# PlasticAnalytics: A Deep Learning-Powered Spectral Library and Analytical Suite

**DOI:** 10.1021/acs.est.6c01309

**Published:** 2026-05-15

**Authors:** Dr.Joseph M. Levermore, Professor Frank J. Kelly, Dr.Stephanie L. Wright

**Affiliations:** Environmental Research Group, MRC Centre for Environment and Health, School of Public Health, 4615 Imperial College London, London, W12 0BZ, United Kingdom

**Keywords:** microplastics, raman spectroscopy, fourier transform infrared spectroscopy, spectral library, deep residual network, out-of-distribution detection

## Abstract

PlasticAnalytics provides an automated workflow that addresses key bottlenecks in vibrational spectroscopic analysis of microplastics by Raman spectroscopy and Fourier transform infrared spectroscopy (FTIR). The preprocessing framework integrates an iterative asymmetric penalized least-squares (i-arPLS) baseline correction algorithm optimized for spectra with complex environmental backgrounds, coupled with a hybrid rule-based and machine learning framework that automatically removes spurious peaks (cosmic rays and CO_2_) while handling resampling, normalization, and smoothing. A complementary machine learning module identifies and removes substrate spectra in spectral images, ensuring that downstream classification operates only on particulate-derived signals. The pipeline combines these steps with a deep residual network and an uncertainty-aware quality-control classifier trained on virgin, consumer, and environmentally weathered plastic spectra, achieving classification accuracies of 96.9% (Raman) and 97.9% (FTIR) and matching or exceeding existing architectures. For spectral imaging, automated background removal and high-speed inference reduced processing time by over 90%, from more than 200 min (Raman) and 800 min (FTIR) to under 7 min in both cases. PlasticAnalytics supports the major instrument platforms and file formats, providing a scalable, reproducible pipeline for environmental microplastic analysis.

## Graphical abstract

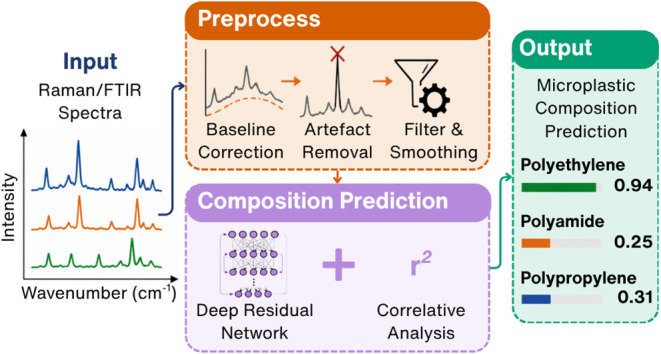

## 1 Introduction

Microplastic pollution is a persistent particulate contaminant detected in aquatic, ambient air, terrestrial, and biological samples.^1^ Visual inspection, commonly used for initial screening of samples for suspected microplastics, is highly error-prone, yielding false-positive rates of 20–100%.^2–4^ To verify suspected microplastics, obtain representative abundance estimates, and minimize analytical error, researchers should chemically characterize 10–50% of the total particles or filter area, as analytical error decreases with increasing analyzed sample fraction.^5–8^

Raman and Fourier transform infrared (FTIR) spectroscopy remain the gold standards for this chemical categorization. Conventional workflows span from manual particle-by-particle interrogation to computer-vision-guided spectral imaging.^7–12^ To meet demands for high-throughput analysis, cutting-edge technologies have emerged: Quantum Cascade Laser (QCL)-based Laser Direct Infrared (LDIR) imaging enables ultrafast screening, while Stimulated Raman Scattering (SRS) allows rapid nanoplastic detection in flow.^13^ Additionally, Optical Photothermal Infrared (O-PTIR) spectroscopy and hyphenated approaches such as asymmetrical flow field-flow fractionation coupled with Raman microspectroscopy (AF4–RM) extend analytical capabilities toward submicron resolution and nanoscale particle characterization in complex matrices.^12,14^

Despite these instrumental advancements, computational processing remains a critical bottleneck.^15^ Environmental microplastics exhibit altered spectral fingerprints due to additives, weathering, and biofouling.^3,16–20^ Commercial software struggles with this complexity; proprietary databases rely heavily on pristine standards, creating a severe diagnostic mismatch.^18,21,22^ Furthermore, their rudimentary univariate algorithms and standard baseline corrections frequently amplify noise and cause false assignments, forcing laborious manual integration.^23–25^

While open-source libraries (e.g., OpenSpecy and SLoPP) offer alternatives, they rely on univariate matching methods that struggle with high intergroup similarity.^22,26–28^ For example, using the Pearson correlation coefficient (PCC) to compare spectral intensities at corresponding Raman shifts can yield a correlation of up to 0.78 between polyamide (PA) and polyethylene (PE).^28^ Traditional machine learning (ML) algorithms (e.g., SVMs and decision trees) improve classification performance but typically require manual feature engineering and remain vulnerable to baseline distortions.^29–31^ In contrast, deep learning models (DLMs), particularly convolutional neural networks (CNNs), autonomously learn hierarchical features.^32,33^ By capturing multidimensional patterns simultaneously, CNNs are highly robust to the noise and spectral shifts of environmental degradation, consistently achieving 94 to 97% accuracy and exceeding traditional ML by 4 to 13%.^30,34–36^

Yet, deep learning approaches in this space remain largely siloed. To bridge this gap, we present PlasticAnalytics, a consolidated platform integrating automated preprocessing, a curated spectral library, and deep learning for the Raman and FTIR analysis of microplastics. Powered by a novel DLM, the suite enhances the prediction speed and accuracy across 26 polymer classes, supporting high-throughput workflows for individual spectra and spectral imaging.

## 2 Materials and Methods

### 2.1 Software Architecture and Data Management

Accessible at https://plasticanalytics.co.uk, PlasticAnalytics is a cross-platform spectral analysis suite (React web and Python desktop) integrating all of the models described herein. It supports over 20 standard file formats (e.g., .txt, .csv, .spa, and.wdf) and enforces metadata standards for user uploads. Figure 1 outlines the methodological workflow.

**Figure 1:**
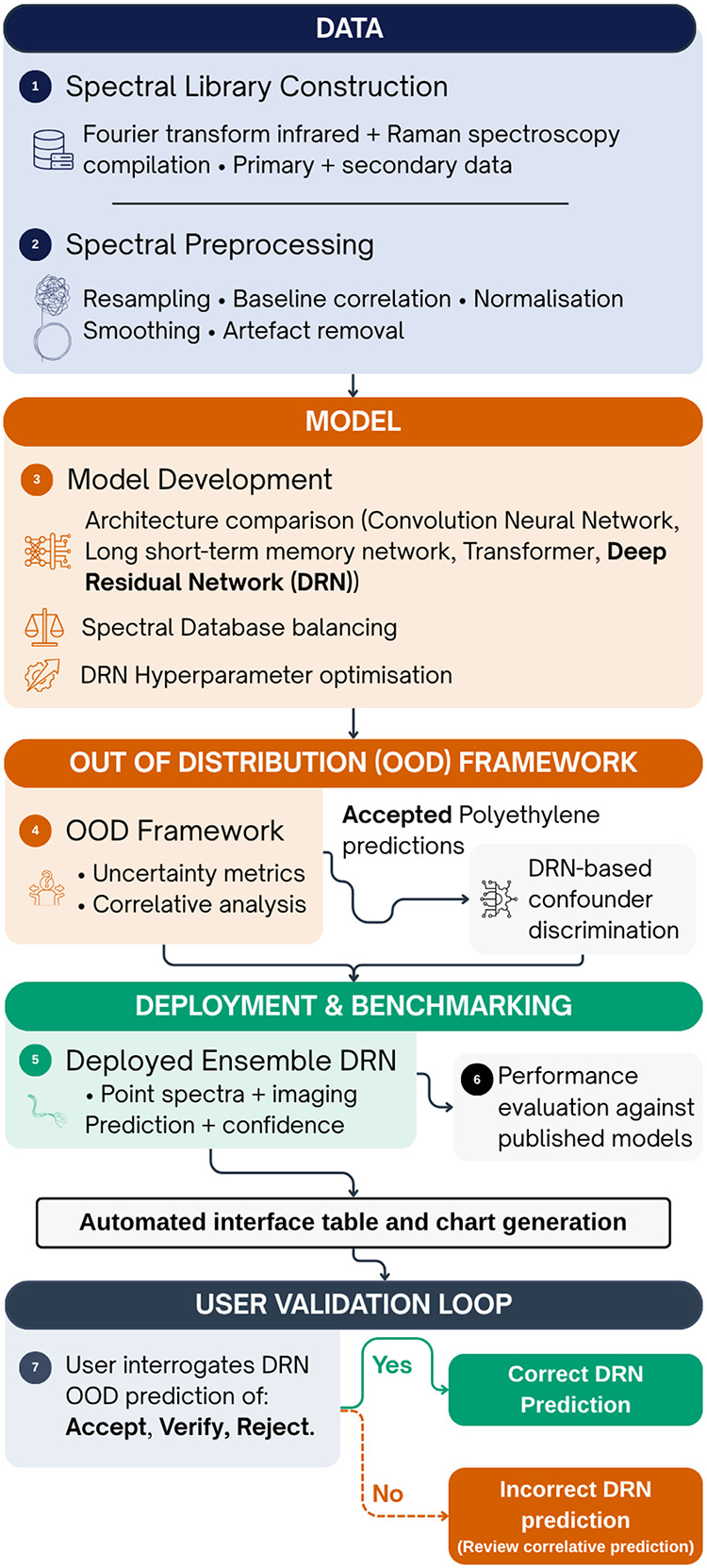
Methodological workflow for spectral data analysis and plastic classification. The pipeline includes data compilation, model development and optimization, an out-of-distribution (OOD) filtering framework (with an additional step for spectra predicted as polyethylene, PE), and deployment benchmarking. The final stage involves human-in-the-loop validation, conducted via the PlasticAnalytics Application user interface, which allows users to review and confirm classification results.

### 2.2 Spectral Preprocessing Pipeline

Spectral candidates were obtained from public repositories or collected by the authors (Tables S4 and S5). All spectra were subjected to a standardized, automated preprocessing workflow, described in detail below.

#### 2.2.1 Resampling and Baseline Correction

Spectra were resampled via linear interpolation to 1000 intensity values (Raman: 200–3200 cm^–1^; ATR-FTIR: 4000–400 cm^–1^). Where spectra did not cover the full target wavenumber range, uncovered regions were padded using the mean class intensity. Baseline correction employs an iterative asymmetric penalized least-squares (i-arPLS) algorithm with adaptive convergence ratios (SI S-2.3), selected for its ability to isolate baselines obscured by fluorescence from environmental fouling, outperforming standard polynomial methods.^24,25^

#### 2.2.2 Spurious Peak Removal

FTIR signals associated with CO_2_ absorption and Raman cosmic ray artifacts were removed prior to normalization to prevent artifact-driven suppression of polymeric signals. A hybrid framework was used to identify cosmic ray artifacts: narrow rays (full width at half-maximum (FWHM) < 1.5 cm^–1^) were filtered via the rule-based criteria detailed in SI S-2.4.2, while broader rays were handled by a Gradient Boosting (GB) classifier. GB was selected over Random Forest (RF) via 5-fold cross-validation, and all confirmed rays were removed using Piecewise Cubic Hermite Interpolating Polynomial (PCHIP) interpolation (SI S-2.4).^37^ Atmospheric CO_2_ absorption peaks (2288–2392 cm^–1^) were excised using PCHIP interpolation with sigmoid-based weighting functions to prevent baseline oscillations (SI S-2.5). Following artifact removal, spectra underwent maximum intensity normalization and Savitzky–Golay smoothing.^28^

### 2.3 Machine Learning Classification Engine

#### 2.3.1 Data Set Construction and Balancing

Spectra were compiled from public repositories^21,22,27,38–46^ and in-house sources. Based on an ablation study evaluating augmentation strategies and data set scaling (SI S-2.8.1.3 and SI S-3.3.1.1), a minimum threshold of 50 original spectra per class was adopted for DLM training, with underrepresented classes reserved for PCC prediction. Classes meeting this threshold were augmented to 8000 spectra per class via i-arPLS-based augmentation, yielding final data sets of 80,000 Raman (10 classes: acrylonitrile butadiene styrene (ABS), PA, polycarbonate (PC), PE, poly­(ethylene terephthalate) (PET), polypropylene (PP), polystyrene (PS), poly­(vinyl chloride) (PVC), poly­(methyl methacrylate) (PMMA), and poly­(tetrafluoroethylene) (PTFE)), and 128,000 ATR-FTIR spectra (16 classes: high-density PE (HDPE), low-density PE (LDPE), PA, PE, polyester (PES), PET, PP, PP-CaCO_3_, PP-flame-retardant (PP-FR), PP-glass fiber (PP-GF), PP-green pigment (PP-GP), PP-red pigment (PP-RP), PP-white pigment (PP-WP), PS, polyurethane (PU), and PVC).

#### 2.3.2 Model Architecture and Training

Five architectures were evaluated: CNN, LSTM, Transformer, Inception-CNN, and a deep residual network (DRN; SI S-2.8.2; Table S6).^36,47–50^ The DRN was selected for its high accuracy combined with a reduced parameter count, incorporating Squeeze-and-Excitation (SE) blocks and multiscale kernel convolutions.^51^ Hyperparameters were optimized via random search under stratified 5-fold cross-validation, with final model selection by highest mean test accuracy (SI S-2.8.2.1). Predictions were generated through a stratified 10-fold ensemble.

#### 2.3.3 Out-of-Distribution Detection

A three-tier framework was implemented to prevent the DLM from assigning high confidence to out-of-distribution (OOD) spectra.^52,53^ First, spectra are PCC-screened against a nonpolymer reference library (SI S-2.8.3.1).^54–60^ Second, an RF model trained on DRN uncertainty features (e.g., predictive entropy, Mahalanobis distance; SI S-2.8.3.2) classifies residual spectra as Accept, Verify, or Reject. A third stage was implemented for Raman analysis to resolve spectral overlap between PE and confounding slip additives (e.g., stearic acid) using a disambiguation framework evaluating three deep learning architectures (DRN, Advanced U-Net, and U-Net; SI S-2.8.3.3).^61,62^ The full framework was evaluated on held-out spectra per modality using ROC-AUC, precision recall, and classification accuracy.

#### 2.3.4 Benchmarking Against Reference Methods

The DRN and OOD framework were benchmarked against PCC spectral matching and six established models: ResNet-Inception,^63^ CoordConv-Inception,^63^ CPL Compound Mixtures, CPL Compound Class,^64^ a 1D-CNN,^30^ and an SE-improved ResNet18.^65^ Evaluation was performed on 953 FTIR and 129 Raman OOD-filtered test spectra with manually validated ground truth labels, assessed by composition accuracy, rank-weighted score, McNemar’s test, and mean inference time, including comparison against Bio-Rad’s KnowItAll software (SI S-2.8.4).^40^

### 2.4 Spectral Imaging and Substrate Removal

In spectral imaging, nontargeted acquisition yields a large proportion of background substrate spectra that must be excluded prior to classification. A semisupervised ensemble model (SSEM) was developed, combining an RF classifier with PCA-informed K-means clustering (SI S-2.9). Model training utilized manual annotations across 17 FTIR and 22 Raman spectral images, yielding 127,675 labeled pixels. The RF classifier was trained on spectral principal components and engineered statistics (peak intensity, variance, skewness), with hyperparameters optimized via grid search using F1-score (SI S-2.9.1.2). The pretrained SSEM was subsequently evaluated across 25 Raman and 53 FTIR unseen spectral images for automated substrate identification and masking using weighted ensemble confidence scoring. The impact of substrate removal on downstream efficiency was quantified by comparing the chemometric and deep learning classification times with and without background masking. Following substrate removal, retained spectra were classified using the proposed pipeline and mapped for morphological characterization.

## 3 Results and Discussion

### 3.1 Plastic Spectral Library

The spectral reference library comprised 2569 Raman spectra spanning 157 unique material classes and 9550 ATR-FTIR spectra encompassing 122 material classes (Tables S4 and S5). Raman spectra included common thermoplastics (ABS, PA, PC, PE, PET, PMMA, PP, PS, PTFE, and PVC), PU, polyesters, rubbers, biopolymers (e.g., PLA and PHB), and natural materials such as cellulose, wool, and chitin. The FTIR library similarly covered these polymer classes and was further expanded to include a wider diversity of copolymers, additives, natural fibers, and environmental analogues. Across both instrument modalities, the data sets captured a range of sample conditions, including virgin and consumer materials (Raman: 78.3%; FTIR: 47.8%), weathered samples (Raman: 7.8%; FTIR: 22.8%), biofouled materials (FTIR: 29.4%), and other reference materials of unspecified condition (Raman: 13.9%). Raman acquisitions were predominantly collected at 532 nm (62.5%), followed by 785 nm (24.1%) and 633 nm (13.4%).

Prior to ML model training, an ablation study on preprocessed spectra showed that reducing the number of original seed spectra decreased predictive accuracy. Mann–Whitney U tests confirmed a significant improvement when increasing the number of original spectra from 20 to 50 for each augmentation strategy (Original: *p* = 0.028; SMOTE: *p* = 0.028; i-arPLS: *p* = 0.016). Consequently, a minimum threshold of 50 original spectra per class was applied to both the Raman and FTIR data sets. This yielded 10 Raman and 16 ATR-FTIR classes with sufficient spectral volume to train the DLM, with the mean spectrum and standard deviation for each polymer class presented in Figures 2 and S2 (see SI S-3.1).

**Figure 2:**
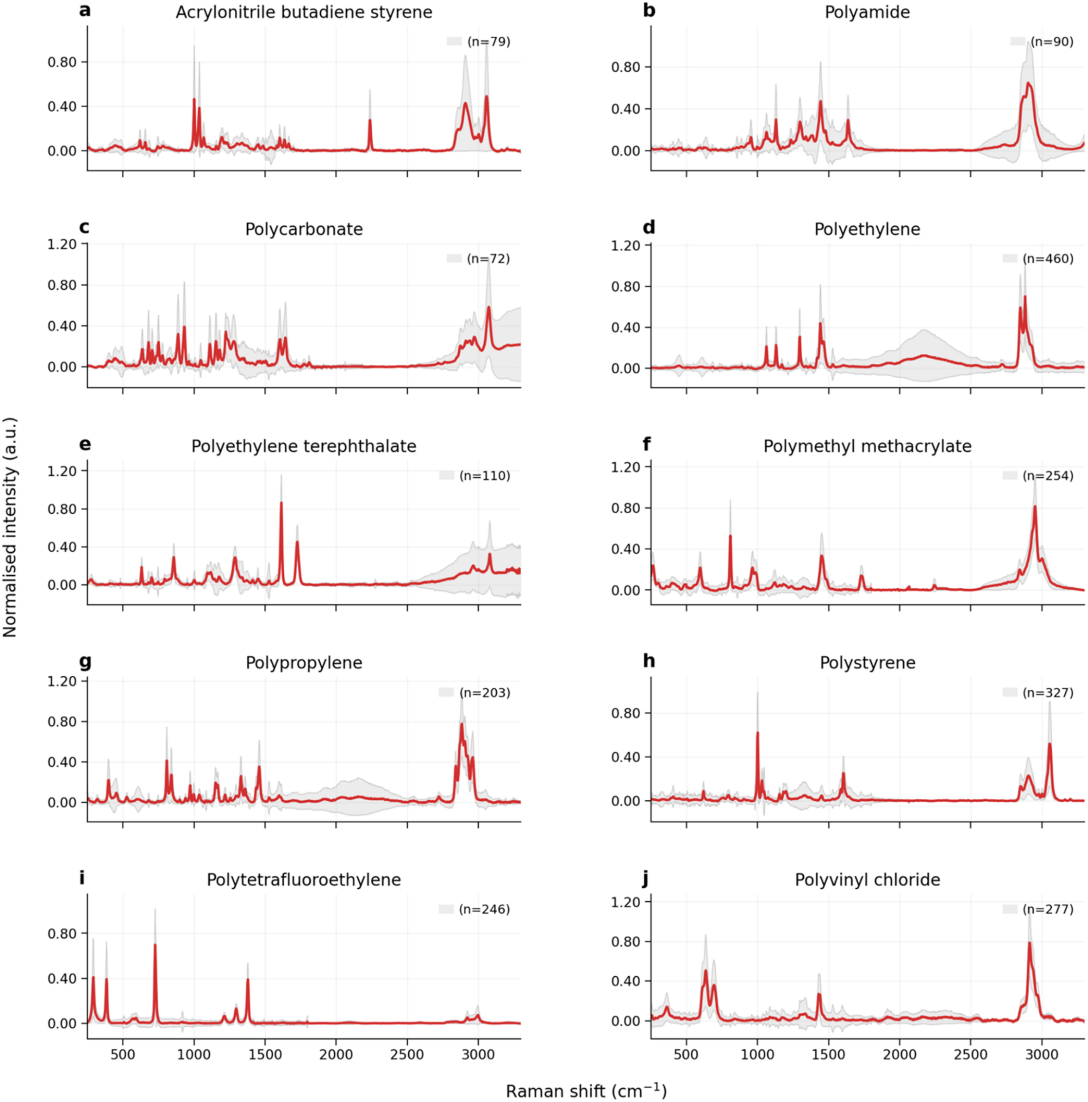
Raman spectra for 10 polymer classes in the spectral library used for deep learning, shown prior to data augmentation. Spectra have undergone resampling, intensity normalization, baseline correction, and cosmic ray removal. Each panel represents one polymer type: acrylonitrile butadiene styrene (a), polyamide (b), polycarbonate (c), polyethylene (d), poly­(ethylene terephthalate) (e), poly­(methyl methacrylate) (f), polypropylene (g), polystyrene (h), poly­(tetrafluoroethylene) (i), and poly­(vinyl chloride) (j). The red line indicates the mean spectrum for each class, while the gray shaded region represents ± 1 standard deviation. Spectra are derived from primary and secondary data sources.

Having established this minimum real-data requirement, three augmentation methods were then applied to expand the number of spectra per class. No statistically significant difference in performance was observed when increasing the augmented spectra from 500 to 8000 (Wilcoxon, *p* > 0.15). However, within a controlled scaling trajectory using a fixed initialization (seed = 50), F1 scores increased with the number of augmented spectra, improving from 0.913 to 0.929 (original), 0.946 to 0.965 (i-arPLS), and 0.935 to 0.947 (SMOTE) as augmentation increased from 500 to 8000 spectra per class. These values reflect within-seed scaling behavior and are not directly equivalent to the absolute best-performing configurations reported in Table S13B, which are obtained by optimizing across all tested seed sizes and augmentation targets (e.g., SMOTE: F1 = 0.958 at seed = 61). Despite the lack of statistical significance across this trajectory, the highest performance under the fixed-seed scaling regime was observed for i-arPLS at 8000 spectra per class (F1 = 0.965, accuracy = 96.2%), which also corresponds to the global optimum for this method. Therefore, classes meeting the minimum original spectral threshold (*N* = 50) were expanded to 8000 spectra per class.

### 3.2 Spurious Peak Removal Evaluation

The hybrid cosmic ray removal (CRR) framework, comprising rule-based detection and an ML enhancement stage, was evaluated for its ability to isolate sharp artifacts from genuine Raman bands. The initial rule-based pipeline, leveraging fwhm, derivative, and statistical outlier criteria, identified 49.1 ± 12.2% of artificially introduced cosmic rays (2002 injected rays across 104 reference spectra). Detections primarily missed broader (FWHM 0.75 ± 0.21 cm^–1^) and lower-amplitude (9.5 ± 3.4 times higher than the local background) events. To minimize false positives in Raman band detection, classification thresholds were optimized via grid search. The optimal band sharpness, derivative, and z-score thresholds were 1.0, 0.2, and 25.0, respectively. A band was classified as positive only if both the sharpness and derivative criteria were satisfied, yielding a precision of 98.7%, recall of 99.3%, F1-score of 0.990, and mean FPR of 6.0%.

To recover cosmic rays missed by the rule-based stage, RF and GB classifiers were hyperparameter optimized (Table S11) and evaluated. The GB model outperformed the RF alternative, achieving a weighted F1-score of 0.975 ± 0.002, recall of 97.46 ± 0.18%, and precision of 97.47 ± 0.19%, while requiring approximately half the optimization time (29,677 s vs 54,616 s). Consequently, GB was selected for integration. Feature importance analysis identified fwhm (68.8%), derivative sharpness (8.9%), and asymmetry (7.8%) as the primary predictors for successful classification (SI S-3.2).

When deployed as a hybrid pipeline, the ML classifier recovered an additional 50.9 ± 12.2% of cosmic rays missed by the rule-based stage, yielding a mean final detection rate of 99.9 ± 0.5% across validation spectra and an aggregate recall of 99.95% overall (1 of 2002 injected cosmic rays missed). Confirmed detections were corrected using PCHIP interpolation, which effectively preserved spectral peak shapes while providing operator-configurable robustness for extreme or out-of-distribution artifacts (Figure 3).

**Figure 3:**
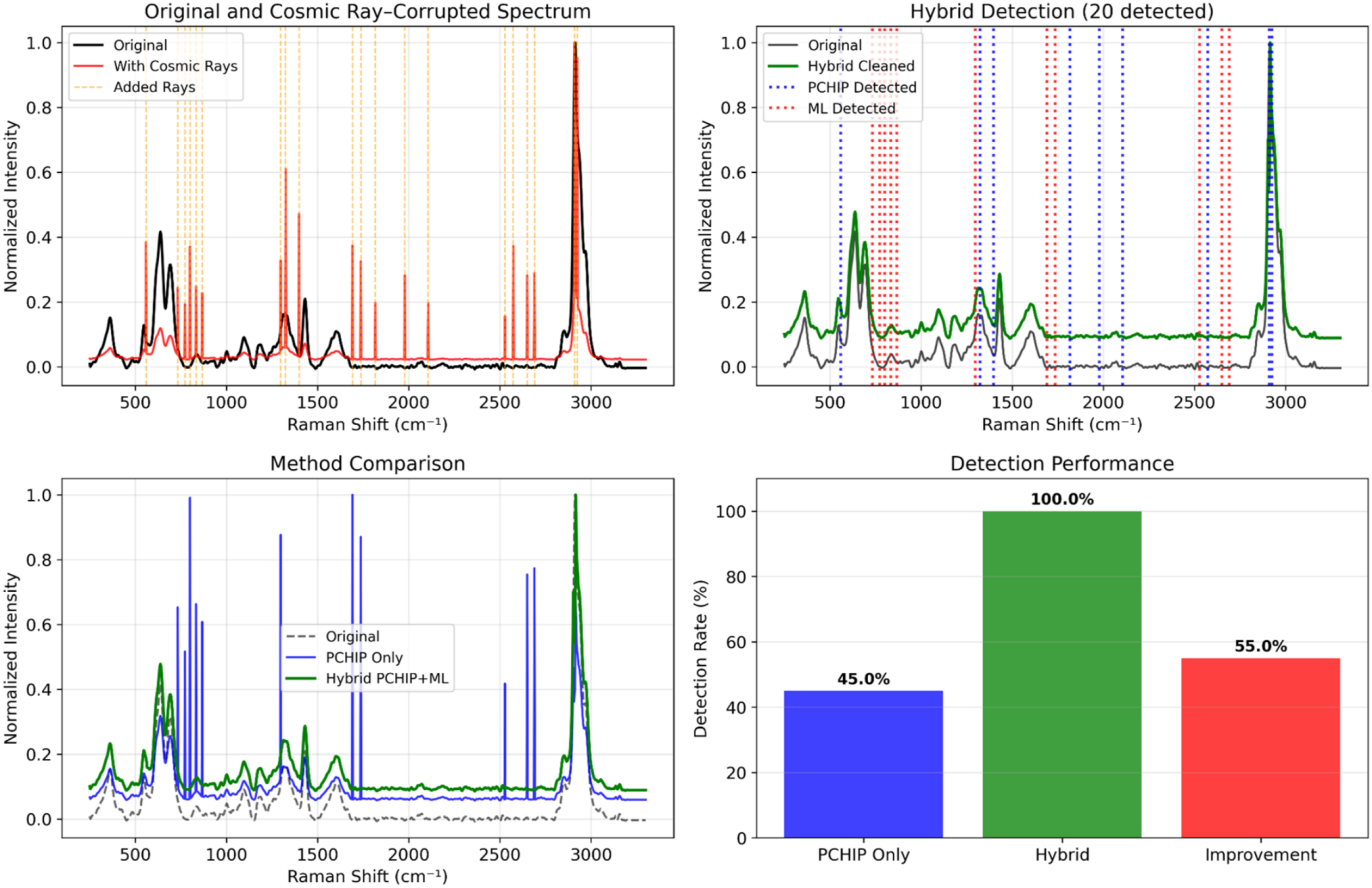
Cosmic ray removal on an example Raman spectrum of poly­(vinyl chloride) (PVC) using the proposed hybrid framework combining rule-based detection, machine learning (ML), and Piecewise Cubic Hermite Interpolating Polynomial (PCHIP) correction. Twenty synthetic cosmic rays, spanning a range of positions, widths (fwhm 0.31–1.15 cm^–1^), and amplitudes (5.7–19.5× local background), were injected across the spectrum to evaluate performance (detailed in Table S12). Using a standard fwhm threshold of 1.0 cm^–1^, the rule-based detection pipeline identified 9 of the 20 injected cosmic rays, with the remaining 11 detected by the ML enhancement step. The hybrid rule-based+ML correction successfully detected and removed all 20 cosmic rays, achieving 100% detection.

Out of 231 ATR-FTIR spectra evaluated, 67 contained polymeric vibrational modes adjacent to the CO_2_ absorption bands; these features, including the nitrile stretch in ABS (∼2240–2260 cm^–1^), were preserved following CO_2_ removal. The algorithm achieved >97% average intensity reduction at both target wavelengths across 100 reference spectra. Although this feature lies close to the lower CO_2_ absorption band at 2288 cm^–1^, the PCHIP-based excision and buffer filtration strategy preserved the nitrile signal without truncation. Effective removal of CO_2_ absorption features was achieved across a range of initial band intensities, spanning 0.20–0.90 and 0.15–0.71 for synthetic bands centered at 2288 cm^–1^ and 2392 cm^–1^, respectively. Using optimal parameters (edge buffer = 30, extended region factor = 2.0, 12 baseline sampling points, and smoothing factor = 0.98), the method achieved >97% removal efficiency across 100 reference spectra, with an average intensity reduction of 97.8 ± 2.4% at both wavelengths. These results demonstrate robust mitigation of atmospheric CO_2_ interference while preserving the intrinsic spectral characteristics of the underlying polymer matrix (Figure 4).

**Figure 4:**
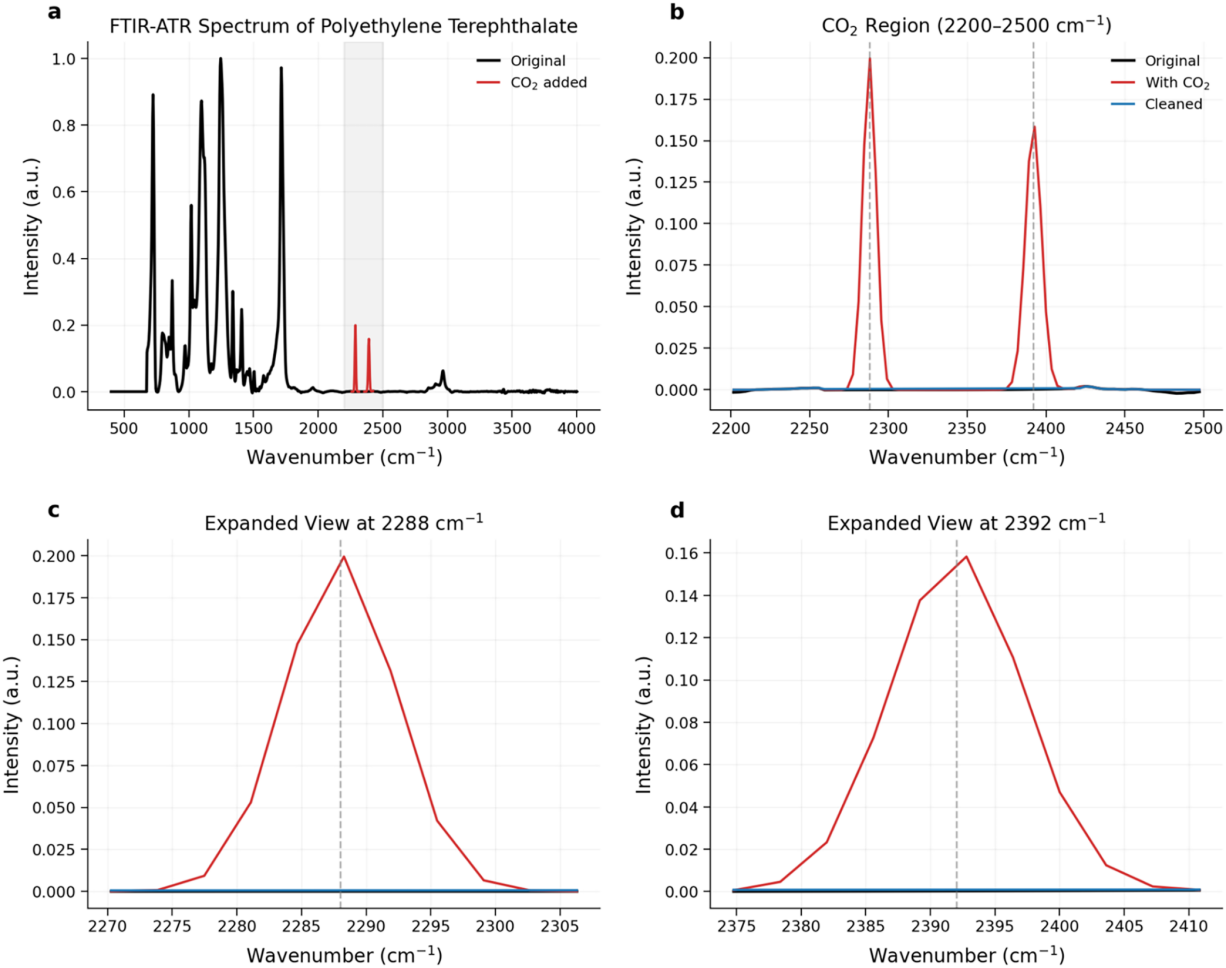
CO_2_ band removal in PET Fourier Transform Infrared Spectroscopy spectra using the proposed cleaning algorithm. (A) Full-spectrum comparison of the original PET spectrum (black), spectrum with synthetic CO_2_ bands added (red), and spectrum post-removal (blue). The CO_2_ target region (2200–2500 cm^–1^) is highlighted in orange, with boundary markers at 2288 cm^–1^ and 2392 cm^–1^. (B) Difference spectra illustrating the added CO_2_ signal (red), the original spectrum (black), and the residual after cleaning (blue). (C–D) Zoomed views of the CO_2_ region showing characteristic double peaks at 2288 and 2392 cm^–1^, respectively. The expanded views demonstrate near-complete removal of the synthetic CO_2_ bands in both regions. Local removal efficiencies exceeded 99% across both peak regions, surpassing the 80% target threshold. These results indicate that the algorithm effectively suppresses atmospheric CO_2_ interference while preserving the intrinsic spectral features of PET.

### 3.3 Chemometric Analysis

#### 3.3.1 Model Selection

The DRN was benchmarked against the four previously described architectures. For FTIR data, the DRN achieved the highest test accuracy (99.99 ± 0.01%), outperforming the Standard CNN (99.87 ± 0.09%) and Transformer (99.55 ± 0.10%). For Raman spectra, DRN achieved the second-best performance (99.56 ± 0.18%) compared to the Standard CNN (99.63 ± 0.14%), with an accuracy difference of 0.07% and overlapping standard deviations indicating comparable performance (Figure S7). Importantly, the DRN exceeded both the Transformer (99.16 ± 0.29%) and LSTM (42.30 ± 23.88%). Consequently, the DRN was selected as the foundational model; it delivered superior or equivalent predictive performance across both spectral domains while requiring approximately half the parameters (2.16 M vs 4.20M) of the Standard CNN, indicating significantly improved computational efficiency (Figures S6, S7 and Table S14).

The improved predictive capability of this DRN, consistent with prior high-accuracy Raman spectroscopy applications,^66^ is attributed to the integration of residual blocks.^67–70^ By facilitating gradient flow via skip connections, the DRN mitigates the vanishing gradient problem encountered by deeper traditional CNNs.^67,70,71^ Unlike data-heavy transformers or standard networks, this architectural efficiency enables the simultaneous extraction of low- and high-level spectral features without overfitting, making DRNs highly optimal for complex spectral data.^72–74^

#### 3.3.2 Hyperparameter Optimization

The DRN was optimized via a random hyperparameter search of 30 configurations, evaluated under stratified 5-fold cross-validation. This search did not identify a single superior architecture; instead, it revealed a plateau of statistically equivalent, high-performing models across both Raman and FTIR data sets (see SI S-3.3.3). The nominally top-performing configuration achieved mean test accuracies of 99.64% (95% CI: 99.61–99.67%) for Raman and 99.98% (95% CI: 99.97–99.99%) for FTIR, but these values were not statistically distinguishable from other high-performing configurations, with performance differences of only 0.003% for Raman and 0.005% for FTIR (pairwise Wilcoxon tests with Bonferroni correction, all *p* > 0.05; Tables S16 and S17).

The model achieved high accuracy on in-distribution (ID) Raman and FTIR spectra (defined as polymer classes present in the training set). However, its real-world utility in a desktop or web-based suite hinges on its ability to identify OOD inputs, a critical aspect often overlooked in prior work.^52^ When OOD detection relied solely on the DRN model’s prediction confidence, the model frequently misclassified OOD spectra while assigning high confidence to these incorrect predictions (e.g., a cotton spectrum classified as PE with a confidence of 95.0 ± 3.0%). Mean prediction confidences for OOD spectra were 95.0 ± 12.1% (FTIR) and 87.8 ± 17.4% (Raman), compared to 99.99 ± 0.13% and 99.8 ± 1.3% for ID spectra. The small and overlapping margins between these distributions highlight the model’s prediction overconfidence for OOD spectra and underscore the need for a dedicated framework to detect and manage such inputs (see SI S-3.3.4.5).

#### 3.3.3 Out-of-Distribution Detection Framework

To establish an OOD detection framework, we first focused on PE and the spectra commonly confounded with it (stearic and oleic acid standards; Figure S11). These spectra exhibit high interclass similarity and compositional overlap with PE and PP (Figure S12), with correlation analysis showing strong linearity across standards and mixtures (mean *r* = 0.97 ± 0.02). Several pairs were perfectly correlated (*r* = 1.00), notably pure stearic acid with its 90:10 mixture and pure oleic acid with its 10:90 mixture. Two distinct clusters of near-perfect correlation (*r* ≥ 0.99) emerged among mixture spectra: one spanning 10–50% stearic acid and another spanning 60–90% stearic acid (Figure S12). This extensive spectral overlap renders univariate thresholding ineffective, motivating the use of ML to map nonlinear decision boundaries between classes. 120 hyperparameter configurations were evaluated for both the DRN and Advanced U-Net, while the baseline U-Net proposed by Lim et al.^61^ was implemented using its published default parameters. The optimal DRN achieved a global average test F1-score of 0.9469, exceeding that of the baseline U-Net (0.9415; Tables S20 and S21). The DRN also demonstrated superior cross-validation performance (0.9558 ± 0.0018 vs 0.9524 ± 0.0017), indicating improved generalization. While the DRN achieved the highest absolute performance, cross-validation confirmed that it remained statistically indistinguishable from the next four top-performing DRN configurations (*p* > 0.05).

Class-level metrics highlighted model-specific limitations. Predictions for pure stearic acid were the least accurate across all models (mean F1-scores: DRN 0.506 ± 0.062; Advanced U-Net 0.380 ± 0.029; U-Net 0.435 ± 0.067; Figures S13–S15). Analysis of misclassifications revealed that the majority (∼60%) were assigned to the 90:10 stearic acid–oleic acid mixture, reflecting the perfect linear correlation (*r* = 1.00) between these spectra. Specifically, normalized predicted probabilities for the 90:10 mixture were 60, 74, and 60% for DRN, Advanced U-Net, and U-Net, respectively, indicating that the models systematically predicted the near-identical mixture rather than the true pure standard. At the optimal postablation configuration, the stearic acid F1-score increased to 0.8447 (Table S20).

To assess whether these limitations arose from insufficient training data or fundamental spectral indistinguishability, an ablation study systematically varied both the number of unique spectra (20–1500) and total augmented data set size (1000–8000). Across 105 training scenarios (35 per architecture), performance scaled directly with seed size, with macro-F1 rising from 0.603 (20 original spectra, 6000 augmented per class) to 0.959 (1500 original spectra, 1000 augmented per class), confirming that sufficient data and augmentation are critical for resolving challenging, overlapping spectral classes. At the optimal configuration, the DRN outperformed the published U-Net on 11 of 14 classes (mean per-class DRN: F1 = 0.947 ± 0.049; U-Net: 0.942 ± 0.051). While all evaluated architectures achieved comparable overall performance, the largest gains for DRN were observed in spectrally ambiguous mixture classes, where it achieved up to 0.023 higher F1 than the U-Net. Overall, the DRN demonstrated superior performance across the class spectrum and was therefore selected as the final classifier in the OOD pipeline for differentiating PE and confounding spectra, outperforming the published U-Net architecture.^62^

Following selection of the DRN as the final PE disambiguation classifier, the model was integrated into a three-tier framework combining PCC screening, RF-based uncertainty filtering, and DRN-based PE disambiguation to identify and remove OOD spectra, ensuring that only ID data are classified. On held-out data sets (Raman *n* = 104; FTIR *n* = 104), a dual-threshold approach (Raman: τ_low_ = 0.10, τ_high_ = 0.90; FTIR: τ_low_ = 0.45, τ_high_ = 0.70) achieved a strict, fully automated classification accuracy of 81.0% for Raman and 52.4% for FTIR. Crucially, the framework demonstrated a strong safety profile, with no true OOD spectra misclassified as ID for Raman (0/32 false acceptances) and only a small fraction passing for FTIR (2/52 false acceptances). For ambiguous spectra routed to the “Verify” tier, subsequent human-in-the-loop manual review increased the final postverification accuracy to 100.0% for Raman and 61.9% for FTIR. Overall, this demonstrates a secure pipeline for Raman spectroscopy, while FTIR performance was limited by distribution shifts between training and held-out OOD samples (Kolmogorov–Smirnov test, *p* = 0.003), highlighting the need for broader or more representative FTIR spectral libraries to fully capture OOD diversity.

#### 3.3.4 Prediction Classification Comparison

The ensemble DRN was benchmarked against six established ML architectures and the PCC reference library for both Raman and FTIR spectra. On ID Raman spectra (*n* = 129), the DRN achieved the highest composition accuracy (96.9%, mean confidence >99%), outperforming the 1D-CNN proposed by Qin et al.^30^ (96.1%), the SE-improved ResNet18 by Huang et al.^65^ (93.8%), and PCC (77.5%), with consistent gains in chemical family accuracy (97.7%) and rank-weighted score (97.3%). For FTIR spectra (*n* = 953 ID-accepted polymer spectra) from pristine laboratory polymers, the DRN reached 97.9% accuracy with mean confidence 99.5 ± 3.4%. Because the data set is largely unweathered, classification was straightforward: three other CNNs (the CPL models proposed by Cooman et al.^64^ and CoordConv-Inception^63^) matched 97.9% accuracy with full prediction agreement. The DRN maintained the most consistent confidence (SD 3.4 vs 6.0% for other CNNs). PCC achieved 96.9% accuracy on accepted spectra, though with lower correlation (90.4 ± 3.5%), demonstrating that even traditional methods perform well on pristine data sets.

McNemar’s tests indicated no statistically significant differences among top CNNs (DRN, the SE-improved ResNet18 by Huang et al.,^65^ the 1D-CNN proposed by Qin et al.,^30^ the CPL models proposed by Cooman et al.,^64^ and CoordConv-Inception^63^) on either instrument spectra, but the DRN consistently ranked highest. Against non-CNN methods, it significantly outperformed PCC on Raman (*p* < 0.0001) and exceeded the SE-improved ResNet18 by Huang et al.^65^ (*p* = 0.031) and the 1D-CNN proposed by Qin et al.^30^ (*p* = 0.004) on FTIR. The DRN’s advantage is most pronounced on challenging environmental Raman spectra. Unlike prior studies requiring manual interpretation,^43^ the DRN provides fully automated, high-confidence predictions. Trained on diverse environmental and weathered spectra, it overcomes limitations of commercial software such as Bio-Rad’s KnowItAll (72.31 ± 14.46% confidence; Table S25), achieving 99.99 ± 0.02% confidence with 100% correct compositional assignments.

DRN and PCC produced equivalent classifications on ID spectra, but DRN is substantially faster: 16.6× on FTIR (7.2 ± 4.4 ms vs 119.5 ± 10.1 ms) and 4.3× on Raman (6.8 ± 3.3 ms vs 28.9 ± 1.9 ms). Unlike PCC, DRN inference time is independent of library size, widening the computational advantage as spectral libraries grow. Combined with the three-tier OOD framework, the DRN provides a robust, efficient approach for plastic spectral analysis, maintaining superior accuracy, confidence, and speed on complex environmental spectra where conventional methods falter.

#### 3.3.5 Spectral Image Analysis

To accelerate spectral image analysis, an SSEM was developed that automatically identifies and removes noninformative substrate (background) spectra, retaining only particle spectra for downstream classification via our chemometric pipeline. By excluding background regions prior to analysis, SSEM reduces the number of spectra requiring compositional classification, thereby improving processing efficiency.

Differences in substrates between the Raman and FTIR spectral images (aluminum foil and silver membrane, respectively) resulted in SSEMs with distinct optimized hyperparameters (Table S26). Despite these differences, 10-fold cross-validation confirmed that both models performed well, achieving mean accuracies of 99.6 and 98.8% for Raman and FTIR background spectra, respectively. Performance was consistent across folds, with accuracy (Raman: 99.59 ± 0.07%; FTIR: 98.78 ± 0.12%), precision (Raman: 99.62 ± 0.14%; FTIR: 98.85 ± 0.20%), and recall (Raman: 99.78 ± 0.05%; FTIR: 99.36 ± 0.11%). The influence of hyperparameters for Raman and FTIR background detection models on recall, precision, and accuracy is displayed in Figures S16 to S17.

Feature importance analysis indicated that predictions were primarily driven by spectral features, 62.3% for Raman and 52.9% for FTIR, while spatial location contributed minimally at 2.1 and 1.8%, respectively. This confirms that the optimized SSEM identifies background spectra based on chemical signatures rather than spatial information.

To assess the model’s performance on data sets of varying scales, the aluminum foil- and silver membrane-trained substrate identification models were applied to 25 Raman and 53 FTIR images with sizes ranging from 320 to 1,257,095 pixels. The Raman image data set was divided into two cohorts: large images (Group 1; *n* = 12; mean size, 111,100 ± 105,500 spectra) and small images (Group 2; *n* = 13; mean size, 960 ± 660 spectra). By contrast, FTIR images were consistently 16,384 spectra in size, reflecting the acquisition parameters reported by Weisser et al.,^75^ who collected images of identical dimensions. The Raman substrate constituted 86.2 ± 6.6% and 44.2 ± 15.7% of the image areas for Group 1 and Group 2, respectively, while for FTIR images the substrate was found to constitute 74.4 ± 15.1%. Similar to prior RF optimization analysis, feature importance analysis identified band intensity as the most critical predictor for both Raman (17.0%) and FTIR (17.7%) models. Overall, engineered spectral features were dominant over PCA-derived and spatial contributions (full breakdown in SI S-3.3.6.1.2).

To quantify the downstream impact of the substrate subtraction preprocessing step, the analysis times for a representative 400,000-spectra image were calculated (SI S-3.3.6.4). When using PCC classification alone without spectral preprocessing, the full chemometric analysis (including baseline subtraction, smoothing, alignment, and prediction) required 212.7 min for Raman and 816.7 min for FTIR.^28^ Incorporating the SSEM as the first step of our image analysis workflow to identify and remove substrate spectra, we found that background pixels constituted 86.2 ± 6.6% and 74.4 ± 15.1% of the image area for Raman and FTIR validation data sets, respectively, substantially reducing analysis time. With chemometric correlative predictions performed only on the remaining sample pixels, analysis times were reduced to 21.7 ± 10.8 min for Raman and 83.3 ± 41.6 min for FTIR using PCC classification. The efficiency gain is most pronounced for DRN methods, where the analysis time for Raman and FTIR data drops to 6.7 ± 3.3 and 6.9 ± 3.4 min, respectively. Substrate masking alone yields a ∼9.8-fold increase in analytical throughput under PCC classification (212.7 to 21.7 min for Raman; 816.7 to 83.3 min for FTIR), reflecting the removal of background pixels prior to chemometric analysis. When combined with DRN inference, the full pipeline is further accelerated, reducing processing times to 6.7 ± 3.3 min (Raman) and 6.9 ± 3.4 min (FTIR), corresponding to overall speedups of ∼32× and ∼118× relative to the unmasked PCC baseline.

### 3.4 Broader Applications and Impact

All models, preprocessing functions, and predictive workflows described herein are integrated into PlasticAnalytics, an accessible cross-platform suite designed to eliminate computational bottlenecks in environmental microplastic research. By consolidating advanced background masking, i-arPLS baseline correction, PCHIP-ML artifact removal, and the DRN classification framework into a single automated pipeline, the platform provides a scalable, high-throughput solution for both single-spectrum and large-area spectral image analysis.

PlasticAnalytics currently supports ATR-FTIR and Raman (532, 633, and 785 nm) spectra, featuring a community-driven architecture that facilitates continuous library expansion. Because the optimal choice between FTIR and Raman depends heavily on the specific analytical context (e.g., sample type, fluorescence, and spatial resolution) rather than one being universally superior, the platform is designed to be instrument-agnostic. By enabling researchers to submit virgin, consumer, and weathered plastic spectra, the platform supports the iterative retraining of the deep learning models, ensuring ongoing adaptability to highly degraded environmental matrices.

To maximize accessibility for the scientific community, the tools are deployed via two modalities: a web-based interface for rapid spectral analysis and database access (https://plasticanalytics.co.uk/spectral-library/database), and a comprehensive desktop application tailored for advanced batch processing of large data sets (https://plasticanalytics.co.uk/software/desktop-application).

### 3.5 Limitations and Future Work

Although the aggregated spectral libraries include 157 Raman and 122 ATR-FTIR polymer types, the current DRN models classify only 10 Raman and 16 ATR-FTIR classes, respectively. In ATR-FTIR, PP dominates with over 1800 spectra, while many rare polymers, such as polybutylene terephthalate, polyimide, and cellulose derivatives, have fewer than 50 entries. Similarly, at 532 nm Raman, PE and PS dominate (more than 200 spectra each), whereas less common polymers, including vinyl copolymers and specialty resins, are sparsely represented, highlighting the strongly imbalanced class distribution.

Within the DRN training data set (10 Raman and 16 ATR-FTIR polymer classes derived from the full spectral libraries), virgin and consumer plastics dominate (Raman: 78.3%; FTIR: 47.8%), with reduced representation of weathered (Raman: 7.8%; FTIR: 22.8%) or biofouled materials (FTIR: 29.4%). Photo-oxidative and biological aging alters spectral fingerprints (e.g., carbonyl formation, fluorescence), meaning degraded microplastics can evade detection by models trained on pristine standards. To partially address this, a reference-aware adaptive clustering framework (SI S-3.3.7) separates spectra into “consensus” (high-correlation, pristine) and “variant” (low-correlation, modified) cohorts, capturing intrapolymer spectral diversity. Subsequent correlative analysis against this library compares degraded spectra to the closest consensus or variant group, accounting for intraclass variation.

Future development of PlasticAnalytics will focus on expanding the spectral library to cover a wider range of weathered and consumer plastics, including materials with diverse additives and copolymers. Users of the desktop and web applications can opt into a model weight updating service, allowing user-validated compositional matches to refine model parameters. They may also contribute spectral data sets to the community-driven repository, supporting iterative retraining and extending predictive performance beyond the current 10 Raman and 16 ATR-FTIR polymer classes. Planned improvements include multilaboratory validation and refinement of uncertainty metrics tailored to environmental matrices. Additionally, limited access to ATR-FTIR data sets for common PE confounders (e.g., slip additives) makes the development of an ATR-FTIR PE disambiguation model a priority for future work.

## Supplementary Material



## Data Availability

The PlasticAnalytics platform, including the web-based spectral libraries and desktop application, is publicly and freely available at https://plasticanalytics.co.uk/. The advanced desktop application is available for purchase at https://plasticanalytics.co.uk/software/desktop-application. Full source metadata for Raman and FTIR spectra are provided in the accompanying supporting information Spreadsheet. All supporting data for this publication are available via the following: https://docs.google.com/spreadsheets/d/1xWNqWZ12XdmVXmTsDSNKNzwe9VKft3_10b4MHN4fa54/edit?usp=sharing.

## Notes

Expanded methodological descriptions, hyperparameter search spaces, model performance tables, ablation analyses, confusion matrices, and spectral library metadata for all components of the PlasticAnalytics pipeline (PDF)
