# Supplementary material for: PlasticAnalytics: A Deep Learning-Powered Spectral Library and Analytical Suite
Source: Environ Sci Technol. 2026 May 15;60(21):15113–23. doi: 10.1021/acs.est.6c01309 (PMC13235552; doi:10.1021/acs.est.6c01309)
Supplement: Supplementary file 1 [file es6c01309_si_001.pdf]

Supporting Information: PlasticAnalytics: A Deep Learning-Powered Spectral Library and Analytical Suite

Dr. Joseph M. Levermore[1*], Professor Frank J. Kelly[1], and Dr. Stephanie L. Wright[1]

[1]Environmental Research Group, MRC Centre for Environment and Health, School of Public Health, Imperial College London, London, W12 0BZ, United Kingdom
*Email: levermorejoseph@gmail.com

Keywords
Microplastics; Raman spectroscopy; Fourier transform infrared spectroscopy; Spectral library; Deep residual network; Out-of-distribution detection.

0. Abstract

PlasticAnalytics provides an automated workflow that addresses key bottlenecks in vibrational spectroscopic analysis of microplastics by Raman spectroscopy and Fourier transform infrared spectroscopy (FTIR). The preprocessing framework integrates an iterative asymmetric penalised least-squares (i-arPLS) baseline correction algorithm optimised for spectra with complex environmental backgrounds, coupled with a hybrid rule-based and machine learning framework that automatically removes spurious peaks (cosmic rays and CO_2) while handling resampling, normalisation, and smoothing. A complementary machine learning module identifies and removes substrate spectra in spectral images, ensuring downstream classification operates only on particulate-derived signals. The pipeline combines these steps with a Deep Residual Network and an uncertainty-aware quality-control classifier trained on virgin, consumer, and environmentally weathered plastic spectra, achieving classification accuracies of 96.9% (Raman) and 97.9% (FTIR) and matching or exceeding existing architectures. For spectral imaging, automated background removal and high-speed inference reduced processing time by over 90%, from more than 200 minutes (Raman) and 800 minutes (FTIR) to under 7 minutes in both cases. PlasticAnalytics supports the major instrument platforms and file formats, providing a scalable, reproducible pipeline for environmental microplastic analysis.

1. Introduction

The Supporting Information includes expanded methodological descriptions, hyperparameter search spaces, model performance tables, ablation analyses, confusion matrices, and spectral library metadata for all components of the PlasticAnalytics pipeline. Full source metadata for Raman and FTIR spectra are provided in the accompanying Supporting Information Spreadsheet.

Table of Contents

0. Abstract **1**
1. Introduction **1**
2. Methods **4**
2.0 Study flowchart 4
2.1 Spectral range selection 6
2.2 Spectrum resampling 6
2.3 Baseline correction – iterative baseline correction 6

2.4 Cosmic Ray Removal 7
2.4.1 Validation Methodology 7
2.4.1.1 Synthetic Cosmic Ray Generation 7
2.4.1.2 Validation Dataset 8
2.4.1.3 Algorithmic Performance metrics 8
2.4.2 Rule-Based Cosmic Ray Identification 10
2.4.2.1 Rule-Based Threshold Optimisation 11
2.4.3 Cosmic Ray Removal 11
2.4.3.1 Linear Interpolation 12
2.4.3.2 Median Interpolation 12
2.4.3.3 Piecewise Cubic Hermite Interpolating Polynomial Interpolation 12
2.4.4 Machine Learning Enhancement 13
2.4.4.1 Training Data 13
2.4.4.2 Classifier Training 13
2.4.5 Detection Framework 13
2.5 Ambient CO2 Identification - FTIR 13
2.5.1 Baseline Reconstruction 14
2.6 Feature scaling 15
2.7 Correlative analysis 15
2.7.1 Correlative analysis for new-spectral-candidate addition by user 15
2.7.2 Spectral Quality Clustering Framework 16
2.7.2.1 Correlation-based Clustering 16
2.7.2.2 Three-Tier Reference Structure 17
2.7.2.3 Divergence Analysis 17
2.8 Machine Learning - Deep Residual Network 17
2.8.1 Creation of the Plastic Raman and FTIR Spectral Library 17
2.8.1.1 Plastics contained in the PlasticAnalytics Database 18
2.8.1.2 Dataset augmentation 23
2.8.1.2.1 Noise Addition 23
2.8.1.2.2 Baseline transformation 23
2.8.1.2.2.1 Linear Baseline Profile 23
2.8.1.2.2.2 Exponential Baseline Profile 23
2.8.1.2.2.3 Quadratic Baseline Profile 24
2.8.1.2.2.4 Sigmoid Baseline Profile 24
2.8.1.2.2.5 Gaussian Baseline Profile 24
2.8.1.2.3 Dataset augmentation strategies 25
2.8.1.3 Ablation Study: Determination of Minimum Original Spectra and Augmentation Target 25
2.8.2 Comparative model architecture evaluation 25
2.8.2.1 Model Selection and Hyperparameter Optimisation 26
2.8.3 Handling of model uncertainty: Out-of-distribution detection 26
2.8.3.1 Correlative Reference Analysis 27
2.8.3.2 Random Forest classifier for OOD detection 27
2.8.3.3 Deep learning discrimination of polyethylene 28

2.8.3.3.1 Hyperparameter Optimisation 28
2.8.3.3.1.1 Training data selection and separation strategy 28
2.8.3.3.2 Model hyperparameter search space 29
2.8.3.3.3 Ablation Study 29
2.8.3.3.4 Evaluation metrics 30
2.8.3.4 Framework Evaluation 30
2.8.4 Prediction classification comparison: Univariate versus Multivariate 30
2.9 Spectral Imaging 31
2.9.1 Substrate Spectra Identification and Removal 31
2.9.1.1 Dataset Training Approach and Substrate Annotation 31
2.9.1.2 Random Forest Substrate Detection Models 31
2.9.1.3 Validation and cross-dataset performance 32
2.9.1.4 Spectral Image Dimension Calculation 32
2.9.1.5 Plastic scope and Microplastic Metadata 33
2.9.1.6 Microplastic Shape and Size 33
2.9.2 Image Inspector 33
2.9.2.1 Maximum intensity 34
2.9.2.2 Integrated intensity 34
2.9.2.3 Correlation Map 34
2.10 Reference library architecture 34
3. Results 35
3.1 Plastic types currently available 35
3.2 Spurious peak removal 36
3.2.1 Cosmic Ray Removal 36
3.2.2 CO2 Removal 38
3.3 Chemometric analysis 38
3.3.1 Machine Learning dataset balancing 38
3.3.1.1 Ablation Study: Data Augmentation and Training Set Size 39
3.3.2 Machine Learning model selection 40
3.3.3 Model architecture and ensemble configuration 43
3.3.3.1 Statistical analysis of top model configurations 46
3.3.4 A Hierarchical Framework for Efficient Out-of-Distribution Detection 48
3.3.4.1 Deep learning discrimination of polyethylene and its confounding counterparts 48
3.3.4.2 Dataset linearity 50
3.3.4.3 Model hyperparameter optimisation 51
3.3.4.4 Model classification evaluation 52
3.3.4.5 Baseline: DRN Ensemble Confidence 55
3.3.4.6 Uncertainty-aware Random Forest classifier 57
3.3.4.7 Final Hierarchical PCC and RF Framework for Out-of-Distribution Detection 58
3.3.5 Prediction classification comparison 59
3.3.6 Spectral image analysis 60
3.3.6.1 Background identification and removal 60
3.3.6.1.1 Semi-Supervised Ensemble Model Architecture 60

3.3.6.1.2 Feature importance for model background prediction 63
3.3.6.1.3 Semi-supervised ensemble model inference performance 64
3.3.6.2 Random Forest classification grid search 65
3.3.6.3 Representative benchmark image selection for throughput analysis 66
3.3.7 Reference-aware adaptive clustering framework 66
3.3.7.1 Attenuated Total Reflection–Fourier Transform Infrared Spectroscopy (ATR-FTIR) – Plastic Spectral Library 67
3.3.7.2 Raman Spectroscopy (532 nm, 633 nm, and 785 nm) – Plastic Spectral Library 68
4. Reference List **69**
4.1 Additional References 73

2. Methods

2.0 Study flowchart

To illustrate the operational logic of the chemometric analysis pipeline proposed here, a comprehensive architectural flowchart of the PlasticAnalytics platform was developed (Figure S1). The schematic outlines the sequential spectral preprocessing steps, including resampling, iterative asymmetric penalised least-squares baseline correction, and Piecewise Cubic Hermite Interpolating Polynomial (PCHIP)–machine learning (ML) artefact removal. It also depicts dataset construction and balancing procedures, including ablation-derived thresholds, together with the hyperparameter optimisation framework used for training the Deep Residual Network (DRN) model architecture.

The flowchart further illustrates the deployment of the trained DRN ensemble classifier in combination with the hierarchical out-of-distribution (OOD) detection framework, followed by correlation-based screening of spectra identified as OOD. Together, these components form the PlasticAnalytics analytical pipeline for automated vibrational spectroscopic analysis of virgin, pristine, and environmentally weathered plastics, including statistical reporting and automated graphical comparison between user spectra and predicted reference spectra.

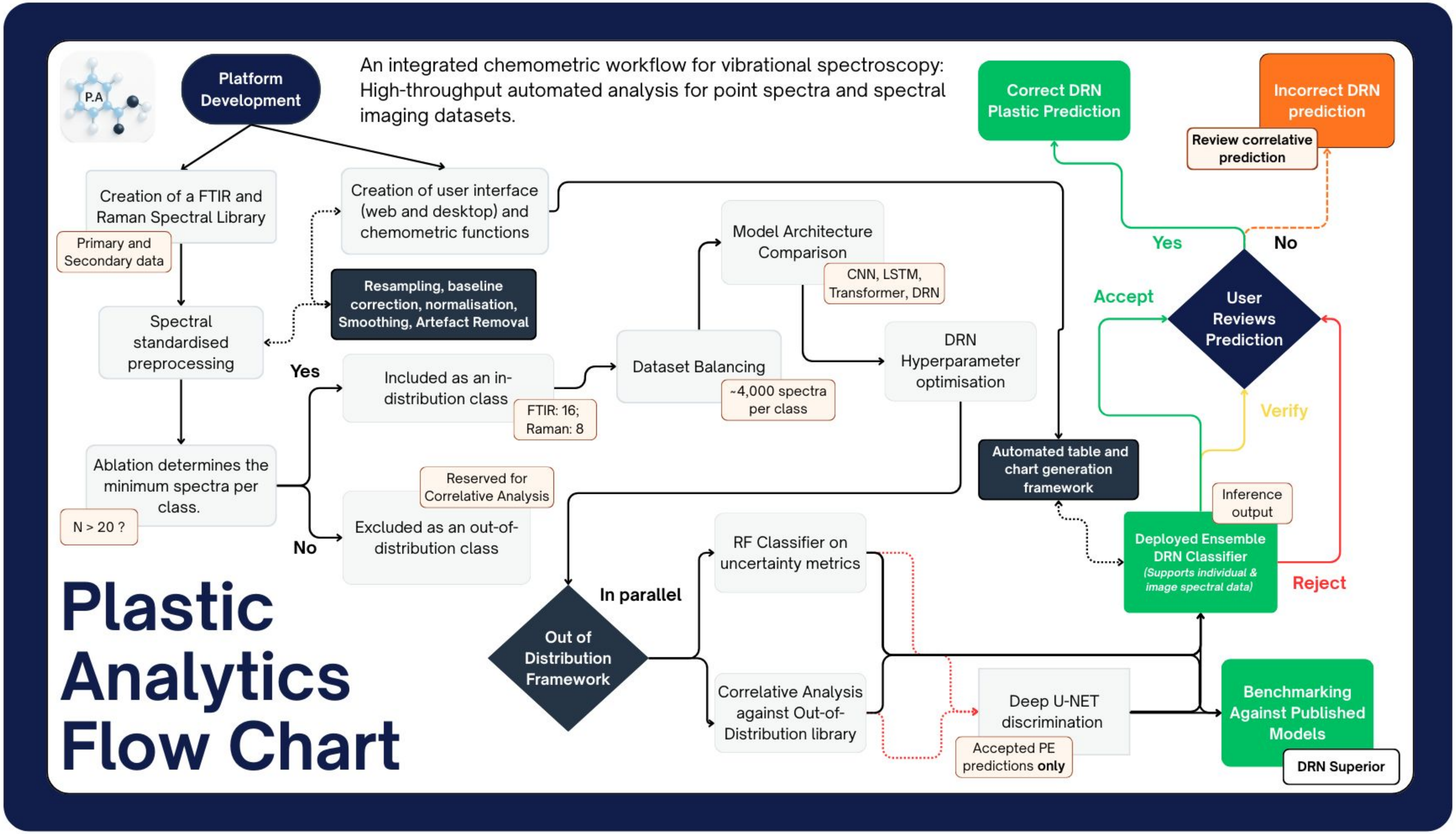

1 **Figure S1**. Operational workflow of the PlasticAnalytics platform.

2.1 Spectral range selection

Spectra acquired from different instruments or using different acquisition settings often cover varying spectral windows. For consistent chemometric analysis, all spectra must first conform to a defined analytical range. Therefore, the data were initially standardised by cropping to target spectral ranges of 4000–400 cm^{-1} for FTIR datasets and 200–3200 cm^{-1} for Raman datasets.

2.2 Spectrum resampling

Within these defined ranges, the original datasets often exhibit varying spectral sampling densities, resulting in an inconsistent number of spectral points (e.g., 500 to 3500). To ensure a standardised data density, the spectra are resampled. This resampling is performed using a linear interpolation function to generate spectra with exactly 1000 evenly spaced wavenumber or Raman shift points across their respective target ranges (Equation 1).

$$f(x) = y_i + \frac{(x - x_i)(y_{i+1} - y_i)}{(x_{i+1} - x_i)}$$

Equation 1

Where $f(x)$ refers to the interpolated intensity value estimated for that specific wavelength value x; x is a specific wavelength value within the target interpolation wavelength range; x_i is the nearest wavelength from the original data less than x; x_{i+1} is the next wavelength value in the original data greater than x_i; y_i is the intensity corresponding to x_i; and y_{i+1} is the intensity corresponding to x_{i+1}.

2.3 Baseline correction – iterative baseline correction

Baseline correction removes the underlying fluorescence or background signal from spectral data, allowing Raman or FTIR bands to be resolved without interference from broad background contributions. Because FTIR spectra acquired in transmittance mode (and ATR spectra when displayed in transmittance-like form) present absorption bands as downward-going troughs, spectra were intensity-inverted so that bands are represented as positive peaks prior to baseline correction preprocessing. Baseline correction algorithms range from simple linear or polynomial models to more advanced methods. Recent developments include the k-iterative double sliding window approach for distinguishing noise from signal and the modified multi-polynomial fitting method, which iteratively models the fluorescence background using polynomial functions.[1–2] To further enhance baseline correction performance on environmental spectra, the iterative asymmetric penalised least squares (i-arPLS) algorithm was developed, integrating adaptive parameter selection for oversaturated regions and a dual-regime correction strategy to accommodate the differing behaviours of saturated and non-saturated spectral domains. This algorithm iteratively calculates the baseline correction value over a maximum of 50 iterations. During each iteration, the diagonal weight matrix (W) is updated based on the intensity values, refining the correction until the optimal parameters are identified (Equation 2).

$$z = (W + \lambda D^T D)^{-1} W y$$

Equation 2

Where W denotes the diagonal weight matrix based on spectrum intensities; D refers to the difference matrix; H outlines the roughness penalty matrix; λ the smoothing parameter; y the

original spectrum; and z being the fitted baseline. The output of Equation 2 is a smoothed baseline fit z that minimises:

$$\sum_i w_i(y_i - z_i)^2 + \lambda \sum_i (d_i z)^2$$

Equation 3

Equation 2 fits z to y based on weights W. Equation 3 penalises, the roughness of z, fitted baseline. From this calculation the diagonal weight matrix (W) values are updated each iteration based on how far the fitted baseline (z) is from spectrum (y):

$$w_i = \frac{1}{1 + e^{2(d_i - (2s - m))/s}}$$

Equation 4

Where m and s depend on distribution of residuals (y-z). This reduces outlier weight, and the iterative process stops when the weights converge below the tolerance values or until the iteration number is reached. For spectra with oversaturated regions, we apply a two-step approach with different parameters: first, for oversaturated regions: $\lambda = 1.5 \times 10^2$ with convergence ratio = 1×10^{-5}, and second, for normal regions: $\lambda = 1 \times 10^5$ with convergence ratio = 1×10^{-4}. To produce the baseline-corrected spectrum the fitted baseline (z) is subtracted from the spectrum (y). This baseline corrected spectrum is then further processed to remove cosmic rays or ambient CO_2 interference.

2.4 Cosmic Ray Removal

Cosmic ray identification combines rule-based mathematical detection with an optional ML enhancement stage, followed by interpolation-based removal.

2.4.1 Validation Methodology

The pipeline was validated using 104 Raman spectra of polymeric reference materials acquired at 532 nm and 785 nm (Table S1), containing both naturally occurring and synthetically added cosmic ray artefacts.

2.4.1.1 Synthetic Cosmic Ray Generation

Synthetic cosmic rays were modelled as Gaussian peaks (Equation 5). Peak positions were randomly distributed across the spectral range, excluding the outer 50 data points at each edge. FWHM values were uniformly sampled between 0.3–1.2 cm^{-1}, and amplitudes were set to 5–20× the local baseline intensity, constrained between 10% and 300% of the spectrum maximum. Pre-normalised spectra (max intensity ≤ 2.0) were temporarily rescaled prior to injection, then all spectra were renormalised to 0–1.

$$I_{\text{cosmic}}(\lambda) = A \cdot \exp\left(-\frac{(\lambda - \lambda_0)^2}{2\sigma^2}\right)$$

Equation 5

where A is the amplitude, λ_0 is the peak position, and σ is the width parameter derived from FWHM via σ = FWHM/2.355. Ray positions (λ_0) were randomly distributed throughout the

spectral range, excluding the outer 50 data points at each edge to ensure complete spectral coverage.

2.4.1.2 Validation Dataset

Cosmic ray contamination was injected into 104 reference Raman spectra. For each spectrum, a fixed number of synthetic cosmic rays was added, beginning at 8 rays for the first spectrum and increasing by 1 ray for each subsequent spectrum, up to a maximum of 20 rays per spectrum. Spectra beyond the 13th continued to receive 20 rays each. This protocol yielded a total of 2,002 injected cosmic rays across the full validation set. All contaminated spectra were intensity-normalised to 0–1 prior to evaluation.

2.4.1.3 Algorithmic Performance metrics

Algorithm performance was quantified using four metrics: detection rate, defined as the proportion of synthetic cosmic rays correctly identified (Equation 6); precision, the fraction of detected features corresponding to true cosmic rays (Equation 7); removal efficiency, the reduction in spectral contamination following correction expressed as an RMS ratio (Equation 8); and Raman peak preservation, quantifying the retention of true Raman intensities after removal (Equation 9).

$$\text{Detection Rate} = \frac{\text{True Positives}}{\text{Total Cosmic Rays Added}} \times 100\%$$

Equation 6

$$\text{Precision} = \frac{\text{True Positives}}{\text{True Positives} + \text{False Positives}}$$

Equation 7

$$\text{Removal Efficiency} = \left(1 - \frac{\text{RMS}_{\text{residual}}}{\text{RMS}_{\text{contamination}}}\right) \times 100\%$$

Equation 8

Where $\text{RMS}_{\text{contamination}}$ is described as: $\text{RMS}_{\text{contamination}} = \sqrt{\frac{1}{N}\sum_{i=1}^{N}(I_{\text{contaminated},i} - I_{\text{original},i})^2}$, and $\text{RMS}_{\text{residual}}$ is described as: $\text{RMS}_{\text{residual}} = \sqrt{\frac{1}{N}\sum_{i=1}^{N}(I_{\text{cleaned},i} - I_{\text{original},i})^2}$.

$$\text{Preservation Ratio} = \frac{I_{\text{cleaned}}}{I_{\text{original}}}$$

Equation 9

A cosmic ray was considered correctly detected when a peak was identified within ±3 Raman shifts of the known insertion position.

Table S1. Summary of reference Raman spectra used for Cosmic Ray removal validation.

Polymer Composition	Excitation Wavelength (nm)	Number of Spectra
**Acrylic**	532	1
**Acrylonitrile butadiene styrene**	785	4
**Cellulose acetate butyrate**	532	1
**Cellulose**	532	2
	785	2
**Cotton**	532	3
	785	5
**High-Density polyethylene**	532	2
**Polyethylene-co-acrylic acid**	532	2
**Polyacetal**	532	1
**Polyamide**	532	2
	785	10
**Polybutylene terephthalate**	532	1
**Polycarbonate**	532	3
**Polyester**	785	8
**Polyethylene terephthalate**	532	3
	785	5
**Polyethylene-vinyl acetate**	532	2
**Polyethylene**	532	3
	785	7
**Polymethyl methacrylate**	785	2
**Polypropylene**	532	2
	785	12
**Polystyrene**	532	3

**Polysulfone**	785	1

Table S1. *Continued.*

Polymer Composition	Excitation Wavelength (nm)	Number of Spectra
**Polyurethane**	532	3
	785	11
**Polyvinyl chloride**	532	3

2.4.2 Rule-Based Cosmic Ray Identification

Candidate peaks are identified by height and prominence thresholds (Equation 10), with Raman band regions protected via spectral smoothing to suppress false positives (Equation 11). Cosmic ray candidates are then identified through three complementary criteria applied in union: FWHM-based detection (Equation 12), which flags peaks below a 1.5 cm^{-1} threshold with an adjustment factor of 0.5 for peaks on Raman bands and 1.0 otherwise; derivative-based detection (Equation 13), which flags peaks based on first and second derivative thresholds adjusted for band presence; and statistical outlier detection (Equation 14), which flags peaks exceeding a z-score threshold of 10 in standard regions and 15 within Raman band regions. The union of these candidate sets is subsequently passed through a final validation function applying stricter criteria for peaks coinciding with Raman bands (Equation 15).

$$P = \{i \mid I(\lambda_i) > I(\lambda_{i-1}) \wedge I(\lambda_i) > I(\lambda_{i+1}) \wedge h(I(\lambda_i)) > h_{\min} \wedge p(I(\lambda_i)) > p_{\min}\}$$

Equation 10

Where P is a set of indices corresponding to peaks in the spectrum; $I(\lambda)$ is intensity as a function of wavelength; $h(I(\lambda i))$: height of peak at position *i*; $p(I(\lambda i))$ refers to prominence of peak at position *i*; h_{min} is minimum peak height threshold (typically 0.05 × max intensity); and p_{min} corresponds to minimum prominence threshold (typically 0.05 × max intensity).

$$B = \{i \mid i \in P_{\text{smooth}} \wedge \text{FWHM}(i) > W_{\text{band}}\}$$

Equation 11

Where B is a set of indices corresponding to Raman band centers; P_{smooth} refers to peaks identified in the smoothed spectrum; $FWHM(i)$ defined as the FWHM of the peak at position *i*; W_{band} refers to the minimum FWHM threshold for Raman bands (typically 30 cm^{-1}).

$$C_{\text{FWHM}} = \{i \mid i \in P \wedge \text{FWHM}(i) < T_{\text{FWHM}} \times f(i)\}$$

Equation 12

Where C_{FWHM} refers to a set of cosmic ray candidates identified based on their FWHM, T_{FWHM} is threshold defined at 1.5 cm^{-1}; and $f(i)$ is the adjustment factor regarded as 0.5 for peaks on bands, and 1.0 otherwise.

$$C_{\text{deriv}} = \left\{ i \mid i \in P \wedge \max \left| I'(\lambda) \right| > T_{d1} \wedge \max \left| I''(\lambda) \right| > T_{d2} \right\}$$

Equation 13

Where C_{deriv} is the set of cosmic ray candidates identified based on peak derivatives; $I\prime(\lambda), I\prime\prime(\lambda)$ is the first and second order derivatives; and T_{d1}, T_{d2} is the derivative thresholds, adjusted based on band presence.

$$C_{\text{stat}} = \left\{ i \mid i \in P \wedge \frac{\sigma_{\text{surround}}}{I(\lambda_i) - \mu_{\text{surround}}} > T_Z \right\}$$

Equation 14

Where C_{stat} is the set of cosmic ray candidates identified based on their statistical analysis; $\mu_{surround}, \sigma_{surround}$ denotes the mean and standard deviation of surrounding regions; while T_Z refers to the z-score threshold being 10 for standard regions, and 15 for Raman band regions.

$$C = \{ i \mid i \in (C_{\text{FWHM}} \cup C_{\text{deriv}} \cup C_{\text{stat}}) \wedge V(i) = \textbf{true} \}$$

Equation 15

Where C is the final set of cosmic ray indices, and $V(i)$ is the validation function that applies stricter criteria for peaks on Raman bands.

2.4.2.1 Rule-Based Threshold Optimisation

To determine the optimal parameters for the mathematical detection criteria, a grid search optimisation was performed across the 104 reference Raman spectra. The objective was to maximise the detection of genuine cosmic rays while minimising the false-positive rate (FPR) on true Raman bands.

The search space evaluated combinations of three primary thresholds: sharpness (1.0 to 6.5), derivative magnitude (0.2 to 1.0), and z-score (5 to 25). Performance was evaluated using precision, recall, and F1-score alongside the mean FPR. The optimal parameter combination was selected based on achieving the lowest mean FPR while maintaining a recall of ≥ 0.80 (Table S2).

2.4.3 Cosmic Ray Removal

Identified cosmic rays are removed by interpolation across the affected spectral region. Three methods are implemented: linear interpolation (Equation 16), median filtering (Equation 17), and PCHIP (Equation 18). PCHIP is the default, as it preserves spectral peak shapes and avoids the oscillations associated with standard cubic spline interpolation. The interpolation method is user-configurable.

Table S2. Grid search space parameters for rule-based cosmic ray detection

Parameters	Search Space
**Sharpness Threshold**	1.0, 1.5, 2.0, 2.5, 3.0, 3.5, 4.0, 4.5, 5.0, 5.5, 6.0, 6.5
**Derivative Threshold**	0.2, 0.3, 0.4, 0.5, 0.6, 0.7, 0.8, 0.9, 1.0
**Z-score Threshold**	5, 7, 10, 12, 15, 17, 20, 22, 25

2.4.3.1 Linear Interpolation

Linear interpolation estimates the corrected intensity between two neighbouring baseline points surrounding the cosmic ray region.

$$I_{\text{interp}}(\lambda) = I(\lambda_L) + \frac{\lambda - \lambda_L}{\lambda_R - \lambda_L}(I(\lambda_R) - I(\lambda_L))$$

Equation 16

Where λ_L and λ_R are the nearest unaffected spectral points on the left and right of the cosmic ray region,and $I(\lambda_L)$and $I(\lambda_R)$ are their corresponding intensities. This approach assumes a linear transition between neighbouring baseline values.

2.4.3.2 Median Interpolation

Median filtering replaces cosmic ray affected points using the median intensity within a local spectral window.

$$I_{interp}(\lambda i) = median(I(\lambda_{i-k}), \ldots, I(\lambda_{i+k}))$$

Equation 17

Where $2k+1$ is the window size centred on spectral point i , and the median operator reduces the influence of sharp spikes caused by cosmic rays.

2.4.3.3 Piecewise Cubic Hermite Interpolating Polynomial Interpolation

PCHIP interpolation constructs a shape-preserving cubic polynomial between baseline points surrounding the cosmic ray region.

$$I_{\text{interp}}(\lambda) = (2t^3 - 3t^2 + 1)\, I_i + (t^3 - 2t^2 + t)\, h\, m_i + (-2t^3 + 3t^2)\, I_{i+1} + (t^3 - t^2)\, h\, m_{i+1}$$

Where

$$t = \frac{\lambda - \lambda_i}{\lambda_{i+1} - \lambda_i}, \quad h = \lambda_{i+1} - \lambda_i$$

Equation 18

Here, $I_i$and I_{i+1} are the baseline intensities at neighbouring grid points, and m_i and m_{i+1} are the corresponding local slopes, computed using monotonicity-preserving constraints. PCHIP interpolation preserves spectral peak shapes and avoids the oscillations that can arise in standard cubic spline interpolation.

2.4.4 Machine Learning Enhancement

A secondary ML detection stage was developed to address challenging cases, particularly broad cosmic rays (FWHM > 2.5 cm^{-1}) that may overlap genuine Raman features and evade mathematical criteria.

2.4.4.1 Training Data

Training data were generated from two sources. First, synthetic cosmic rays (FWHM 0.1–6.5 cm^{-1}) were injected into the 104 normalised reference spectra, with genuine Raman features extracted from the uncontaminated spectra using a height threshold of 1% of maximum intensity and a prominence threshold of 0.5%, yielding a dataset of 3,645 real Raman features (peaks). Second, a synthetic augmentation dataset was generated comprising 2,000 cosmic-ray features spanning four FWHM categories: ultra-sharp (0.1–1.0 cm^{-1}, n=500), sharp (1.0–2.5 cm^{-1}, n=500), mid-range (2.5–4.5 cm^{-1}, n=500), and broad (4.5–6.5 cm^{-1}, n=500), alongside 1,500 synthetic Raman features equally divided between broad low-intensity and sharp high-intensity peaks. Synthetic cosmic ray features were constrained to near-perfect symmetry (0.95–1.05), serving as a key discriminative characteristic relative to the natural asymmetry of genuine Raman peaks (0.7–1.3). Eight peak descriptors were extracted per feature for classifier input: peak height, prominence, FWHM, z-score, height-to-prominence ratio, height-to-FWHM ratio, derivative sharpness, and peak asymmetry.

2.4.4.2 Classifier Training

Random Forest (RF) and Gradient Boosting (GB) classifiers were evaluated. Hyperparameter optimisation used grid search with 5-fold stratified cross-validation, repeated across three independent iterations with different random seeds; final parameters were determined by aggregating results across iterations (Table S3). Weighted F1-score was used as the primary optimisation metric to balance precision and recall under class imbalance. The GB classifier outperformed the RF alternative (achieving a weighted F1-score of 0.9746 ± 0.0018 compared to 0.9737 ± 0.0018) and was subsequently integrated into the analytical pipeline.

2.4.5 Detection Framework

The final pipeline operates in two stages. Stage 1 applies the mathematical detection criteria (Section 2.4.2). Stage 2 applies the GB classifier to peaks not flagged in Stage 1, targeting challenging cases such as broad or overlapping cosmic rays. Both stages incorporate Raman band protection using known polymer band positions from the spectral library to reduce false positives. Key parameters, including FWHM threshold, detection method, and band protection settings, are user-adjustable.

2.5 Ambient CO_2 Identification - FTIR

Spurious absorption features arising from ambient CO_2 were identified in the 2288–2392 cm^{-1} region of FTIR spectra.

Table S3. Hyperparameter search space for Random Forest and Gradient Boosting.

Parameters	Random Forest	Gradient Boosting
**N estimators**	100, 200, 300, 500	50, 100, 200, 300
**Max depth**	10, 15, 20, 25, None	10, 20, 30, None
**Min samples split**	2, 5, 10, 15	2, 5, 10, 20
**Min samples leaf**	1, 2, 4, 8	1, 2, 4
**Max features**	‘Sqrt’, ‘log2’, None, 0.3, 0.5	-
**Class weight**	None, balanced, balanced_subsample	-
**Learning rate**	-	0.01, 0.05, 0.1, 0.2

A fixed buffer zone of 30 cm^{-1} was applied on each side of the target region to define the extended correction window (Equation 19), with baseline sampling points drawn from regions 2.0× the band width beyond each buffer edge (Equation 20).

$$I_{\text{target}} = \{i \mid \lambda_1 \leq \textbf{wavenumbers}[i] \leq \lambda_2\}$$

Equation 19

The buffer zones before and after the CO_2 peak are defined using Equation 20.

$$\lambda_{1,\text{ext}} = \lambda_1 - \Delta\lambda_{\text{buffer}}\lambda_{2,\text{ext}} = \lambda_2 + \Delta\lambda_{\text{buffer}}$$

Equation 20

Where $\Delta\lambda_{\text{buffer}}$ = 30-40 cm^{-1}, depending on the spectral resolution. The spectral data within these buffer zones are then used to interpolate the baseline across the CO_2-affected region, effectively removing the artefact.

2.5.1 Baseline Reconstruction

A monotonic baseline was reconstructed across the CO_2-affected region using PCHIP interpolation (Equation 21), as defined previously in Section 2.4.3.3. Sigmoid weighting functions were applied at the left and right transition boundaries (Equations 22 and 23) to blend the reconstructed baseline smoothly with the original spectrum, and were subsequently smoothed via Savitzky-Golay filtering to ensure continuous derivatives at the boundaries (Equation 24). The corrected intensities were computed as a weighted blend between the original and baseline values (Equation 25), followed by a final Savitzky-Golay smoothing pass with smoothing factor β = 0.98.

$$\lambda_{1,\text{sample}} = \max\left(\lambda_1 - \alpha(\lambda_2 - \lambda_1), \lambda_{\text{next\_peak\_base}}\right)$$
$$\lambda_{2,\text{sample}} = \min\left(\lambda_2 + \alpha(\lambda_2 - \lambda_1), \lambda_{\text{prev\_peak\_base}}\right)$$

Equation 21

Where α = 2.0-2.5 representing the standard extended region factor, and $\lambda_{\text{next\_peak\_base}}$ and $\lambda_{\text{prev\_peak\_base}}$ are the wavenumbers at the base of the nearest peaks before and after the CO_2 region, respectively.

$$w_{\text{left}}(x) = 1 - \frac{1}{1 + e^{-10(t-0.5)}}$$

$$w_{\text{right}}(x) = \frac{1}{1 + e^{-8(t-0.5)}}$$

Equation 22

Where *t* ranges from 0 to 1 across each transition region.

$$w_{\text{smooth}} = \text{SG}(w, \text{window} = 15, \text{order} = 3)$$

Equation 23

$$I_{\text{corrected}}(x) = w_{\text{smooth}}(x) \cdot I_{\text{baseline}}(x) + (1 - w_{\text{smooth}}(x)) \cdot I_{\text{original}}(x)$$

Equation 24

$$I_{\text{final}} = \beta \cdot \text{SG}(I_{\text{corrected}}, \text{window}, \text{order}) + (1 - \beta) \cdot I_{\text{corrected}}$$

Equation 25

Where *β = 0.98-0.99* is a smoothing factor that controls the strength of the final smoothing.

2.6 Feature scaling

Spectra were normalised to the 0–1 intensity range by dividing each intensity value by the spectrum maximum, ensuring a consistent intensity scale for chemometric classification. (Equation 26).

$$y_i' = \frac{y_i}{y_{\max}}$$

Equation 26

Where: y_i' is the normalised intensity value for the ith wavelength; y_i is the original interpolated intensity value from the resampled spectrum; and $y_{\max} = \max(y_i)$the spectral maximum.

2.7 Correlative analysis

The Pearson correlation coefficient (PCC) is used across the PlasticAnalytics chemometric suite. PCC is used to evaluate OOD spectra for quality assurance and quality control (QA/QC) through comparison with a reference database, enabling inclusion or rejection of candidate spectra. It is also applied within a reference-aware spectral clustering framework to support DRN predictions and identify spectra deviating from the training distribution.

2.7.1 Correlative analysis for new-spectral-candidate addition by user

All user-uploaded spectra are subjected to correlative analysis to verify consistency with the user-declared polymer composition prior to database inclusion. This assessment is performed using pairwise PCC analysis across spectra within the corresponding reference class (Equation 27). The average correlation coefficient for each spectrum is then computed (Equation 28), providing a measure of consistency with the reference dataset.

$$r_{ij} = \frac{\sum_{k=1}^{n}(x_{ik} - \bar{x}_i)(x_{jk} - \bar{x}_j)}{\sqrt{\sum_{k=1}^{n}(x_{ik} - \bar{x}_i)^2}\sqrt{\sum_{k=1}^{n}(x_{jk} - \bar{x}_j)^2}}$$

Equation 27

Where: r denotes the correlation coefficient; n being the number of points between start and end wavelength; x_i, y_i = Intensity values at the ith wavelength; and $\bar{x}$, $\bar{y}$ = Mean intensity values between start and end wavelength.

$$\bar{r}_i = \frac{1}{N-1}\sum_{j\neq i} r_{ij}$$

Equation 28

Where n is the total number of spectra. Spectra exhibiting $\bar{\rho}_i > \tau$(correlation threshold $\tau = 0.7$) are classified as high-quality, while those with$\bar{\rho}_i \leq \tau$ are assigned to the low-quality cohort.

2.7.2 Spectral Quality Clustering Framework

A reference-aware clustering framework was developed to categorise spectra by conformity to polymer-specific signatures, enabling robust identification across pristine and weathered microplastics. Spectra are grouped into three tiers: consensus, variant, and complete reference spectra. The consensus reference is the mean of the high-correlation group, representing pristine plastic signatures with minimal variation. The variant reference is the mean of the low-correlation group, capturing variability from additives, degradation, and manufacturing differences. The complete reference is the mean of all spectra, providing maximal spectral coverage.

2.7.2.1 Correlation-based Clustering

All polymer spectra in the instrument and polymer-type database were subjected to correlation-based clustering. The number of spectra per polymer class varied depending on availability from in-house measurements, published literature, and open-access spectral databases. For polymer classes with three or fewer spectra, a fixed correlation threshold of 0.99 was applied, such that only spectra with pairwise correlation coefficients >0.99 were assigned to the consensus group. For polymer classes with more than three spectra, the optimal correlation threshold was determined using the elbow method applied to pairwise PCC coefficients. The correlation matrix was computed for all spectral pairs using Equation 29.

$$C_{ij} = PCC(I_i, I_j)$$

Equation 29

Where I_i and I_j represent intensity vectors for spectra *i* and *j*. Following the correlation matrix computation, the average correlation for each spectrum is then calculated using Equation 30.

$$\rho_i = \frac{1}{n-1}\sum_{j\neq i} C_{ij}$$

Equation 30

The elbow point in the distribution of low-correlation spectra as a function of threshold identifies τ_{opt}, which separates consensus (ρ_i > τ_{opt}) from variant (ρ_i ≤ τ_{opt}) groups.

2.7.2.2 Three-Tier Reference Structure

Following clustering, three reference spectra were generated for each polymer class: consensus, variant, and complete reference spectra. These were computed as the mean

spectra of the consensus group, variant group, and full dataset, respectively, using Equation 31.

$$R_g(\nu) = \frac{1}{n_g} \sum_{i=1}^{n_g} I_i(\nu)$$

Equation 31

Where $R_g(v)$ is the mean intensity at wavenumber v for group g, n_g is the number of spectra in group g, and $I_i(v)$ is the intensity of spectrum i at wavenumber v.

2.7.2.3 Divergence Analysis

Spectral divergence between consensus and variant groups was quantified using root mean square deviation (RMSD) and identification of divergent peaks. Regions where the deviation exceeded a defined significance threshold were flagged as indicative of chemical modification. The threshold was determined from the standard deviation of the consensus spectrum intensity.

The resulting reference library, spanning Raman microscopy and FTIR across multiple acquisition modes, was used for out-of-distribution analysis and spectral classification.

2.8 Machine Learning - Deep Residual Network

The classification framework is based on a DRN architecture designed to learn complex patterns from Raman and FTIR spectral data.

2.8.1 Creation of the Plastic Raman and FTIR Spectral Library

The PlasticAnalytics spectral library comprises Raman and FTIR spectra acquired from multiple sources and instrument platforms. This section describes the data sources, instrument types, and acquisition configurations used to compile the dataset.

2.8.1.1 Plastics contained in the PlasticAnalytics Database

Raman and FTIR spectra were compiled from published datasets and open-access spectral libraries.[3–22] A detailed summary of FTIR and Raman data sources is provided in Tables S4 and S5.

Table S4. Source, material classes, sample conditions, and total number of spectra in the Fourier transform infrared (FTIR) spectral library.

**Mode**	Reference / Source	Material Classes	Sample Condition	Total Spectra (n)
ATR	Gómez-Bacab et al. [16]	Polypropylene (CaCO3), Polypropylene (Flame Retardant), Polypropylene (Glass Fiber), Polypropylene (Green), Polypropylene (Red), Polypropylene (White)	100.0% Pristine	791
ATR	Jiang et al. [17]	Polyethylene, Polypropylene	100.0% Pristine	600
ATR	Kedzierski et al. [18]	Cellulose acetate, Ethylene propylene rubber, Morphotype 1, Morphotype 2, Polyethylene, Polypropylene, Polystyrene, Polyvinyl chloride, Animal fibre, Cellulose, Ethylene vinyl acetate, Polyamide, Polymethyl methacrylate, Polyurethane	100.0% Weathered	960
ATR	Lenz et al. [15]	High-density polyethylene, Low-density polyethylene, Polyethylene, Polyethylene terephthalate, Polylactic acid, Polypropylene, Polystyrene	26.8% Weathered / 73.2% Biofouled	3832
ATR	Manap et al. [19]	High-density polyethylene, Polyethylene, Polypropylene, Polypropylene (Isotactic), Protein	100.0% Weathered	5
ATR	Meyers et al. [5]	Polyethylene, Polyethylene terephthalate, Polypropylene, Polystyrene, Polyvinyl chloride, Polyurethane	23.8% Pristine / 76.2% Weathered	21
ATR	Primpke et al. [7]	Cellulose acetate, Polybutylene terephthalate, Polyethylene, Polyethylene terephthalate, Polylactic acid, Polypropylene, Polystyrene, Polyvinyl chloride, Acrylonitrile butadiene, Acrylonitrile butadiene styrene, Algae, Alginic acid, Alkyd varnish, Amber, Animal fibre, Aramid, Butyl-methacrylate isobutyl methacrylate, Cellulose, Cellulose acetate butyrate, Cellulose propionate, Cellulose triacetate, Chitin, Coal, Epoxide, Ethyl-cellulose, Ethylene acrylic acid, Ethylene ethyl acrylate, Ethylene methacrylic acid, Ethylene propylene, Ethylene vinyl acetate, Ethylene vinyl alcohol, Fur, Honeycomb, Hydroxyethyl cellulose, Hydroxypropyl cellulose, Hydroxypropyl methyl cellulose, Lahmian Medium acrylic paint, Methyl-cellulose, Methyl-vinyl ether maleic acid, Methyl-vinyl ether maleic anhydride, N-vinylpyrrolidone vinyl acetate, Phenoxy resin, Plant fibre, Poly-1-butene isotactic, Poly 2-4-6-tribromostyrene, Poly-2-6-dimethyl-p-phenylene oxide, Poly-2-hydroxyethyl-methacrylate, Poly-4-4'-dipropoxy-2-2'-diphenyl propane fumarate, Poly-4-methyl-1-pentene, Poly-n-butyl methacrylate, Poly-p-phenylene ether sulphone, Polyacetal, Polyacrylamide, Polyacrylic acid, Polyamide, Polybutadiene, Polycaprolactone, Polycarbonate, Polychloroprene, Polydiallyl isophthalate, Polyester,	50.0% Pristine or Consumer / 50.0% Weathered	322

		Polyester epoxide, Polyesterurethane, Polyetherester, Polyetheretherketone, Polyetherurethane, Polyethyl methacrylate, Polyethylene glycol, Polyethylene oxide, Polyhydroxybutyric acid, Polyimide, Polyisobutyl methacrylate, Polyisoprene, Polymethyl methacrylate, Polyoxymethylene, Polyphenylene sulfide, Polyphenylsulfone, Polysulfone, Polytetrafluoroethylene, Polyurethane, Polyurethane acrylic resin, Polyvinyl acetate, Polyvinyl alcohol, Polyvinyl butyral, Polyvinyl formal, Polyvinyl stearate, Polyvinylidene fluoride, Polyvinylpyrrolidone, Resin dispersion, Rubber, Silicone, Styrene acrylonitrile, Styrene allyl alcohol, Styrene butadiene, Styrene butyl methacrylate, Styrene ethylene butylene, Styrene isoprene, Styrene maleic anhydride, Styrene maleic anhydride partial methyl ester, Thermoplastic elastomer, Vinyl chloride vinyl acetate, Vinyl chloride vinyl acetate hydroxypropyl acrylate, Vinyl chloride vinyl acetate maleic acid, Vinylidene chloride acrylonitrile, Viscose, Wood, Wood glue, Zein		
ATR	De Frond et al. [20]	Polyethylene	100.0% Weathered	10
ATR	Villegas-Camacho et al. [4]	High-density polyethylene, Low-density polyethylene, Polyethylene terephthalate, Polypropylene, Polystyrene, Polyvinyl chloride	100.0% Pristine	3004
ATR	Authors	Polystyrene, Polyethylene, Low-density Polyethylene, Polyethylene terephthalate	100.0% Pristine	5

Table S5. Source, material classes, sample conditions, and total number of spectra in the Raman Microscopy Spectral Library.

Wavelength	Reference / Source	Material Classes	Sample Condition	Total Spectra (n)
532 nm	Cabernard et al. [21]	1,2-Polybutadiene, Vinyl chloride, Vinyl acetate, Maleic acid, Vinyl chloride–vinyl acetate copolymer (90:10)	100.0% Pristine	5
532 nm	Munno et al. [8] (SLoPP)	Acrylonitrile butadiene styrene, Acrylic, Cotton, Polyamide, Polycarbonate, Polyethylene, Polyethylene Vinyl Acetate, Polyethylene terephthalate, Polypropylene, Polystyrene, Polyurethane, Polyvinyl chloride	100.0% Pristine	69
532 nm	Hogan et al. [22] (RaSPI)	Acrylonitrile butadiene styrene, Polyamide, Polycarbonate, Polyester, Polyethylene, Polyethylene terephthalate, Polymethyl methacrylate, Polyoxymethylene, Polypropylene, Polystyrene, Polytetrafluoroethylene, Polyurethane, Polyvinyl chloride, Silicone	11.0% Weathered / 89.0% Other	172
532 nm	Authors	Acrylonitrile butadiene styrene, Cellulose acetate, Nitrocellulose, Polyacrylonitrile, Polyamide, Polyamide 12, Polyamide 6, Polyamide 6,6, Polybutylene adipate terephthalate, Polybutylene terephthalate, Polycarbonate, Polyethylene, Polyethylene terephthalate, Polyhydroxybutyrate, Polyisoprene rubber, Polylactic acid, Polymethyl methacrylate, Polyoxymethylene, Polypropylene, Polystyrene, Polysulfone, Polytetrafluoroethylene, Polyurethane, Polyurethane [thermoset], Polyvinyl alcohol, Polyvinyl chloride, Polyvinylidene fluoride, Sodium polyacrylate, Styrene butadiene	88.1% Pristine / 11.9% Weathered	1360
633 nm	Hogan et al. [22] (RaSPI)	Acrylonitrile butadiene styrene, Polyamide, Polyester, Polyethylene, Polyethylene terephthalate, Polymethyl methacrylate, Polyoxymethylene, Polypropylene, Polystyrene, Polytetrafluoroethylene, Polyurethane, Polyvinyl chloride, Silicone	8.5% Weathered / 91.5% Other	224
633 nm	Authors	Acrylonitrile butadiene styrene, Polyacrylonitrile, Polyamide, Polyethylene terephthalate, Polypropylene, Polystyrene	100.0% Pristine	121
785 nm	Authors	High-density polyethylene, Low-density polyethylene, Polyamide, Polyethylene, Polyethylene terephthalate, Polymethyl methacrylate, Polypropylene, Polystyrene, Polyvinyl chloride, Tyre rubber	100.0% Pristine	340
785 nm	Cabernard et al. [21]	Acrylonitrile, Acrylonitrile butadiene styrene, Acrylonitrile butadiene styrene-containing-pigment, Alkyd resin, Alkyd varnish, Beeswax, Biaxially Oriented Polypropylene, Cashmere, Cellulose,	100.0% Pristine	204

		Cellulose acetate, Cellulose acetate butyrate, Cellulose propionate, Cellulose triacetate, Chitin, Co-extruded-polyethylene-polyamide-polyethylene, Cotton, Ethyl cellulose, Ethylene acrylic acid, Ethylene-vinyl acetate copolymer, Ethylene-vinyl acetate copolymer 18%, Ethylene-vinyl acetate copolymer 33%, Ethylene-ethyl acrylate copolymer, Ethylene-methacrylic acid copolymer, Ethylene-propylene copolymer, Ethylene-vinyl acetate (14%) copolymer, Ethylene-vinyl acetate (28%) copolymer, Ethylene-vinyl acetate (40%) copolymer, Ethylene-vinyl alcohol (18%), Ethylene-vinyl alcohol copolymer, Expanded Polystyrene, Glycol-modified polyethylene terephthalate, Hammerite red special adhesive primer resin dispersion, High-density polyethylene, High-gloss alkyd resin paint, Hydroxyethyl cellulose, Hydroxypropyl cellulose, Hydroxypropyl methyl cellulose, Lahmian Medium acrylic paint, Lignin-based Biocomposite, Low-density polyethylene, Methyl vinyl ether, Methylcellulose, N-vinylpyrrolidone, Natural Rubber, Natural silk, Paraffin, Phenoxy resin, Poly n-butyl methacrylate, Poly(1-butadiene), isotactic, Poly(2,6-dimethyl-p-phenylene oxide), Poly(2-hydroxyethyl methacrylate), Poly(3-hydroxybutyrate), Poly(4,4'-dipropoxy-2,2'-diphenylpropane fumarate), Poly(4-methyl-1-pentene), Poly(butylene adipate-co-terephthalate), Polyacetal, Polyacrylamide, Polyacrylic acid, Polyamide, Polyamide 11, Polyamide 12, Polyamide 6, Polyamide 6(3)T, Polyamide 6,12, Polyamide 6,6, Polyamide 6,9, Polyamide 66, Polybutylene terephthalate, Polycaprolactone, Polycarbonate, Polydiallyl isophthalate, Polyester, Polyester urethane, Polyester epoxy, Polyethyl methacrylate, Polyethylene, Polyethylene glycol, Polyethylene oxide, Polyethylene terephthalate, Polyethylene-chlorinated, Polyisobutyl methacrylate, Polyisoprene-chlorinated, Polylactic acid, Polylactide, Polymethyl methacrylate, Polyoxymethylene, Poly-p-phenylene ether sulphone, Polyphenylene sulfide, Polyphenylsulfone, Polypropylene, Polystyrene, Polysulfone, Polytetrafluoroethylene, Polyurethane, Polyurethane acrylic, Polyvinyl acetate, Polyvinyl alcohol, Polyvinyl butyral, Polyvinyl chloride, Polyvinyl formal, Polyvinyl stearate, Polyvinylidene fluoride, Polyvinylpyrrolidone, Quartz, Sand, Styrene acrylonitrile, Styrene allyl alcohol, Styrene butadiene, Styrene butyl methacrylate, Styrene maleic anhydride, Styrene-ethylene-butylene-styrene, Styrene-isoprene-styrene, Styrene-maleic anhydride, partial methyl ester, Vinyl chloride, Vinylidene chloride, Viscose, Wool, n-Butyl methacrylate		
785 nm	Munno et al. [8] (SLoPP)	Acrylonitrile butadiene styrene, Acrylic, Cellulose acetate, Cotton, Polyamide, Polycarbonate, Polyester, Polyethylene, Polyethylene terephthalate, Polymethyl methacrylate, Polypropylene, Polystyrene, Polyvinyl chloride	100.0% Pristine	74

2.8.1.2 Dataset augmentation

To improve model generalisation and simulate real-world variability in spectral measurements, dataset augmentation was applied to Raman and FTIR spectra. Augmentation strategies were designed to account for noise, baseline distortions, and class imbalance commonly observed in spectroscopic datasets.

2.8.1.2.1 Noise Addition

Random noise was added to spectral intensities to simulate measurement variability arising from fluorescence, instrumental noise, and sample heterogeneity. Noise was sampled from a normal distribution with zero mean, with magnitude scaled relative to the intensity range of the original spectrum (Equation 32).

$$I_{noise} = I_{original} + \varepsilon$$

Equation 32

Where I_{noise} represents the noise-augmented spectrum; $I_{original}$ represents the original Raman or FTIR spectrum; and ε is random noise generated from a normal distribution with mean 0 and standard deviation σ_{noise}. The noise magnitude σ_{noise} is proportional to the original spectrum's intensity range and the specified noise level parameter, calculated as σ_{noise} = (I_{max} - I_{min}) × noise_level × 0.1.

2.8.1.2.2 Baseline transformation

Baseline distortions were introduced to replicate common spectral artefacts arising from scattering effects, surface contamination, and chemical heterogeneity. A range of baseline profiles was applied, including linear, exponential, quadratic, sigmoidal, and Gaussian functions. Baseline parameters were randomly sampled to generate diverse spectral variants representative of real-world conditions.

2.8.1.2.2.1 Linear Baseline Profile

The linear baseline transformation was calculated using Equation 33.

$$B_{linear}(x) = m \times x$$

Equation 33

where $B_{linear}(x)$ represents the linear baseline contribution at position x; m is the slope coefficient determined as $m = \pm(amplitude/length)$, with amplitude being proportional to the spectrum's intensity range and the specified noise level; and the sign (±) is randomly selected to create either positive or negative slopes.

2.8.1.2.2.2 Exponential Baseline Profile

Equation 34 outlines the calculation of baseline values representing an exponential profile.

$$B_{\exp}(x) = \left\{ \text{amplitude} \times \frac{e^{rx}-1}{e^{r\cdot\text{length}}-1} \quad \text{for growth amplitude} \times \frac{e^{r(\text{length}-x)}-1}{e^{r\cdot\text{length}}-1} \quad \text{for decay} \right.$$

Equation 34

where $B_{\exp}(x)$ represents the exponential baseline contribution at position x; r is the rate parameter randomly selected from a uniform distribution between $1/length$ and $5/length$; and the direction (growth or decay) is randomly chosen to simulate both increasing and decreasing exponential trends.

2.8.1.2.2.3 Quadratic Baseline Profile

The quadratic baseline calculation simulates parabolic distortion patterns commonly observed in spectroscopic data (Equation 35).

$$B_{quad}(x) = a \cdot (x - c)^2$$

Equation 35

Where $B_{quad}(x)$ represents the quadratic baseline contribution at position x; a is a coefficient determining both magnitude and direction of the parabola, calculated as $a = \pm amplitude/length2$ with the sign randomly chosen; c represents the center point (vertex position) randomly selected along the spectrum's domain; and x represents the position along the x-axis.

2.8.1.2.2.4 Sigmoid Baseline Profile

Sigmoidal baseline profile generation was conducted using Equation 36.

$$B_{\text{sig}}(x) = d \cdot \frac{\text{amplitude}}{1 + e^{-(x-m)\cdot s}}$$

Equation 36

Where e is a constant (approximately 2.71828) serving as the base of the natural logarithm; x represents the position along the x-axis; m is the midpoint parameter randomly selected between 30-70% of the spectrum's length, determining where the sigmoid function transitions between its plateaus; s is the steepness parameter randomly selected between 0.01-0.05, controlling the slope of the transition region; and d is the direction parameter (either +1 or -1), determining whether the sigmoid increases or decreases across the spectrum.

2.8.1.2.2.5 Gaussian Baseline Profile

Gaussian baseline profiles were generated using Equation 37 to simulate broad, localised baseline features.

$$B_{gaussian}(x) = A \cdot e^{-\left(\frac{x - x_c}{w}\right)^2}$$

Equation 37

Where x_c is the center position randomly selected between 30-70% of the spectrum's length, and w is the width parameter, randomly selected between $L/10$ and $L/5$.

2.8.1.2.3 Dataset augmentation strategies

Three augmentation strategies were implemented. In the original augmentation, spectra were modified through random combinations of noise addition and baseline distortion, followed by baseline correction, smoothing, and normalisation. In the i-arPLS augmentation, the same procedure was applied, but with adaptive baseline correction using iterative asymmetric penalised least-squares to better preserve spectral features under varying baseline conditions. In the SMOTE augmentation, synthetic spectra were generated in feature space using the Synthetic Minority Over-sampling Technique (SMOTE), which interpolates between spectra of the same class to maintain class-specific spectral structure.

2.8.1.3 Ablation Study: Determination of Minimum Original Spectra and Augmentation Target

To systematically evaluate the influence of the number of original training spectra and the extent of data augmentation on classification performance, an ablation study was conducted. The experimental design evaluated three independent axes in a full factorial grid: (i) the number of original seed spectra per polymer class (5, 10, 20, 50, 100, 200, and 500), (ii) the target number of spectra per class following augmentation (500, 1,000, 2,000, 4,000, and 8,000), and (iii) the three augmentation strategies described in Section 2.8.1.2.3 (Original pipeline, i-arPLS pipeline, and SMOTE).

Prior to augmentation, the complete spectral dataset was partitioned via stratified sampling into a training/validation set (85%) and a held-out test set (15%). The test set was never exposed to augmentation and was reserved exclusively for final performance evaluation to ensure an unbiased estimate of generalisation. Model optimisation was performed using 3-fold stratified cross-validation on the augmented training/validation partitions. Our optimised DRN (Table S15, Configuration 09) was trained using mixed-precision computation on an NVIDIA A100 GPU. Training hyperparameters comprised a batch size of 512, a learning rate of 4.85×10^{-4} with a cosine annealing scheduler, and a maximum of 30 epochs. Early stopping with a patience of 5 epochs was applied, monitored via validation macro-F1 score and accuracy.

The primary evaluation metrics were macro-F1 score and accuracy computed on the held-out test set. To quantify the structural variance introduced by the different augmentation strategies, and thereby assess whether augmented spectra constituted meaningfully diverse training examples rather than near-duplicates, the spectral diversity of each augmented dataset was measured as the mean pairwise Pearson correlation coefficient ($\bar{r}$) across randomly sampled spectral pairs within each class. Lower $\bar{r}$ values indicate greater chemical diversity and reduced redundancy.

2.8.2 Comparative model architecture evaluation

Machine learning for vibrational spectroscopy prediction is a diverse field with multiple potential approaches.[23] The following model architectures were evaluated for plastic spectral classification: Standard convolutional neural network (CNN), Long Short-Term Memory (LSTM) networks, Transformer, DRN, and Inception CNN (ICNN; Table S6).

Table S6. Evaluated model architectures for spectral classification.

Model Architecture	Description	Training Time (m)	Parameters (Millions)
Standard CNN	5-layer CNN with increasing filter sizes	4.30 ± 0.0	4.20
Long Short-Term Memory Networks (LSTM)	Bidirectional LSTM with attention mechanism	11.7 ± 0.1	0.53
Transformer	4-head attention with 3 encoder blocks	33.0 ± 0.1	0.15
Inception CNN	Multi-scale feature extraction	2.80 ± 0.1	0.04
Deep Residual Learning Network	ResNet with 9 residual modules	4.3 ± 0.0	2.16

2.8.2.1 Model Selection and Hyperparameter Optimisation

The DRN was selected for hyperparameter optimisation. Raman and FTIR datasets were divided into stratified 5-fold cross-validation splits to preserve class distributions. For each hyperparameter configuration, models were trained on four folds and validated on the remaining fold, iterating across all five fold combinations. Architectural hyperparameters were explored via a random search of 30 configurations sampled from a literature-informed hyperparameter space (Table S7). Models were trained using the Adam optimiser with ReduceLROnPlateau learning rate scheduling and L2 regularisation (weight decay: 1×10^{-4}).

Model selection was based on mean cross-validation accuracy and low cross-fold variance. Statistical comparisons between top-performing configurations were conducted using non-parametric tests, with effect sizes quantified to distinguish practical from statistical significance. Final models with optimised hyperparameters were trained using stratified 10-fold cross-validation, and the resulting models were combined into an ensemble by averaging softmax probabilities. Ensemble performance was evaluated on both in-distribution (ID) and out-of-distribution Raman and FTIR spectra.

2.8.3 Handling of model uncertainty: Out-of-distribution detection

To address model overconfidence on spectra outside the training distribution, we implemented an uncertainty-aware OOD detection framework. The framework combines correlation against a reference library, an RF ensemble classifier based on DRN prediction metrics, and a PE-confounder layer. To evaluate performance, OOD detection was performed on 104 FTIR and 104 Raman spectra. The OOD subset comprised 32 Raman spectra and 52 FTIR spectra, with the remaining samples used as ID data.

Table S7. Architectural and training hyperparameter search space

Model hyperparameter	Value	References
**Initial Channel Size**	32, 64, 128	Zhang et al.[24]; Ren et al.[25]
**Kernel size**	3, 5, 7, 9, 11, 13	Lei et al.[26]; Huang et al.[27]
**Residual block number**	8 to 19	Zhang et al.[24]; Ren et al.[25]; Huang et al.[27]
**Channel progressions**	[16, 32, 64], [16, 64, 128], [32, 64, 128], [32, 128, 256], or [64, 128, 256]	Zhang et al.[24]; Ren et al.[25]
**Pooling configuration**	2, 4, 8	Huang et al.[27]
**Squeeze-and-Excitation reduction ratios**	8, 16, 32	Huang et al.[27]
**Fully Connected Layer**	[256], [512], [256, 128], [512, 256], [1024, 512], [512, 256, 128], [1024, 512, 256]	He et al. [28]; Yang et al.[29]
**Learning rates**	10^{-5} to 5×10^{-3}	Lei et al.[26]; Zhang et al.[24]; Ren et al.[25]; Huang et al.[27]
**Dropout rates**	0.1 to 0.5	Lei et al.[26]; Huang et al.[27];
**Epoch number**	15 to 40	Zhang et al.[24]; Ren et al.[25]; Huang et al.[27];
**Early stopping patience**	3 to 7	Zhang et al.[24]; Ren et al.[25];
**Batch size**	256, 512, 1024	Zhang et al.[24]; Huang et al.[27]; Lei et al.[26];

2.8.3.1 Correlative Reference Analysis

The first layer of the OOD framework screened incoming spectra against an instrument-specific reference library using PCC. Spectra falling below a correlation threshold of r = 0.70 were immediately flagged for rejection (PCC), except for Raman spectra from PVC, where a lower threshold of r = 0.55 was applied due to PVC's broad Raman features reducing correlation values.

To mitigate false positives from naturally occurring or anthropogenic non-plastic particles, the reference library incorporates spectra across multiple material classes, including minerals, organics, pigments, chemicals, and polymers, allowing the model to discriminate environmental particulates.[30–33]

2.8.3.2 Random Forest classifier for OOD detection

Uncertainty-aware features used in RF training were engineered from ensemble predictions and penultimate layer embeddings, including measures of predictive diversity, confidence, embedding distance, and spectral similarity. An optimal subset of these features was selected via 3-fold cross-validation and used to train an RF classifier, standardised with z-scores. Hyperparameters for the classifier were optimised via 3-fold grid search, with class weighting applied to account for class frequency differences (Table S8). The trained model produced a

three-tier decision framework (Accept, Verify, Reject) based on dual probability thresholds optimised to balance automation and misclassification rates.

Table S8. Hyperparameter search space for the Random Forest classifier grid search.

Model Parameters	Value
Number of trees	50, 100, 150
Maximum depth of the tree	5, 7, 10
Minimum number of samples required to split an internal node	4, 8, 12
Minimum number of samples required to be at a leaf node	2, 4, 6
Number of features to consider when looking for the best split	Sqrt, log2

The RF model outputs produce a three-tier decision framework (Accept, Verify, Reject) that was established by optimising dual probability thresholds (τ_{low}, τ_{high}) on a held-out validation set. The thresholds were determined using a grid search that maximised a composite scoring function designed to balance the automation rate against classification errors.

2.8.3.3 Deep learning discrimination of polyethylene

The final stage of the OOD framework addressed spectral overlap between polyethylene (PE) and chemically similar compounds, including fatty acids (oleic and stearic acid) and slip additives, which share a $–CH_2–$ aliphatic backbone and can produce false-positive PE assignments.[34–35] Spectra predicted as PE by the DRN and accepted by the preceding two OOD layers were passed to a dedicated disambiguation classifier.

2.8.3.3.1 Hyperparameter Optimisation

2.8.3.3.1.1 Training data selection and separation strategy

Three architectures were evaluated: Lim et al. [35]'s published U-Net, an enhanced U-Net with residual blocks, squeeze-and-excitation attention, and multi-scale feature extraction (Advanced U-Net), and the proposed DRN. The training dataset comprised 2,000 Raman spectra per class of Sodium Dodecyl Sulphate (SDS), PE, PP, stearic acid, oleic acid, and nine stearic–oleic mixture classes (10% increments). For hyperparameter optimisation, 1,000 spectra were selected for training, maximising spectral diversity based on Euclidean distance from class means; the remaining spectra were reserved as a held-out test set.

2.8.3.3.2 Model hyperparameter search space

Hyperparameters for the DRN and Advanced U-Net were optimised via random search over 120 configurations (Table S9) using 5-fold stratified cross-validation, with macro F1-score as the selection criterion.

Table S9. Hyperparameter search space for the DRN and Advanced U-Net Model Optimisation

Model Parameters	Value
Initial channels	16, 32, 64
Kernel sizes	3, 5, 7, 9, 11, [3, 5, 7], [5, 7, 9], [7, 9, 11]
Number of residual blocks	6 to 15
Channel progression templates	[16, 32, 64], [32, 64, 128], [64, 128, 256]
Pool size	2, 4, 8
Squeeze-Excitation reduction	8, 16, 32
Learning rate	10^{-5}, 5 * 10^{-5}, 10^{-4}, 5 * 10^{-4}, 10^{-3}, 5 *10^{-3}
Dropout rate	0.1, 0.15, 0.2, 0.25, 0.3, 0.35, 0.4, 0.45, 0.5
Max epochs	15 to 40
Patience	3 to 7
Batch size	32, 64, 128

Lim et al. [35]'s U-Net was fixed and trained with default parameters (learning rate = 10^{-3}, maximum epochs = 30, early stopping patience = 5). Optimisation included architectural parameters (filter count, kernel size, dropout rate, SE reduction ratio) and training parameters (batch size, learning rate, maximum epochs). Training used the Adam optimiser with weight decay (10^{-4}), mixed-precision FP16, and ReduceLROnPlateau learning rate scheduling; early stopping halted training when macro F1-score did not improve over the specified patience.

2.8.3.3.3 Ablation Study

A subsequent ablation study evaluated the impact of training seed size (20–1,000 spectra per class) and data augmentation targets (1,000–8,000 spectra per class) on generalisation performance. Synthetic spectra were generated by sequentially applying additive Gaussian noise (5–50% amplitude), stochastic baseline distortion, asymmetric least-squares baseline correction, Savitzky–Golay smoothing, and maximum normalisation. Stratified 5-fold cross-validation was used for all ablation experiments, retaining the best model per fold based on macro F1-score and evaluating performance on the held-out test set.

2.8.3.3.4 Evaluation metrics

Evaluation metrics included test accuracy, macro F1-score, per-class F1-scores, and confusion matrices. The ablation results identified the minimum number of seed spectra required for effective augmentation and the optimal augmentation target for each architecture. The best-performing architecture was incorporated into the PlasticAnalytics pipeline as the

final disambiguation layer, which outputs “Accept” or “Reject” for PE predictions passing the preceding OOD stages, with final adjudication performed by the user.

2.8.3.4 Framework Evaluation

Final performance was evaluated on a completely held-out test dataset (n = 104 for FTIR and n = 104 for Raman) by comparing three approaches: (i) the uncertainty-aware RF classifier alone; (ii) PCC screening alone; and (iii) the integrated hierarchical framework, which combines PCC screening and uncertainty-aware RF classification with an additional Raman-specific PE disambiguation step. Performance was assessed using area under the receiver operating characteristic curve (ROC-AUC), precision-recall, and overall classification accuracy, and decision boundaries were visualised by projecting the feature space onto its first two principal components.

2.8.4 Prediction classification comparison: Univariate versus Multivariate

Model performance was evaluated by comparing our ensemble DRN against six established CNN architectures commonly used for microplastic spectral classification. These included the ResNet-Inception and CoordConv-Inception models from Neo et al. [36] for Raman and FTIR spectra, respectively, incorporating skip connections and coordinate-aware convolutions; the CPL Compound Mixtures and CPL Compound Class models from Cooman et al. [37], employing fully connected layers with 10% dropout; a baseline 1D-CNN described by Qin et al. [38] with five convolutional blocks (kernel size = 3, stride = 2) and 0.2 dropout; and the SE + Improved ResNet18 architecture from Huang et al. [27], which introduces modified residual blocks, reordered batch normalisation, activation, and convolution layers, along with SE blocks for channel-wise feature weighting. Further architectural details, training parameters, and implementation environments are provided in the Supporting Information. Predictions from our PCC algorithm using internal Raman and FTIR libraries were also included for comparison.

Models were evaluated on an unseen test set comprising 1,000 FTIR and 276 Raman spectra. After filtering through the two-stage OOD framework, only spectra judged as in-distribution were retained, yielding 953 FTIR and 129 Raman spectra. Ground truth labels were manually validated. Performance was assessed using three primary metrics: composition accuracy (1.0 for exact match, 0 otherwise), chemical family accuracy (0.5 for chemically related polymers, e.g., EVOH scored as EVA), and a rank-weighted score for PCC predictions using an exponentially decaying rank-based scheme. Robustness and efficiency were further assessed by benchmarking the DRN against Bio-Rad KnowItAll software on an external Raman dataset,[9] and by comparing mean inference times (ms) between DRN and PCC. Statistical significance of performance differences was tested using McNemar’s test.

2.9 Spectral Imaging

2.9.1 Substrate Spectra Identification and Removal

2.9.1.1 Dataset Training Approach and Substrate Annotation

Substrate spectra were identified using a combination of manual interrogation, unsupervised clustering, and supervised machine learning to create substrate identification models. Models were trained on plastic-only and environmental spectral imaging datasets (n = 17 FTIR and n = 22 Raman images). Manual background annotations were performed across these images

to provide initial spectral seeds, achieving annotation coverage of 22.50% (FTIR) and 34.35% (Raman) of total image pixels.

Interactive annotation included two modes: brush mode, which expanded local regions within a 4-pixel circular radius based on cosine similarity thresholds relative to the seed spectrum, and flood-fill mode, which used connected region expansion with adaptive similarity thresholds limited to 500 pixels per operation. Thresholds were calculated as the 99.3rd percentile of cosine similarities within regions with peak intensity <30th percentile and spectral variance <40th percentile, yielding cosine similarity thresholds of ≥0.985 for brush mode and 0.995–0.999 for flood-fill mode.

To propagate sparse manual annotations across entire images, automated expansion combined spatial and spectral features. Each image underwent PCA for dimensionality reduction, followed by K-means clustering, with the optimal cluster number determined via silhouette score and elbow method. Clusters were evaluated for peak prominence, peak sharpness, maximum intensity, and known peak contamination score (Levermore et al., [39]), and a composite background score was computed, where low scores indicate higher likelihood of substrate spectra.

2.9.1.2 Random Forest Substrate Detection Models

RF classifiers were trained on substrate spectra, with hyperparameters optimised via grid search informed by literature on Raman spectrum prediction (Table S10).[40–43] Stratified k-fold cross-validation ensured proportional representation of substrate classes across all folds.

Models combined RF probabilities (weight = 0.6) with PCA K-means clustering consensus (weight = 0.4), assigning substrate labels only if ensemble confidence ≥ 0.7. Spatial coordinates of identified substrate spectra were used to generate binary masks for background removal.

The optimal configuration was selected based on mean cross-validation accuracy, prioritising high detection performance (>99.5%) and low fold variance ($\sigma < 0.001$). Secondary criteria included training time (<3000 s) and balanced precision, recall, and F1-score across all classes. Generalisation was assessed via nested stratified cross-validation (5- and 10-fold), with the outer loop evaluating final performance and the inner loop performing hyperparameter selection. Statistical significance was determined from 95% confidence intervals, and a coefficient of variation <0.1% indicated high model stability.

Table S10. Hyperparameter variables for Random Forest training grid-search optimisation

Parameters	Value	References
Number of trees	50, 100, 200, 300	Zhang et al. [41], Seifert [42], Mayorova et al. [40]
Maximum depth of the tree	10, 20, 30, None	Zhang et al. [41], Mayorova et al. [40]
Minimum number of samples required to split an internal node	2, 5, 10, 20	Chen et al. [43]
Minimum number of samples required to be at a leaf node	1, 2, 4	Zhang et al. [41]
Number of features to consider when looking for the best split	‘sqrt’, ‘log2’	Zhang et al. [41], Seifert [42]
Whether bootstrap samples are used when building trees	‘True’, ‘False’	Zhang et al. [41], Mayorova et al. [40]

2.9.1.3 Validation and cross-dataset performance

The pre-trained semi-supervised ensemble model, combining Random Forest and PCA K-means clustering, was applied to 25 Raman and 53 FTIR spectral images to identify, mask, and remove substrate spectra and pixels. Substrate labels were assigned using a weighted ensemble score (0.6 RF, 0.4 K-means) with a confidence threshold of ≥0.7. Datasets were stratified by image size to account for fixed input-output operations and initialisation overhead, enabling representative assessment of high-throughput performance. The downstream impact of substrate-masking preprocessing on computational efficiency was evaluated by comparing subsequent PCC and deep learning analyses on reduced datasets.

2.9.1.4 Spectral Image Dimension Calculation

The spatial dimensions (X and Y) of each spectral image were automatically determined from the dataset. When explicit coordinates were available, the image height and width were inferred by counting the unique X and Y values. If coordinates were absent, dimensions were estimated from the total number of spectra [N] using Equation 38, selecting the factor pair that approximates a square layout (Equation 39). For visualisation and downstream analysis, 1D spectral data were mapped consistently to a 2D grid based on sorted coordinates. GPU acceleration was applied to the PCC algorithm to improve computational efficiency prior to evaluating background-subtraction effects on pipeline throughput.

$$H \times W = N \quad \text{with} \quad \min|H - W|$$

Equation 38

Subject to:

$$H, W \in \mathbb{Z}^{+} \quad \text{and} \quad N \mod H = 0$$

Equation 39

The factor pair (H, W) satisfying these conditions and yielding the smallest absolute difference $\mid H - W \mid$ was selected to approximate a square-shaped image layout.

2.9.1.5 Plastic scope and Microplastic Metadata

After substrate removal, sample spectra were preprocessed using weighted background subtraction with iterative i-arPLS, Savitzky–Golay filtering, and removal of cosmic-ray or CO_2 signals. Preprocessed spectra were analysed via Microplastic Scope, spatially reconstructed, and examined for microplastics, with morphological properties including size and shape reported for each particle.

2.9.1.6 Microplastic Shape and Size

Microplastic regions identified by PCC or DRN were assigned polymer types, and their morphology was quantified using length (L) and width (W) measurements. Morphological classification was determined from the length:width ratio: fibres (>3:1), spheres (0.85–1), and particles (all other shapes). Length and width were calculated from the positive signal regions using Equations 40 and 41.

$$F_{max} = \max_{i,j} \sqrt{(x_i - x_j)^2 + (y_i - y_j)^2}$$

Equation 40.

$$F_{min} = \min_{\theta} \left[\max_{i}(x_i \cos\theta + y_i \sin\theta) - \min_{j}(x_j \cos\theta + y_j \sin\theta) \right]$$

Equation 41.

Where: (xi, yi) and (xj, yj) are coordinates of boundary points of the particle; θ represents the rotation angle; $Fmax$ is the maximum distance between any two points on the particle boundary; and $Fmin$ is the minimum width of the particle when rotated through all angles. Positive thresholds for PCC and ML analysed spectral images differ. Positive microplastic signals were defined as spectra with PCC to intra-plastic linearity (r>0.78) and ML model confidence scores above the OOD-filtered threshold.

2.9.2 Image Inspector

The Image Inspector provides a click-based interface for rapid interrogation of spectral images. Upon uploading, it generates projections of maximum intensity, integrated intensity, spectral variance, peak position, and correlation with stored FTIR or Raman reference spectra. Users can select any pixel to view its corresponding spectrum in the spectral plot.

2.9.2.1 Maximum intensity

The Maximum Intensity Projection (MIP) identifies the highest spectral intensity within a user-defined wavelength/wavenumber range [$\lambda_{start}, \lambda_{end}$] for each pixel. An interactive spectral slider allows dynamic selection of the spectral window. The resulting MIP image (I_{MIP}) is normalised to the global maximum intensity to ensure consistent visual contrast across datasets (Equation 42–43).

$$I_{MIP}(x,y) = \max_{\lambda \in [\lambda_{start}, \lambda_{end}]} \{S(x,y,\lambda)\}$$

Equation 42.

$$I'_{MIP}(x,y) = \frac{I_{MIP}(x,y)}{\max(I_{MIP})}$$

Equation 43.

2.9.2.2 Integrated intensity

The Integrated Intensity Projection (IIP) computes the total spectral area within a selected spectral window for each pixel. The integrated intensity ($I_{Integrated}(x,y)$) is calculated as the sum of all intensities across the chosen range (Equation 44).

$$I_{\text{Integrated}}(x,y) = \sum_{\lambda=\lambda_1}^{\lambda_2} S(x,y,\lambda)$$

Equation 44.

2.9.2.3 Correlation Map

The Correlation Map Projection (CMP) visualises the spatial distribution of chemical components by comparing each pixel spectrum to reference FTIR or Raman spectra using the PCC algorithm. Spectra are preprocessed (e.g., background removal, baseline correction) to improve signal quality and computational efficiency. For each reference spectrum, a correlation map is generated where pixel intensity represents the PCC value between the unknown and reference spectra.

2.10 Reference library architecture

Each polymer class contains two reference entries: one for virgin/high-quality spectra and one for environmentally degraded or chemically modified spectra. This structure captures spectral variability in aged or post-consumer plastics while preserving accurate identification of virgin material. If fewer than two spectra exist for a class, a conventional per-class average is used. These quality-stratified, instrument- and mode-specific references are then used for PCC-based univariate classification.

3. Results

3.1 Plastic types currently available

The 16 ATR-FTIR plastic classes included in our library and predicted by the DRN are shown in Figure S2.

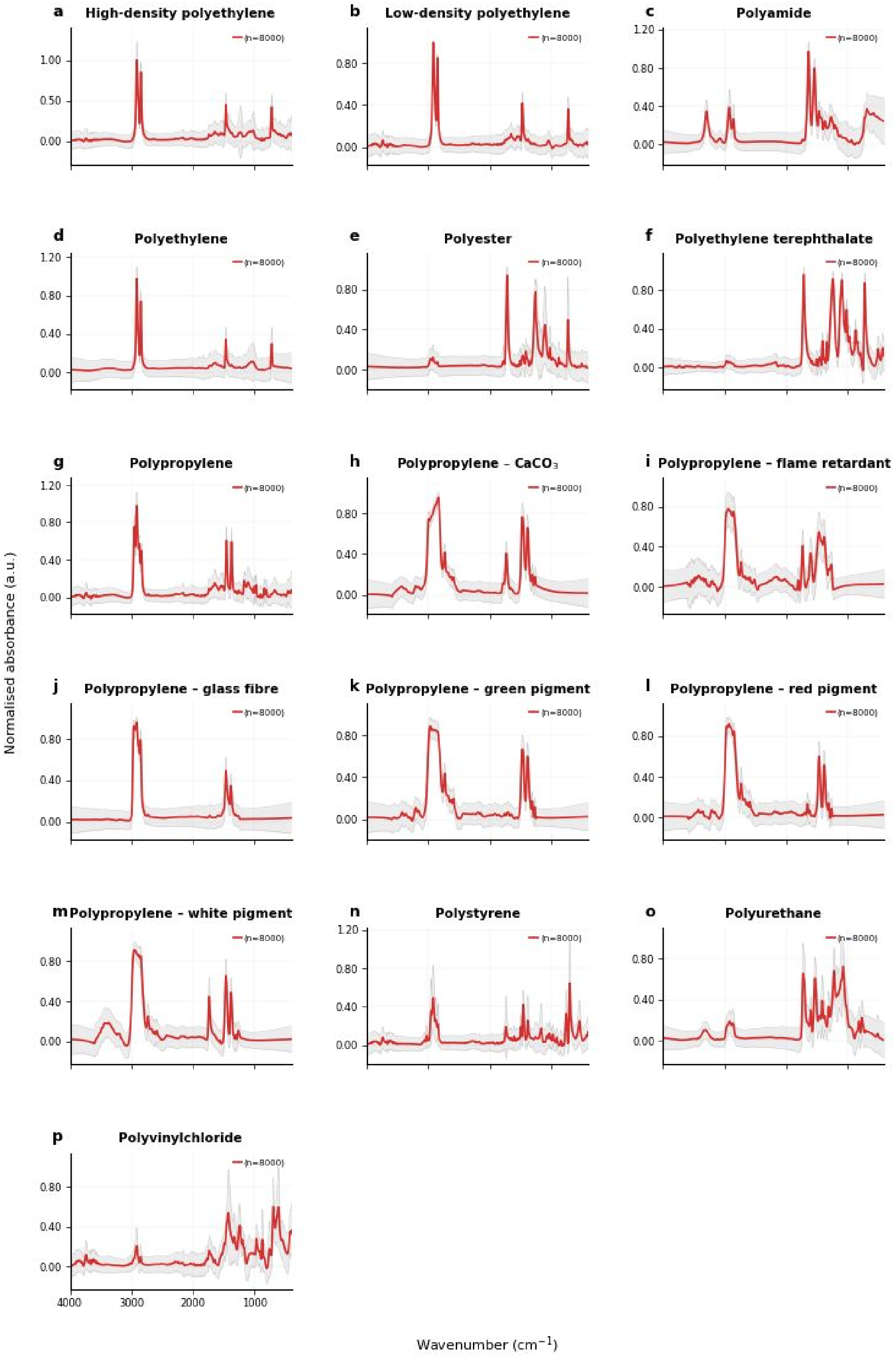

Figure S2. Normalised and baseline-corrected synthetic FTIR (Fourier Transform Infrared Spectroscopy) spectra for the 16 classes available for deep learning predictions included in

the Attenuated total reflectance-Fourier Transform Infrared Spectroscopy (ATR-FTIR) spectral library. The alphabetical class labels correspond to the following plastic types: High-density Polyethylene (HDPE; a), Low-density Polyethylene (LDPE; b), Polyamide (PA; c), Polyethylene (PE; d), Polyester (PES; e), Polyethylene terephthalate (PET; f), Polypropylene (PP; g), Polypropylene-CaCO3 pigment (PP-CaCO3; h), Polypropylene-flame retardant (PP-FR; i), Polypropylene-glass fibre (PP-GF; j), Polypropylene-green pigment (PP-GP; k), Polypropylene-red pigment (PP-RP; l), Polypropylene-white pigment (PP-WP; m), Polystyrene (PS; n), Polyurethane (PU; o), and Polyvinyl chloride (PVC; p).

3.2 Spurious peak removal

3.2.1 Cosmic Ray Removal

Cosmic rays can introduce sharp, high-intensity artefacts that mimic genuine Raman features.[44] A hybrid detection framework was developed, combining a rule-based detection stage and a second-stage ML model with PCHIP smoothing. The initial rule-based filter identified 49.1 ± 12.2% of artificially introduced cosmic rays in a validation dataset but struggled with broader or lower-intensity artefacts. To improve detection, RF and GB classifiers were trained as a second-stage enhancement. Hyperparameter tuning of RF and GB classifiers demonstrated strong performance on 15,536 spectra (7,689 cosmic ray, 7,847 non-cosmic ray; Table S11).

Table S11. Hyperparameter search for optimal prediction of cosmic rays against the presence of Raman spectral features

Parameters	Gradient Boosting				Random Forest			
	1	2	3	Aggregated	1	2	3	Aggregated
Class Weight	-	-	-	-	None	Balanced	None	None
Max depth	30	20	30	27	20	25	25	23
Max features	-	-	-	-	0.5	log2	0.5	0.5
Min samples leaf	4	4	4	4	1	1	1	1
Min samples split	2	20	20	4	5	2	2	3
N estimators	300	100	200	200	300	300	200	267
Learning rate	0.1	0.2	0.1	0.13*	-	-	-	-

***The aggregation process averaged the optimal parameters from the three iterations. Where the implementation requires integer values (e.g., N estimators), the mean value (133.33) was rounded to the nearest integer (133) for the final configuration. '-' denotes non-applicable parameters.**

RF achieved a weighted F1 of 0.9737 ± 0.0018, with recall of 97.37 ± 0.18% and precision of 97.38 ± 0.18%. GB slightly outperformed RF (weighted F1 0.975 ± 0.002, recall 97.46 ± 0.18%, precision 97.47 ± 0.19%) while requiring approximately half the optimisation time (29,677 s vs. 54,616 s), and was therefore selected for integration. Feature importance analysis of the GB model indicated FWHM (68.8%), derivative sharpness (8.9%), and asymmetry (7.8%) as the most informative predictors, with secondary contributions from z-score, peak height, and height-to-FWHM/prominence ratios.

When deployed in the hybrid two-stage cosmic ray detection pipeline, the GB classifier significantly improved artefact removal, identifying all residual cosmic rays missed by the initial PCHIP filter and producing near-zero spectral residuals suitable for downstream analysis (Figure S3).

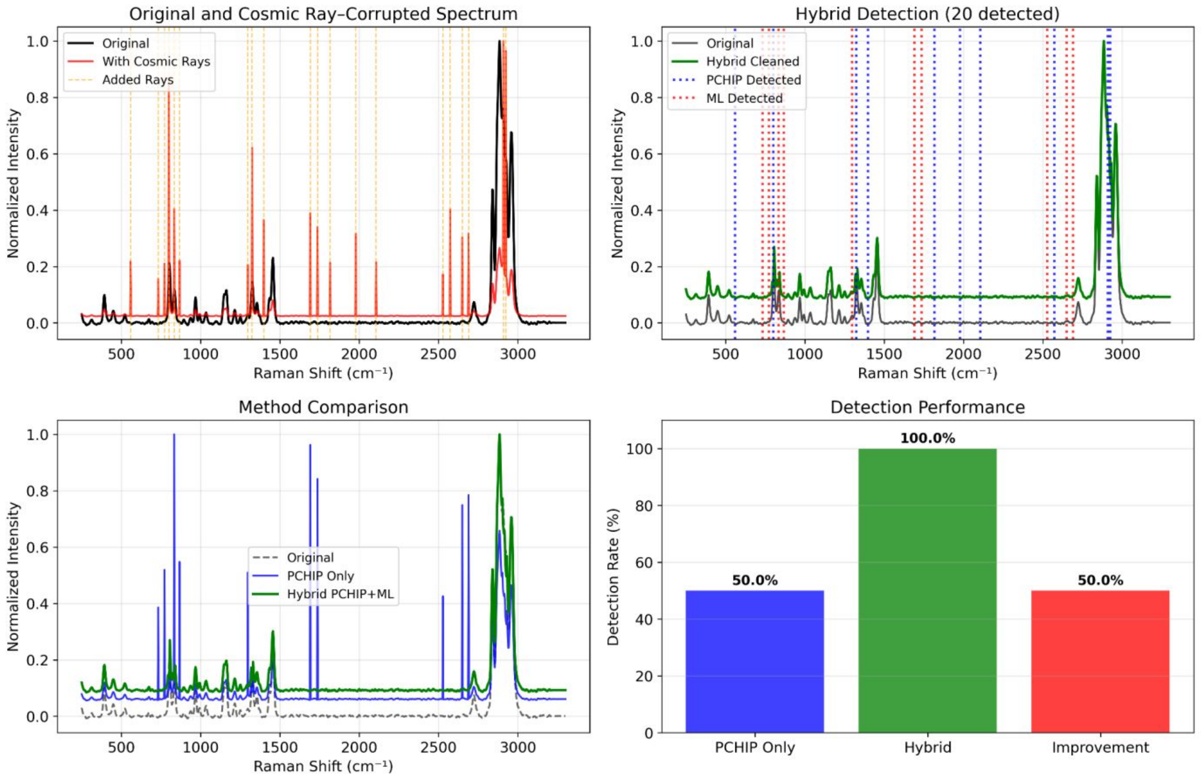

Figure S3. Additional demonstration of cosmic ray removal in a validation Raman spectrum of polypropylene (PP) using a hybrid framework combining rule-based detection, machine learning (ML), and Piecewise Cubic Hermite Interpolating Polynomial (PCHIP) correction. Twenty synthetic cosmic rays were injected across the spectrum, spanning positions from 558 to 2924 cm^{-1}, widths of 0.3–1.2 cm^{-1} (FWHM), and amplitudes 5.7–19.5× above the local signal. In this example, using synthetic cosmic rays inserted into the displayed PP spectrum, and applying a standard FWHM threshold of 1.0 cm^{-1}, the rule-based stage detected 12 of 20 cosmic rays (60%), with the ML enhancement stage identifying the remaining 8 (40%). The hybrid PCHIP+ML pipeline successfully detected and removed all 20 cosmic rays, achieving 100% removal efficiency.

Table S12 lists the synthetic cosmic ray parameters used for the separate PVC validation example presented in the main manuscript (Figure 3), whereas Figure S3 illustrates an additional PP-based demonstration of the hybrid detection pipeline.

Table S12. Spectral parameters of the twenty synthetic cosmic rays injected into the polyvinyl chloride (PVC) validation spectrum.

Ray ID	Position (cm⁻¹)	FWHM (cm⁻¹)	Absolute Amplitude	Relative Amplitude (× local)
1	866.7	1.02	874.4	7.8
2	772.1	0.84	737.9	7.3
3	1978.0	0.39	1129.7	11.9
4	1691.0	0.84	1525.5	15.6
5	2573.4	0.35	1520.6	15.8
6	1816.2	0.49	758.3	7.7
7	1397.9	0.86	1914.5	14.2
8	1324.7	0.69	2396.2	9.4
9	732.4	0.66	901.5	5.7
10	2689.4	0.63	1157.4	11.8
11	2649.7	0.86	1120.0	10.7
12	1297.2	0.83	1210.0	5.7
13	2527.6	0.45	580.6	6.0
14	1736.8	1.15	1314.3	13.4
15	2924.5	0.31	3300.0	8.5
16	833.1	0.70	938.5	6.8
17	2106.3	1.05	754.0	7.6
18	799.5	0.53	1507.6	14.9
19	558.4	0.68	1486.4	8.1
20	2909.2	0.47	3300.0	19.5

3.2.2 CO_2 Removal

CO_2 atmospheric contamination presents as characteristic double absorption bands centered at 2288 cm⁻¹ and 2392 cm⁻¹ in FTIR spectra. The algorithm employs PCHIP interpolation to replace the contaminated region with a continuous baseline that maintains consistency with the spectral trend at both boundary regions. The PCHIP-based CO_2 removal algorithm achieves near-complete removal (>97%) of CO_2 absorption features across a range of contamination intensities, spanning 0.20–0.90 and 0.15–0.71 relative absorption for synthetic bands centred at 2288 cm⁻¹ and 2392 cm⁻¹, respectively, as illustrated by the difference between the original contaminated (blue) and corrected spectra (red). Detailed boundary analysis at 2288 cm⁻¹ and 2392 cm⁻¹ illustrates a smooth transition between the original and reconstructed regions with no artificial discontinuities. This approach effectively eliminates atmospheric interference while preserving intrinsic polymer spectral features, allowing for more accurate material identification in subsequent analysis steps.

3.3 Chemometric analysis

3.3.1 Machine Learning dataset balancing

The spectral datasets exhibited substantial class imbalance. In the Raman and FTIR data, majority classes like Polyethylene terephthalate (PET), Polypropylene (PP), and Polystyrene (PS) were common, while minority classes were significantly underrepresented, such as Polyester, Polyurethane, and Polyamide. To address this imbalance, spectral augmentation using noise and baseline transformations was applied to minority classes and random under-sampling for majority classes, resulting in approximately 8,000 spectra per polymer class surpassing the 50 original spectra threshold for inclusion in the DRN training data (Figure S4 and S5). Figures S4 and S5 illustrate the effect of dataset balancing (using the proposed i-arPLS synthetic augmentation method; S2.8.1.2.3) on the polymeric classes for FTIR and Raman spectra, respectively.

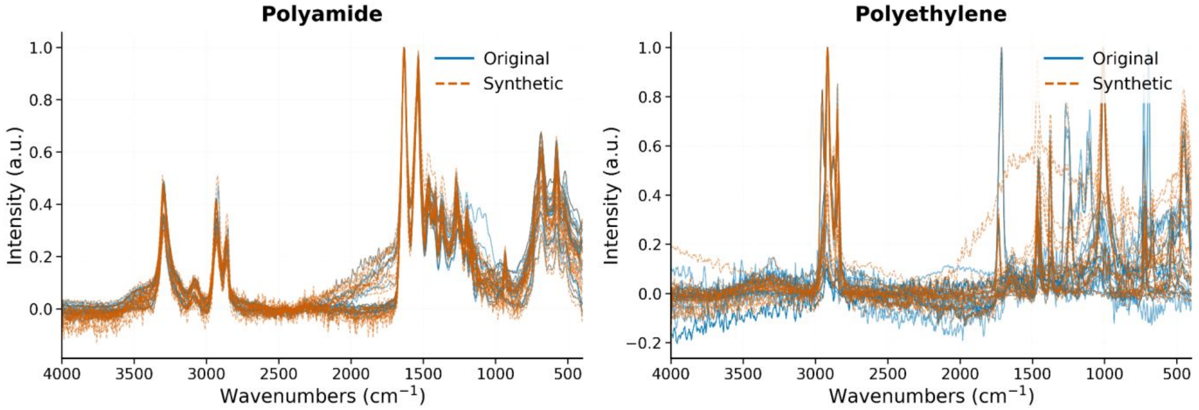

Figure S4. Original and synthetically generated Fourier Transform Infrared Spectroscopy-Attenuated total reflectance spectra for polyamide (left) and polyethylene (right). Displayed spectra have been subjected only to max normalisation and spectral alignment to ensure optimal graphical visualisation.

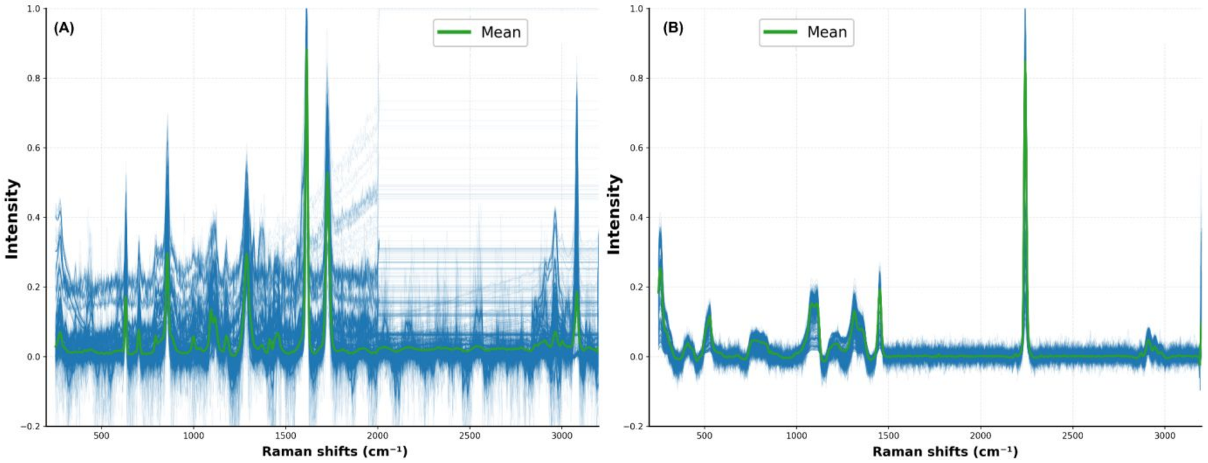

Figure S5. Original, non-mean-padded Raman spectra of Polyethylene terephthalate (A) and Polyacrylonitrile (B). Spectra were only max-normalised and spectrally aligned for optimal visual presentation.

3.3.1.1 Ablation Study: Data Augmentation and Training Set Size

To determine the optimal data augmentation strategy and training set size for the DRN, an ablation study was conducted evaluating three methods (original pipeline, i-arPLS pipeline, and SMOTE) across varying initial seed sizes and augmentation targets. Model performance was assessed using the macro F1-score and total accuracy on a strict, unaugmented held-out test set. The results (Table S13) demonstrate that the choice of augmentation method and the target dataset size influenced classification performance. The i-arPLS augmentation strategy consistently outperformed both the original noise-based pipeline and the SMOTE approach across comparable seed sizes. The optimal configuration was identified using the i-arPLS method with an initial seed size of 50 spectra per class, augmented to a target of 8000 spectra per class. This configuration yielded the highest overall performance, achieving a test accuracy of 0.9623 and a macro F1-score of 0.9648.

While the SMOTE strategy maintained competitive performance (achieving a peak F1-score of 0.9582 with a seed size of 61 and an augmentation target of 8000), it exhibited higher mean pairwise Pearson correlation coefficients ($r > 0.60$), indicating lower spectral diversity among the generated samples compared to the i-arPLS method. The original augmentation pipeline produced the most diverse spectra ($r < 0.06$) but resulted in lower classification accuracy. This indicates that baseline distortion introduced during augmentation likely degraded key class-specific spectral features. In contrast, the i-arPLS method preserved spectral structure while maintaining sufficient variability, and was therefore selected for all subsequent model training.

Table S13. Summary of ablation study results evaluating data augmentation strategies. Part A presents the macro-average performance across all tested seed sizes and augmentation targets for each method. Panel B details the single top-performing configuration for each respective strategy.

**PART**	**Augmentation Strategy**	**Mean spectral diversity (*r*)**	**Mean CV F1-Score**	**Mean Test Accuracy**	**Mean Test F1-Score**
A.sOverall method averages	i-arPLS pipeline	0.2891	0.9412	92.54	0.9138
	SMOTE	0.6724	0.9465	89.13	0.8799
	Original pipeline	0.0547	0.1834	84.36	0.8247
	**Seed Size**	**Seed Size / Target**	**CV F1-Score (± SD)**	**Test Accuracy**	**Test F1-Score**
B.wTop-performing configurations	i-arPLS pipeline	50 / 8000	0.9988 ± 0.0003	0.9623	0.9648
	SMOTE	61 / 8000	1.000 ± 0.000	0.9591	0.9582
	Original pipeline	50 / 1000	0.1175 ± 0.0558	0.9497	0.9313

Note: Lower Mean Spectral Diversity (r) values indicate greater variance and diversity among the synthetically generated spectra. The i-arPLS pipeline provided the optimal balance of spectral diversity and predictive accuracy

3.3.2 Machine Learning model selection

To evaluate available machine-learning models for spectral classification, five architectures were assessed: Standard CNN, LSTM, Transformer, DRN, and ICNN for both FTIR (Figure S6) and Raman (Figure S7).

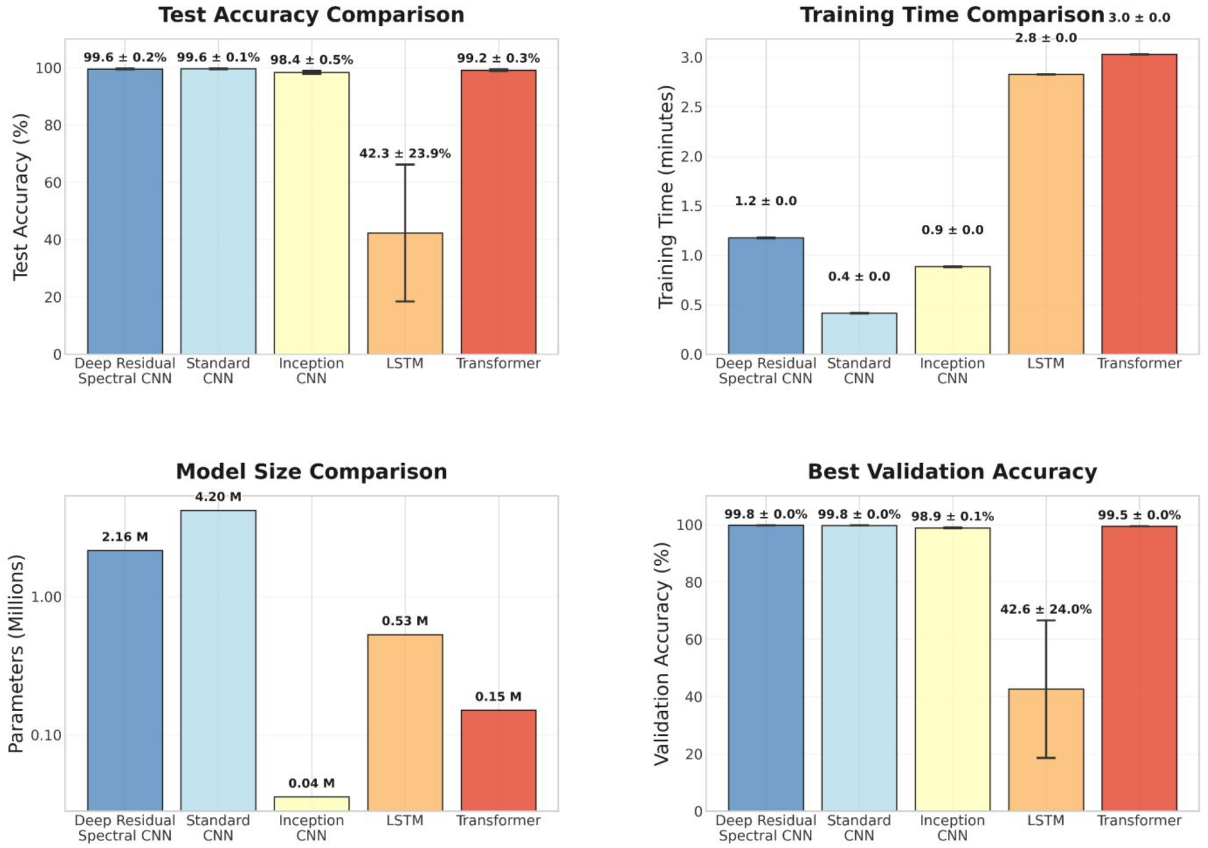

Figure S6. Comparative performance of machine learning architectures for FTIR plastic classification. (A) Test accuracy (mean ± s.d.) across three independent runs. Deep Residual Network (DRN; 99.99 ± 0.01%) achieved the highest accuracy, followed closely by Standard CNN (99.87 ± 0.09%) and Transformer (99.55 ± 0.10%). LSTM showed poor performance with high variance (69.89 ± 17.59%) due to failed training instances. (B) Training time comparison, highlighting computational efficiency. Standard CNN was the fastest to train (0.8 ± 0.0 min), while Transformer required the most time (5.5 ± 0.0 min). (C) Model size (logarithmic scale), ranging from 0.036 million parameters (Inception CNN) to 4.199 million (Standard CNN). (D) Best validation accuracy over training epochs, with patterns consistent with test accuracy metrics. All models were trained using identical settings: 8 epochs, learning rate of 0.001, batch size of 32, and the Adam optimiser. Error bars denote standard deviation across three runs.

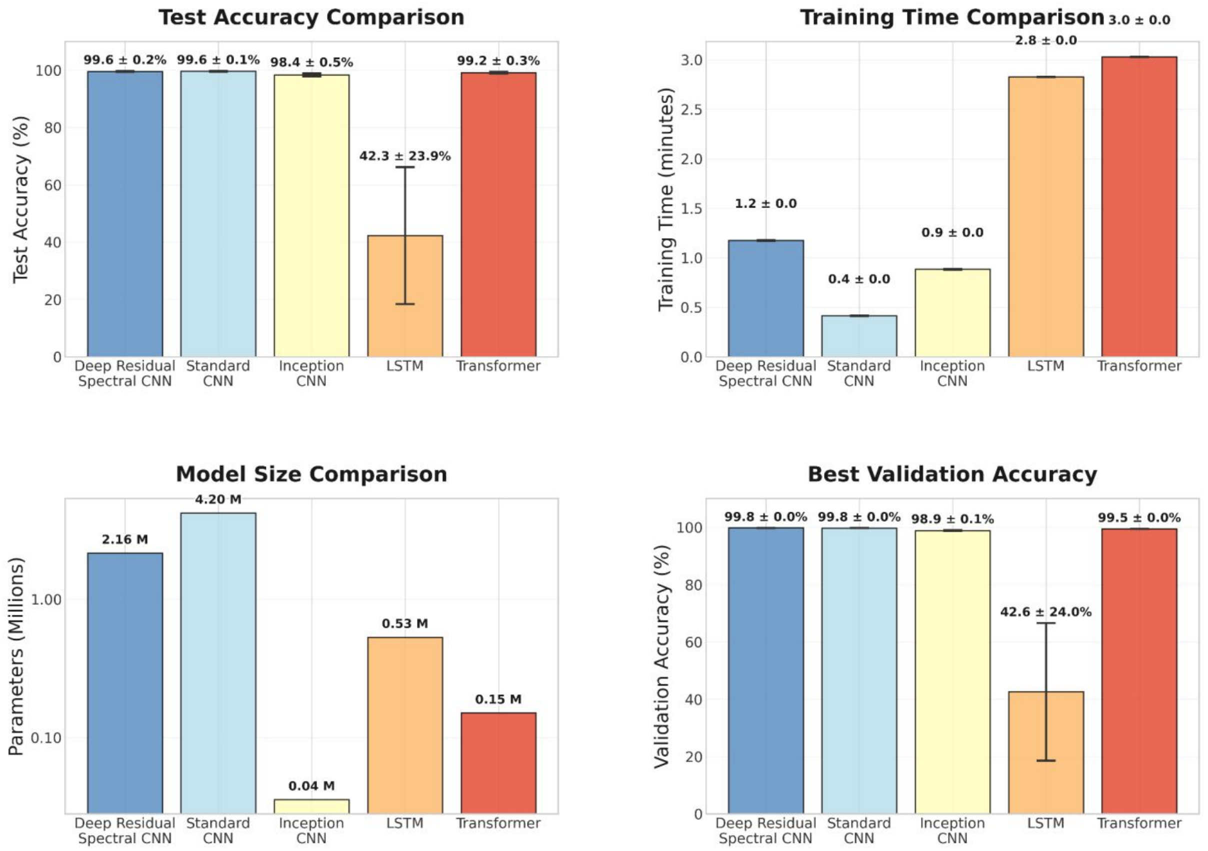

Figure S7. Comparative performance of machine learning architectures for Raman plastic classification. (A) Test accuracy (mean ± s.d.) across three independent runs. Standard CNN (99.63 ± 0.14%) achieved the highest test accuracy, followed closely by Deep Residual Spectral CNN (DRN; 99.56 ± 0.18%) and Transformer (99.16 ± 0.29%). LSTM showed poor performance with high variance (42.30 ± 23.88%) due to failed training instances. (B) Training time comparison, highlighting computational efficiency. Standard CNN was the fastest to train (0.4 ± 0.0 min), while Transformer required the most time (3.0 ± 0.0 min). (C) Model size (logarithmic scale), ranging from 0.036 million parameters (Inception CNN) to 4.199 million (Standard CNN). (D) Best validation accuracy over training epochs, with patterns consistent with test accuracy metrics. All models were trained using identical settings: 8 epochs, learning rate of 0.001, batch size of 32, and the Adam optimiser. Error bars denote standard deviation across three runs.

Table S14. Comparative performance metrics for different neural network architectures on FTIR and Raman spectroscopy datasets for polymer identification.

Model type	FTIR (Validation set)				Raman (Validation set)			
	Acc.	Prec.	Recall	F1-Score	Acc.	Prec.	Recall	F1-Score
Standard CNN	99.87 ± 0.09	99.87 ± 0.09	99.88 ± 0.08	99.87 ± 0.01	99.76 ± 0.02	99.65 ± 0.13	99.66 ± 0.12	99.65 ± 0.13
**DRN**	99.99 ± 0.01	99.99 ± 0.01	99.99 ± 0.01	99.99 ± 0.01	99.78 ± 0.04	99.59 ± 0.16	99.59 ± 0.17	99.58 ± 0.17
Spectral Transformer	99.55 ± 0.10	99.55 ± 0.10	99.55 ± 0.10	99.54 ± 0.10	99.51 ± 0.01	99.22 ± 0.27	99.22 ± 0.28	99.21 ± 0.28
Inception CNN	99.29 ± 0.37	99.30 ± 0.36	99.30 ± 0.36	99.29 ± 0.37	98.89 ± 0.14	98.49 ± 0.43	98.46 ± 0.52	98.46 ± 0.48
Spectral LSTM	69.89 ± 17.59	72.43 ± 16.72	69.52 ± 17.63	66.63 ± 20.49	42.62 ± 24.01	38.10 ± 27.40	42.41 ± 24.19	36.43 ± 28.43

***Note: All values are presented as mean ± standard deviation across three independent runs. Val. Acc., Prec., Recall, and F1 refer to mean ± standard deviation across three independent runs on the validation set used for architecture selection. These results are distinct from the final evaluation, where the optimised DRN was assessed on an unseen OOD-filtered test set (Raman: 96.9%, FTIR: 97.9%) as reported in Section 3.3.4.**

This model architecture comparison demonstrates that convolutional neural networks consistently outperform recurrent and transformer-based approaches for spectral polymer classification. Statistical analysis across multiple runs reveals strong performance of the DRN architecture for both FTIR and Raman spectroscopy applications, with the highest accuracy on FTIR and statistically equivalent performance on Raman. For FTIR data, DRN achieved exceptional accuracy (99.99 ± 0.01%), outperforming all other architectures including Standard CNN (99.87 ± 0.09%) and Transformer (99.55 ± 0.10%) as shown in Figure S6 and Table S14. For Raman spectra, DRN achieved the second-best performance (99.56 ± 0.18%) compared to Standard CNN (99.63 ± 0.14%), with the small accuracy difference (0.07%) and reported standard deviations indicating closely comparable performance. (Figure S7). The model demonstrated impressive performance while utilising approximately half the parameters (2.16M vs 4.20M) of the Standard CNN, indicating superior computational efficiency. The residual architecture's ability to maintain gradient flow through deeper networks likely contributes to this performance ceiling across both spectral domains. Furthermore, DRN demonstrates excellent generalisation between validation and test sets, suggesting robust real-world applicability. Given these characteristics across both spectral modalities and the established advantages of residual architectures for complex pattern recognition tasks, the DRN was selected as the model architecture for deployment in our polymer identification framework.

3.3.3 Model architecture and ensemble configuration

The CNN prediction pipeline presented utilises a one-dimensional CNN architecture. The model architecture, implemented in PyTorch, processes an input spectral vector through an initial feature extraction layer comprising a single one-dimensional convolution block, one-dimensional batch normalisation layer, and Rectified Linear Unit (ReLU) activation function. This layer feeds into a deep residual backbone constructed from a sequence of residual module blocks. Each residual module contains two one-dimensional convolution layers, which

utilise Dropout to prevent neuron co-adaptation and Batch Normalisation to stabilise internal layer activations. A key component of this module is an integrated Squeeze-and-Excitation attention mechanism. This block, applied prior to the residual addition, utilises one-dimensional adaptive average pooling (Squeeze) and two subsequent linear layers (Excitation) to compute and apply channel-wise attentional weights, thereby recalibrating feature-map importance. The residual identity (x + F(x)) is implemented via a shortcut connection, which employs a 1x1 convolutional layer to project the tensor x only when input and output channel dimensions are mismatched. Following the residual stack, a max-pooling layer performs spatial dimensionality reduction. The resulting feature map is flattened and processed by a terminal fully-connected (FC) head, composed of linear and Dropout layers, to produce the final classification logits. As detailed in Table S15, the hyperparameter optimisation yielded two distinct final configurations. The Raman model utilises a deeper architecture of 12 residual blocks, a larger initial kernel (k=11), and a pooling size (p=8), terminating in a two-layer FC head ([256, 128]). In contrast, the FTIR model is a more compact architecture with 8 residual blocks, a smaller initial kernel (k=3), a fine-grained pool size (p=2), and a single-layer FC head ([256]).For final deployment, these configurations are instantiated as 10-fold cross-validation ensembles. This strategy enhances predictive robustness by averaging the logits from five independent models, each trained on a unique data fold. The high-level data flow for these configurations is visualised in Table S15 and Figures S8.

Table S15. Optimal model hyperparameters determined via nested cross-validation. Following a random hyperparameter search, pairwise Wilcoxon signed-rank tests revealed a plateau of statistically equivalent, high-performing models. The final configurations presented here were selected from this top tier based on the highest absolute mean test accuracy (detailed in Tables S16 and S17).

**Model parameters**	**Raman**	**FTIR**
**Initial Channels**	32	128
**Initial kernel**	11	3
**Number of residual blocks**	12	8
**Channel progression**	32, 128, 256	16, 32, 64
**Kernel Size**	7, 11, 9, 11	7, 13, 3, 9
**Pool size**	8	2
**FC Layers**	256, 128	256
**Dropout rate**	0.38	0.22
**SE reduction**	16	16
**Learning Rate**	0.000485	0.0002393
**Early stopping patience**	7	7
**Average training epoch number**	39	23

**Batch size**	64	256

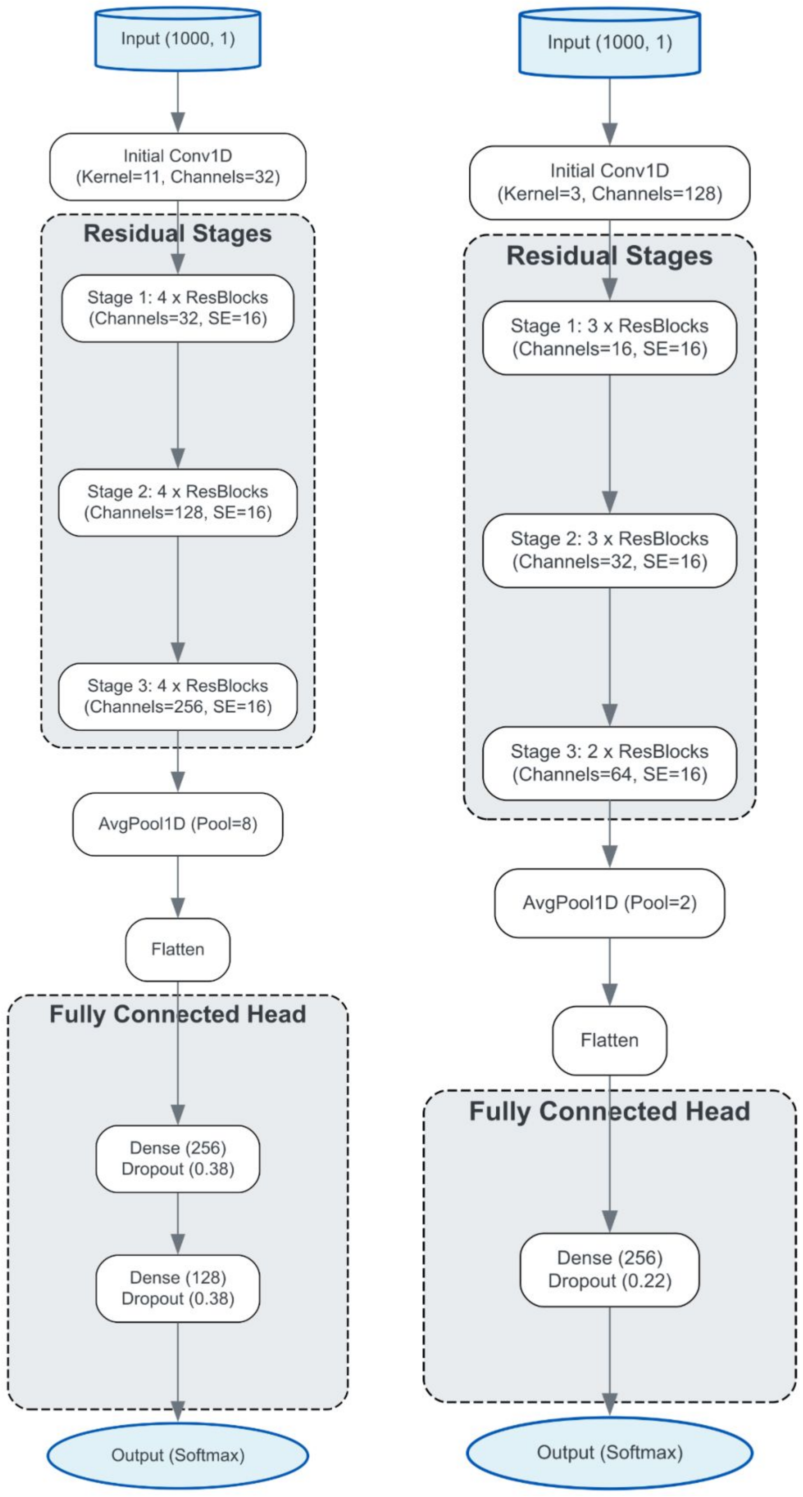

(a) (b)

Figure S8. Architecture of our proposed convolutional neural network. This visualisation provides a hierarchical view of the model's architecture, emphasising the flow of information from the input through convolutional, residual, pooling, and fully-connected layers to the final

output. Each layer's details, such as the type of operation and activation function, are annotated within the respective nodes.

3.3.3.1 Statistical analysis of top model configurations

To assess model performance robustness, statistical analyses including pairwise Wilcoxon signed-rank tests with Bonferroni correction and Spearman's rank correlation analysis were conducted across the top-performing configuration. The Wilcoxon signed-rank tests were used to compare the cross-validation fold results of the highest-ranked model against the subsequent top-performing contenders for both datasets. Bonferroni-corrected comparisons of Raman and FTIR hyperparameter responses showed that the differences exceeded the 0.05 significance threshold, indicating no statistically significant difference between the top-ranked model and the other leading configurations. This statistical equivalence is visually corroborated by the data in Tables S16 and S17.

Table S16. Performance summary for the top 10 Raman model configurations, ranked by mean test accuracy and including the 95% confidence interval (CI) and mean macro F1-score.

Configuration id	Mean test accuracy	Standard deviation of test accuracy	Confidence Intervals lower test accuracy	Confidence Intervals upper test accuracy	Mean macro F1 score
**9**	**0.996394**	**0.000328**	**0.996106**	**0.996682**	**0.996680**
11	0.996364	0.000643	0.995800	0.996927	0.996644
21	0.996273	0.000365	0.995953	0.996593	0.996562
20	0.996242	0.000249	0.996024	0.996461	0.996532
24	0.996242	0.000291	0.995987	0.996498	0.996534
10	0.996091	0.000826	0.995367	0.996815	0.996396
29	0.996061	0.000577	0.995555	0.996566	0.996367
2	0.996061	0.000597	0.995538	0.996583	0.996355
17	0.996030	0.000860	0.995277	0.996784	0.996336
7	0.996000	0.001075	0.995058	0.996942	0.996311

Table S17. Performance summary for the top 10 FTIR model configurations, ranked by mean test accuracy and including the 95% confidence interval (CI) and mean macro F1-score.

Configuration id	Mean test accuracy	Standard deviation of test accuracy	Confidence Intervals lower test accuracy	Confidence Intervals upper test accuracy	Mean macro F1 score
**11**	**0.999800**	**0.000162**	**0.999658**	**0.999942**	**0.999800**
24	0.999750	0.000156	0.999613	0.999887	0.999750
7	0.999717	0.000173	0.999565	0.999868	0.999717
9	0.999717	0.000254	0.999494	0.999939	0.999717
20	0.999650	0.000370	0.999326	0.999974	0.999650
10	0.999617	0.000173	0.999465	0.999768	0.999616
18	0.999617	0.000267	0.999382	0.999851	0.999617
4	0.999583	0.000228	0.999383	0.999783	0.999583
19	0.999567	0.000379	0.999234	0.999899	0.999567
21	0.999517	0.000375	0.999188	0.999845	0.999516

Tables S16 and S17 display the close performance metrics, with overlapping confidence intervals (CI) for mean test accuracy (mean test acc.) and minimal variation in mean macro F1-scores across the top models. Furthermore, the box and whisker plots in Figures S9 and S10 clearly illustrate this finding, showing heavily overlapping performance distributions for the top 10 configurations.

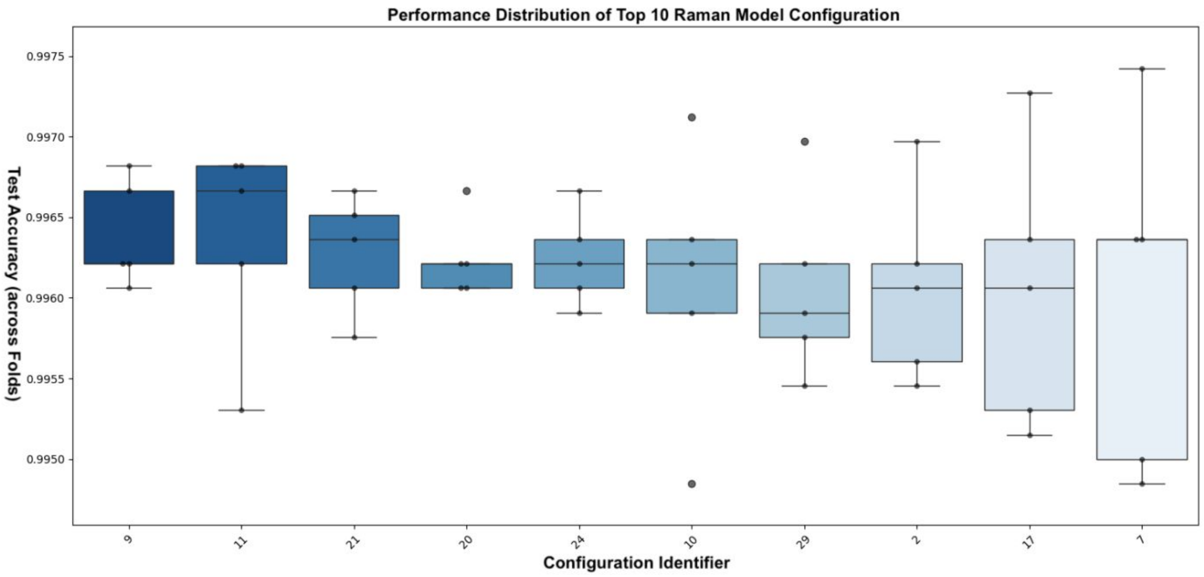

Figure S9. Performance distribution of the top 10 Raman configurations across all cross-validation folds.

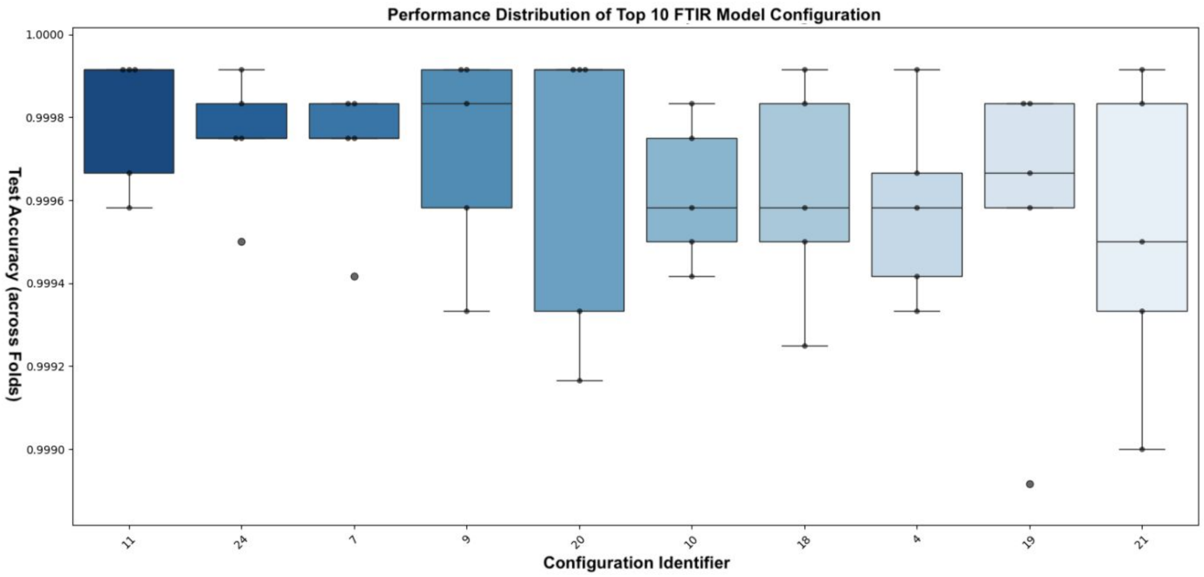

Figure S10. Performance distribution of the top 10 FTIR configurations across all cross-validation folds.

Given this statistical equivalence, configurations 9 (Raman) and 11 (FTIR) were selected for the final analysis as representative high-performing models with the highest observed mean test accuracy.

A Spearman's correlation analysis (Table S18) was conducted to investigate the relationship between individual hyperparameters and model performance. The results confirmed that no single parameter had a dominant effect, with all correlation coefficients falling within the weak-to-moderate range (< 0.5). Both datasets exhibited a similar moderate positive correlation with Early stopping patience ($p = 0.387$) and weaker positive trends with Learning rate and Number of epochs. The primary differences emerged in the negative correlations. Raman performance showed weak-to-moderate negative correlations across several architectural parameters, most notably Initial kernel ($p = -0.295$), Initial Channels ($p = -0.289$), and Number of residual blocks ($p = -0.287$). In contrast, FTIR performance was largely uncorrelated with most parameters, with its strongest negative trend being a weak association with Initial Channels ($p = -0.224$) and Pool size ($p = -0.220$).

3.3.4 A Hierarchical Framework for Efficient Out-of-Distribution Detection

3.3.4.1 Deep learning discrimination of polyethylene and its confounding counterparts

The mean Raman spectra (±1 standard deviation) for all PE confounding classes are presented in Figure S11, each comprising 2,000 spectra.

Table S18. Spearman's Rho (p) correlation coefficients between model hyperparameters and mean test accuracy for the FTIR and Raman datasets. Abbreviation: Fourier Transform Infrared Spectroscopy, FTIR; Raman microscopy, Raman.

**Configuration parameter**	**FTIR Spearman's Rho**	**Raman Spearman's Rho**
Early stopping patience	0.3874	0.3874
Learning rate	0.1854	0.2501
Number of epochs	0.1705	0.2134
Dropout rate	0.0075	-0.1624
Number of residual blocks	0.0029	-0.2866
Initial kernel	-0.0312	-0.2952
Batch size	-0.0612	-0.2823
Squeeze-and-Excitation reduction	-0.1604	-0.2577
Pool size	-0.2204	-0.2271
Initial Channels	-0.2243	-0.2891

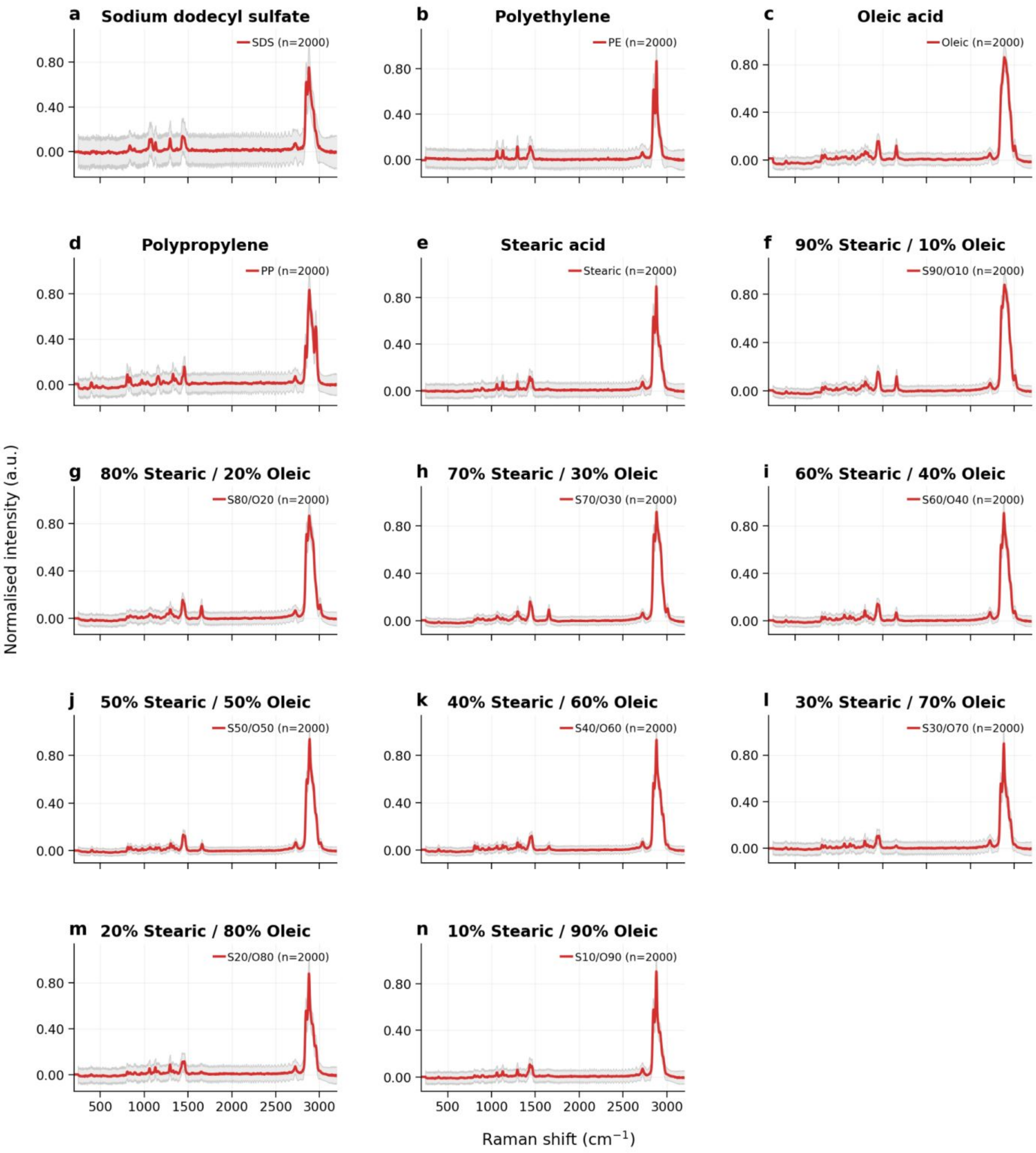

Figure S11. Normalised mean Raman spectra (solid red lines) with ±1 standard deviation (grey shaded regions) for the 14 classes included in the Raman spectral library (n = 2,000 spectra per class). Subplots correspond to: (a) Sodium Dodecyl Sulphate (SDS), (b) polyethylene (PE), (c) oleic acid, (d) polypropylene (PP), (e) stearic acid, and (f–n) binary stearic acid–oleic acid mixtures at mass ratios of 90:10, 80:20, 70:30, 60:40, 50:50, 40:60, 30:70, 20:80, and 10:90, respectively. Spectra are displayed over the 300–3,200 cm^{-1} Raman shift range.

3.3.4.2 Dataset linearity

The mean inter-class PCCs were computed for confounding PE class combinations to assess the degree of spectral linearity within the dataset (Figure S12). The resulting correlation matrix revealed uniformly high inter-class correlations, with the vast majority of values exceeding r = 0.93 and all values remaining above r = 0.82. The highest correlations were observed among

the binary stearic acid–oleic acid (SA:OA) mixtures, where adjacent mixing ratios routinely approached r = 1.00, indicating near-identical mean spectral profiles. Even the most spectrally distinct class, PE, exhibited its lowest correlation of r = 0.82 against PP, which still constitutes a strong positive relationship. This high degree of linear similarity across all classes confirms that the classification task is inherently challenging, as the spectral differences between classes are subtle and largely confined to small intensity variations rather than the presence or absence of distinct spectral features.

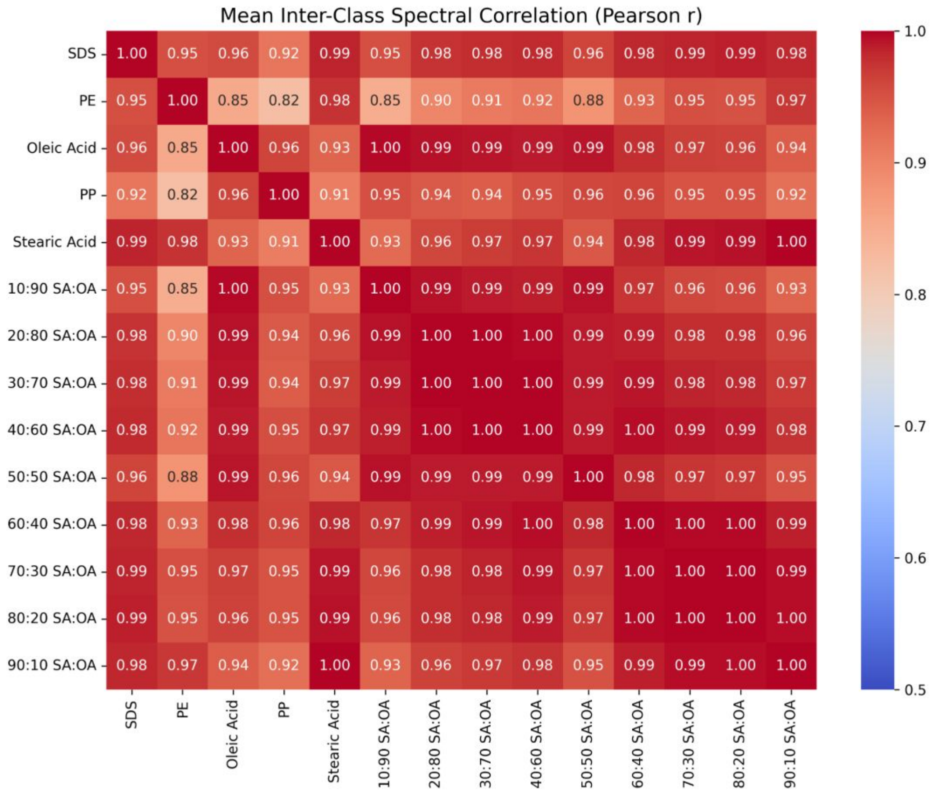

Figure S12. Mean inter-class Pearson correlation matrix (r) for the 14-class Raman spectral dataset. Each cell represents the pairwise correlation between the mean spectra of two classes. Class labels denote the five pure compounds, sodium dodecyl sulphate (SDS), polyethylene (PE), oleic acid, polypropylene (PP), and stearic acid, alongside nine binary stearic acid–oleic acid (SA:OA) mixtures at mass ratios ranging from 90:10 to 10:90. The colour scale spans r = 0.5 to 1.0, with darker red indicating higher spectral similarity.

3.3.4.3 Model hyperparameter optimisation

Hyperparameter optimisation was performed to identify the optimal architectural and training configurations for each deep learning model evaluated. The proposed DRN and Advanced U-Net architectures were tuned using the optimisation framework described in Section 2.8.3.3, while the published U-Net was implemented using its fixed, literature-defined parameters. Optimal configurations were selected based on cross-validated performance, balancing classification accuracy and generalisation. The resulting model parameters are summarised in Table S19.

Table S19. Optimised architectural and training hyperparameters for the proposed DRN, Advanced U-Net, and published U-Net models

Model parameters	DRN proposed here	Advanced U-Net	Published U-Net
**Initial Channels**	32	64	-
**Initial kernel**	11	7	3
**Kernel Sizes**	3	7, 9, 11	-
**Number of residual blocks**	12	8	8
**Channel progression**	32, 64, 128, 128, 128, 128,128,128,128,128, 128,128	64, 128, 256, 256, 256, 256, 256, 256	-
**Pool size**	8	8	-
**SE reduction**	16	8	-
**Learning Rate**	0.001	0.00005 (5e-05)	0.001
**Dropout rate**	0.1	0.2	-
**Max epoch number**	37	33	30
**Patience**	7	7	5
**Batch size**	128	32	512
**FC Layer**	128, 64	256, 128	-

3.3.4.4 Model classification evaluation

Prior to the ablation study, the class-level predictive performance of the proposed DRN (Figure S13) was evaluated for spectral class predictions and benchmarked against the Advanced U-Net architecture (Figure S14) and Lim et al. [35]'s published U-Net (Figure S15). To explicitly quantify these differences, per-class F1-scores were compared between the proposed DRN and the baseline U-Net (Table S20). The DRN outperformed the published architecture in 11 of 14 classes, with the most significant performance gains observed in highly ambiguous binary mixtures.

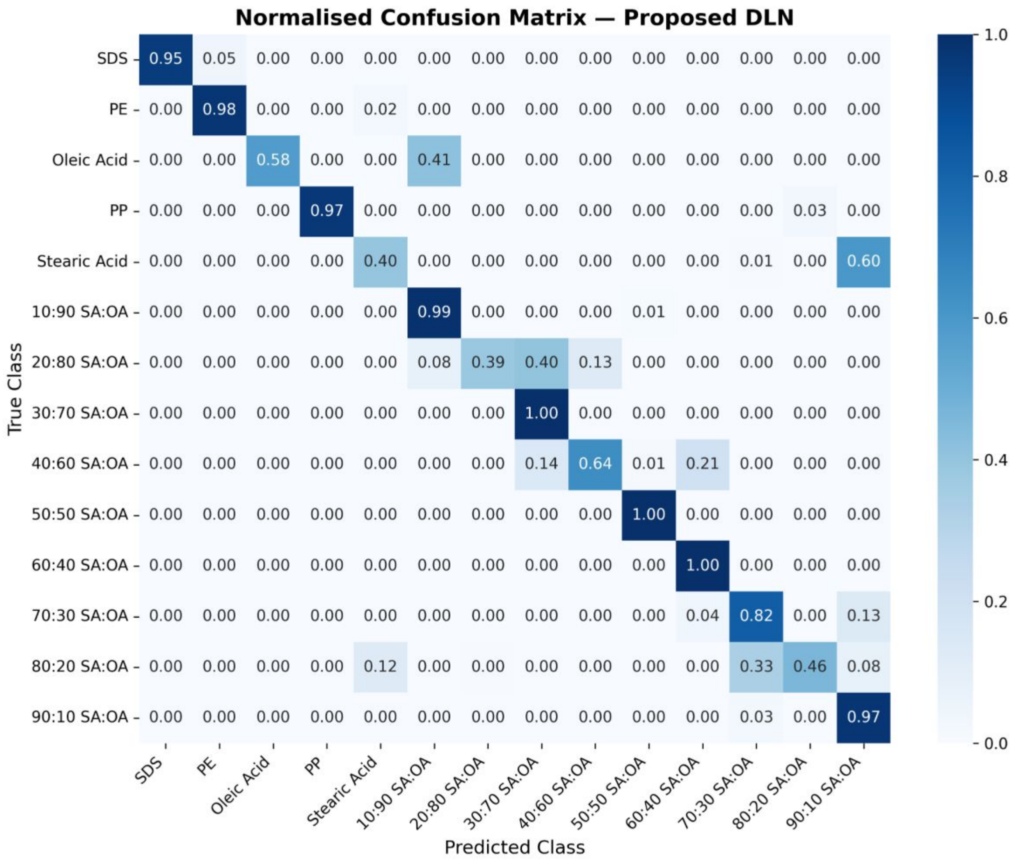

Figure S13. Normalised confusion matrix illustrating the classification performance of the proposed Deep Residual Network (DRN). The matrix displays the true versus predicted classification rates across pure compounds (SDS, PE, PP, Oleic Acid, Stearic Acid) and varying concentration ratios of Stearic Acid to Oleic Acid (SA:OA) mixtures. The diagonal values represent the proportion of correctly predicted instances for each respective class.

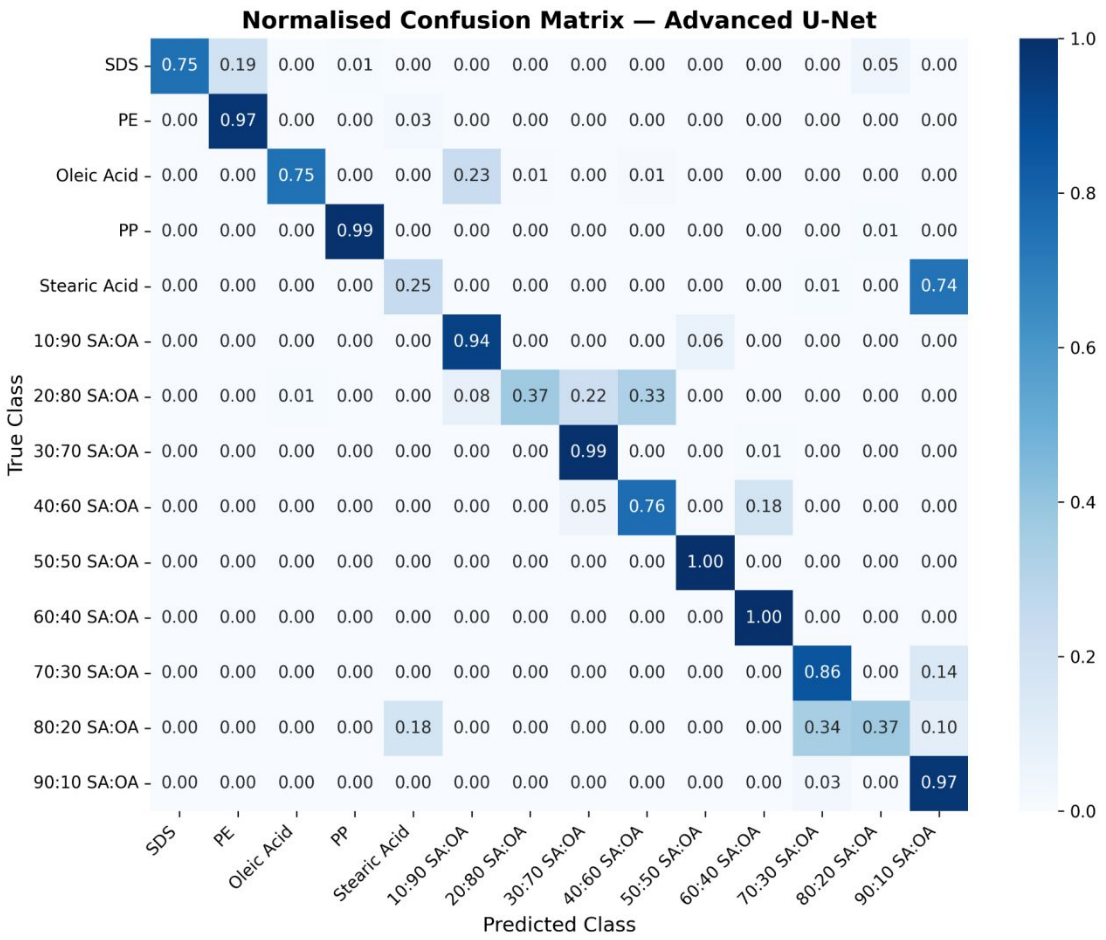

Figure S14. Normalised confusion matrix illustrating the classification performance of the proposed Advanced U-Net. The matrix displays the true versus predicted classification rates across pure compounds (SDS, PE, PP, Oleic Acid, Stearic Acid) and varying concentration ratios of Stearic Acid to Oleic Acid (SA:OA) mixtures. The diagonal values represent the proportion of correctly predicted instances for each respective class.

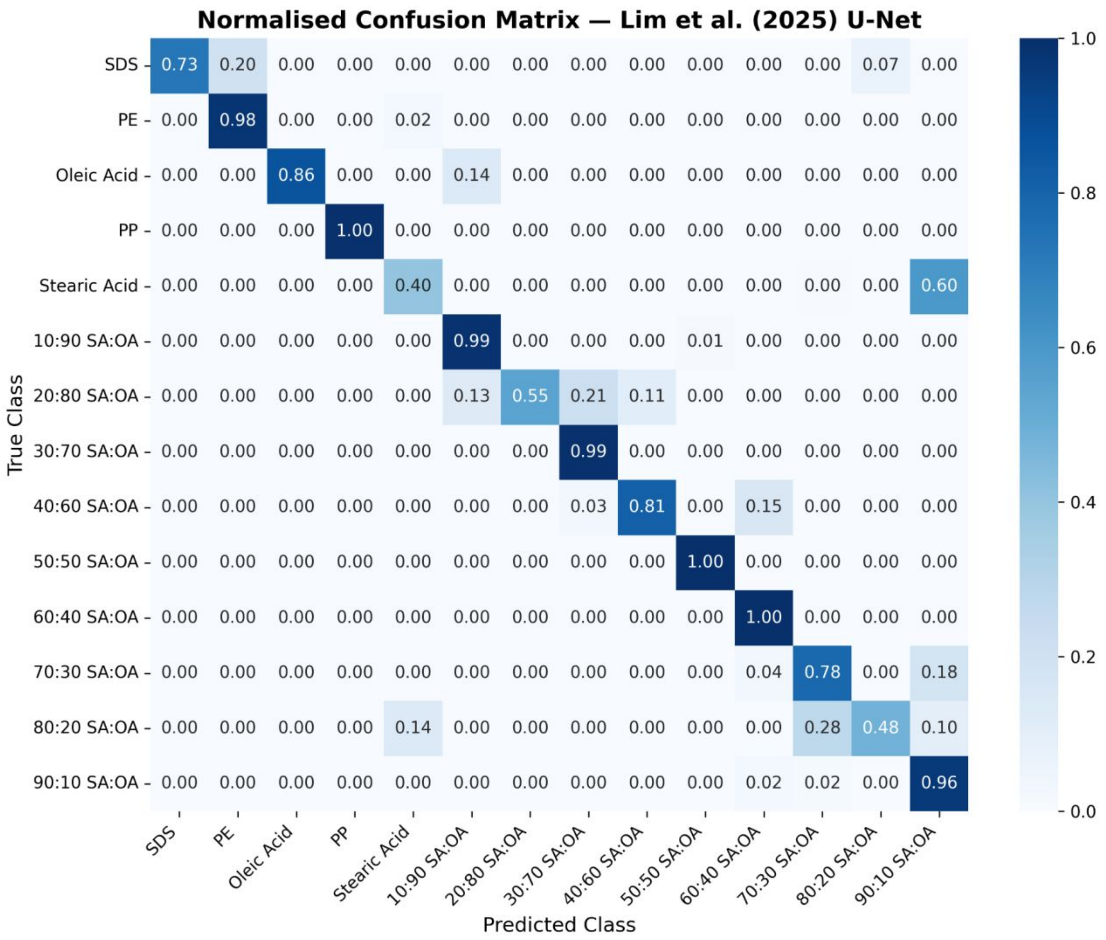

Figure S15. Normalised confusion matrix illustrating the classification performance of the published Lim et al. [35] published U-Net. The matrix displays the true versus predicted classification rates across pure compounds (SDS, PE, PP, Oleic Acid, Stearic Acid) and varying concentration ratios of Stearic Acid to Oleic Acid (SA:OA) mixtures. The diagonal values represent the proportion of correctly predicted instances for each respective class.

3.3.4.5 Baseline: DRN Ensemble Confidence

The 10-fold DRN ensemble exhibited clear discrimination between ID and OOD samples for Raman spectroscopy, while performance was comparatively weaker for FTIR data. For ID spectra, the ensemble achieved perfect classification accuracy for both modalities (Raman: 100%; FTIR: 100%). This was accompanied by high mean predictive confidence, reported in probability space (0–1 scale), with Raman reaching 0.9984 ± 0.0133 and FTIR reaching 0.9999 ± 0.0013. These values indicate near-saturated softmax outputs under ID conditions. When applied to out-of-distribution (OOD) spectra, the ensemble failed to correctly classify any samples (0% accuracy across both Raman and FTIR). Despite this, the model frequently produced overconfident predictions, particularly for FTIR OOD spectra. Mean prediction confidence for OOD samples remained elevated and only slightly lower relative to in-distribution data (Raman: 0.8778 ± 0.1736 [range: 0.5100–1.0000]; FTIR: 0.9499 ± 0.1214 [range: 0.4767–1.0000]). This indicates systematic overconfidence under distribution shift, particularly for FTIR spectra.

Table S20. Per-class classification performance (F1-score) comparison between the proposed Deep Residual Network (DRN) and the published U-Net architecture. The evaluation demonstrates the models' ability to disambiguate polyethylene (PE) from highly correlated confounding chemicals and mixtures. The DRN outperformed the baseline U-Net in 11 of the 14 classes, with the most significant performance gains observed in highly ambiguous binary mixtures (e.g., 90:10 Stearic Acid:Oleic Acid).

Chemical Composition	Proposed DRN (F1)	Published U-Net (F1)	Δ (DRN - U-Net)
Sodium Dodecyl Sulphate	0.9882	0.9865	+0.0017
Polyethylene	0.9885	0.9900	-0.0016
Oleic Acid (OA)	0.9801	0.9688	+0.0113
Polypropylene	1.0000	0.9900	+0.0100
Stearic Acid (SA)	0.8447	0.8432	+0.0015
90:10 SA:OA	0.9748	0.9519	+0.0229
80:20 SA:OA	0.9508	0.9455	+0.0053
70:30 SA:OA	0.9816	0.9784	+0.0032
60:40 SA:OA	0.9351	0.9201	+0.0150
50:50 SA:OA	0.9934	0.9819	+0.0114
40:60 SA:OA	0.9704	0.9612	+0.0092
30:70 SA:OA	0.9012	0.9106	-0.0094
20:80 SA:OA	0.9157	0.9313	-0.0157
10:90 SA:OA	0.8317	0.8214	+0.0103

Table S21. Overall classification performance comparison between the proposed Deep Residual Network (DRN) and the baseline U-Net. Performance is evaluated using the global average Test F1-score on the held-out dataset and the mean Cross-Validation (CV) F1-score (± standard deviation). The results demonstrate that the proposed DRN achieves superior overall predictive accuracy and generalisation compared to the published baseline architecture.

Metrics		Proposed DRN (F1)	Published U-Net (F1)	Δ (DRN - U-Net)
Test F1	Global Average	0.9469	0.9415	+0.0054
CV F1	Cross-Validation Average	0.9558 ± 0.0018	0.9524 ± 0.0017	+0.0034

Using mean prediction confidence as a naive OOD detection criterion yielded moderate to strong separability, with ROC AUC values of 0.956 for Raman and 0.846 for FTIR. However, this apparent performance masked a limitation: the confidence distributions of ID and OOD

samples substantially overlap, particularly for FTIR, where ID confidence (0.9999 ± 0.0013) and OOD confidence (0.9499 ± 0.1214) are not cleanly separable under simple thresholding.

This overlap demonstrates that while confidence-based scoring can provide partial discrimination between ID and OOD samples, it is not sufficiently robust for reliable deployment. The results therefore point to the need for a more advanced, multi-feature OOD detection framework that accounts for spectral heterogeneity across instrument modalities.

3.3.4.6 Uncertainty-aware Random Forest classifier

A hierarchical framework for OOD detection was developed to ensure DRN predictions are applied only to ID Raman and FTIR spectra. The second stage of this framework uses an uncertainty-aware RF classifier, which incorporates both DRN-derived metrics and PCC scores as inputs to detect potential OOD samples.

The RF OOD classifier was trained to analyse the output statistics from both the primary DRN ensemble and the PCC library search. The input features for this RF model were selected from a pool of 28 engineered metrics, with the optimal set for the Raman model consisting of 6 features, and 7 features for the FTIR model. This set combined DRN uncertainty metrics (including calibrated confidence and total uncertainty) with PCC-derived metrics (including correlation best match score and hybrid confidence). These distance metrics were computed within a dimensionality-reduced embedding space, generated by applying PCA to the raw DRN embeddings. For the Raman model, 212 components (capturing 99% of the explained variance) were used, while 1601 components (99% of explained variance) were used for the FTIR model. The Mahalanobis distance, in particular, was calculated using the saved PCA transformer model, a pre-computed set of class centroids, and their inverted shared covariance matrix.

The RF was trained as a binary classifier, where the ground truth target was 'ID' (label 1) only if two conditions were met: (1) the spectrum was manually verified as belonging to an ID class, and (2) the DRN's classification for that spectrum was correct. If either condition failed (i.e., the spectrum was true OOD, or it was a misclassified ID sample), the ground truth target was set to 'OOD' (label 0). An RF model hyperparameter search optimisation was completed in line with prior models shown in this publication (Table S22).

Table S22. Optimised hyperparameters for the Out-of-Distribution (OOD) Random Forest classifiers applied to the Raman and FTIR datasets.

Parameters	Raman	FTIR
**Number of estimators**	50	50
**Max depth**	5	7
**Min samples split**	4	8
**Min samples leaf**	2	2
**Max features**	sqrt	sqrt
**Bootstrap**	False	False

A key finding was that the optimal OOD detection strategy differed significantly between the Raman and FTIR models. We analysed the Gini importance of each feature in the trained RF classifiers to determine which metrics were most predictive of an OOD sample (Table S23).

For the Raman-based RF classifier, internal uncertainty metrics were exclusively dominant. Interestingly, no PCC-based metrics were selected in the optimal top-6 feature set for Raman. The most important feature was Total Uncertainty (0.246), followed by Distance Weighted Confidence (0.194), confirming that the deep learning embeddings provided sufficient signal for outlier detection without external library validation (Table S23).

In contrast, the FTIR-based RF classifier relied heavily on geometric distance in the embedding space, supported by a hybrid of DRN confidence and external library-matching metrics. The top-ranked feature was Mahalanobis distance (0.238), followed by calibrated confidence (0.179) and hybrid confidence of PCC (0.175). This divergence underscores that while Raman OOD detection can be driven by internal softmax entropy and uncertainty, FTIR OOD detection benefits significantly from measuring the geometric distance of samples from the training distribution centroids.

Table S23. Top-ranking feature importance for OOD Random Forest classifiers. Gini importance scores for the top features used by the RF models to classify spectra as In-Distribution (ID) or Out-of-Distribution (OOD). Abbreviation: Fourier Transform Infrared Spectroscopy, FTIR; Raman microscopy, Raman.

Feature Rank	Raman	FTIR
1	Total uncertainty (0.246)	Mahalanobis distance (0.238)
2	Distance weighted confidence (0.194)	Calibrated confidence (0.179)
3	Uncertainty weighted confidence (0.173)	Hybrid confidence correlation (0.175)
4	Confidence variation product (0.136)	Correlation best match score (0.174)
5	Confidence entropy ratio (0.133)	Correlation Margin 1 vs 2 (0.141)
6	Calibrated confidence (0.119)	Correlation and DRN match score (0.074)
7	None (Only 6 features were selected)	Correlation match 1 (0.018)

3.3.4.7 Final Hierarchical PCC and RF Framework for Out-of-Distribution Detection

The performance of this integrated framework was quantified on the held-out test datasets (Raman: n=104; FTIR: n=104). Two distinct metrics were established to evaluate its practical utility and safety profile. First, *strict accuracy* was defined as the classification accuracy where any sample triaged for 'Verification' is considered a non-automated prediction (reflecting the automation rate). Second, *safety* was evaluated by the number of OOD spectra incorrectly accepted as ID. The framework's performance is presented in Table S24.

Table S24. Final Hierarchical Framework Performance on Held-Out Test Data. Performance metrics evaluate the safety and automation efficiency of the Out-of-Distribution (OOD) detection pipeline. *PCC Screen-Only Accuracy* indicates the baseline filtering performance using only Pearson correlation. *Strict Accuracy (Fully Auto)* defines the true automation rate, representing the percentage of spectra processed correctly without human intervention (spectra flagged for verification are counted as incorrect). *Post-Verification Accuracy* reflects the final accuracy after a human operator reviews the flagged data. *ROC AUC* measures the Random Forest classifier's ability to separate In-Distribution (ID) from OOD spectra. *OOD Spectra Incorrectly Accepted* represents the critical safety fail rate, showing the exact number of anomalous spectra that bypassed the screening framework. Abbreviations: Fourier Transform Infrared Spectroscopy, FTIR; Raman microscopy, Raman.

	PCC Screen-Only Accuracy	Strict Accuracy (Fully Auto)	Post-Verification Accuracy	ROC AUC	OOD Spectra Incorrectly Accepted
FTIR	85.58%	52.4%	61.9%	0.891	2 / 52
Raman	96.15%	81.00%	100.0%	0.956	0 / 32

The OOD framework demonstrated exceptional efficacy for Raman spectra, achieving 100.0% Test Accuracy on the held-out set with an 81.0% Automation Rate. Critically, it achieved perfect safety, with zero (0/32) true OOD spectra being incorrectly accepted as ID; all challenging OOD samples were correctly routed to the 'Verify' or 'Reject' bins. Performance on the FTIR dataset highlighted the challenge of distribution shifts in the OOD class (KS test p=0.003), resulting in a more conservative 52.4% Automation Rate and 61.9% Test Accuracy. While the system maintained a high safety profile, a small number of OOD spectra (2/52) were incorrectly accepted. The significant shift indicates that the test OOD samples represent a domain not fully covered by the training set. This suggests that while the hierarchical framework is highly effective for Raman spectroscopy, the FTIR modality may require larger training datasets or tighter thresholds to handle significant distribution shifts between training and deployment environments. To ensure the OOD framework continually improves, outcomes of OOD acceptance and their corresponding classifications will be anonymously stored, subject to user consent, and used to further train the system.

3.3.5 Prediction classification comparison

The strength of our DRN model stems from its training dataset, which includes a diverse array of spectra from environmental, virgin, and consumer plastics. This diversity allows the model to learn the 'general' spectral trends associated with polymers, rather than only pristine reference signatures. As a result, our model is more robust for analysing weathered or environmental samples compared to commercial libraries that contain solely 'virgin' spectra of the highest quality.

This advantage is demonstrated by applying our model to a held-out dataset of Raman spectra collected by Dong et al. [9], which were originally analysed using the commercial Bio-Rad library. Table S25 compares the Bio-Rad Hit Quality Index (HQI) score for these spectra against the predictions from our DRN model. All samples shown were successfully accepted as ID by our two-stage OOD framework before prediction.

Table S25. Comparison of commercial library HQI scores (Bio-Rad) versus the confidence of the developed DRN model for spectra from Dong et al. [9]. Abbreviations: polyethylene, PE; polypropylene, PP; polyethylene terephthalate, PET; and polyvinyl chloride, PVC. Spectra from Dong et al. [9].

File name	Composition ground truth	Bio-Rad Score	ML Prediction	ML Prediction Confidence
wea-156.txt	PVC	70.1	PVC	1
sta-3.txt	PET	80.76	PET	1
wea-168.txt	PP	87.45	PP	0.9999412298
wea-71.txt	PE	77.68	PE	0.999375999
sta-10.txt	PVC	84.67	PVC	1
wea-135.txt	PP	51.21	PP	1
sta-4.txt	PET	54.33	PET	1

The table illustrates that while the commercial library returned low-confidence matches (e.g., HQI scores as low as 51.21 and 54.33) for several spectra, our DRN model correctly and confidently identified the same polymers with near-perfect confidence scores. In these specific cases, our DRN model consistently provided the correct polymer composition with high confidence (mean confidence: 1.0 ± 0.0; Table S25).

3.3.6 Spectral image analysis

3.3.6.1 Background identification and removal

This section outlines the results of the enhanced spectral image pipeline utilising an RF classifier in combination with K-means clustering and PCA for the identification and subtraction of background spectra, facilitating the downstream spectral interrogation of microplastics. Analysis was performed on both plastic-only and environmental FTIR and Raman spectral images, comprising 17 and 22 images, respectively. These 17 FTIR and 22 Raman images formed the labelled training dataset; inference performance was subsequently evaluated on a larger held-out set of 53 FTIR and 25 Raman images (Section 3.3.6.1.3). Image labeling comprised annotating particle presence, with background pixels assigned to the substrate, yielding 91,995 labelled pixels for FTIR (62,678 background; 29,317 sample) and 35,680 for Raman (24,277 background; 11,403 sample). The manual annotation coverage was 22.50% and 34.35% of FTIR and Raman images used in the training dataset. Optimal hyperparameters were identified via a grid search of 768 parameter sets using 5-fold stratified cross-validation.

3.3.6.1.1 Semi-Supervised Ensemble Model Architecture

Our classification pipeline employs a semi-supervised ensemble model to spectrally differentiate sample spectra from the background substrate. This approach was designed to combine the precision of a supervised classifier with the global context of an unsupervised clustering algorithm. The final pixel classification is derived from a weighted average of two

independent components: the RF classifier (60% weight) and a PCA K-means clustering analysis (40% weight). The number of PCs was determined using the elbow method, where 99.5% of the cumulative variance was captured by the selected PCs. This dimensionality reduction facilitated subsequent clustering and classification procedures while maintaining spectral discrimination capability for downstream analysis. The RF classifier was trained on a sparse, manually annotated subset of pixels, using an enhanced feature set that includes spatial coordinates, the first 15 principal components, and engineered spectral statistics such as peak intensity, variance, and skewness. During inference, this supervised model outputs a continuous probability score for the background spectrum class. Concurrently, the unsupervised K-means component provides a binary consensus score. This is achieved by clustering the entire dataset's PCA-transformed spectra and analysing the mean spectrum of each resulting cluster for background-like characteristics. Pixels belonging to clusters identified as substrate are assigned a score of 1.0, while all others receive a score of 0.0. The final classification is determined by an ensemble score calculated as the weighted sum of these two outputs. A pixel is only designated as background if this final score exceeds a confidence threshold of 0.7, ensuring a robust consensus between the manually trained classifier and the unsupervised global clustering. The effect of each hyperparameter on the mean test accuracy during the 5-fold stratified cross-validation grid search for the Raman and FTIR datasets is illustrated in Figures S16 and S17, respectively. The final parameters for each model were selected based on achieving the highest mean F1-score, which serves as the optimal metric for balancing model recall (bias toward particle detection) and precision (minimising background misclassification).

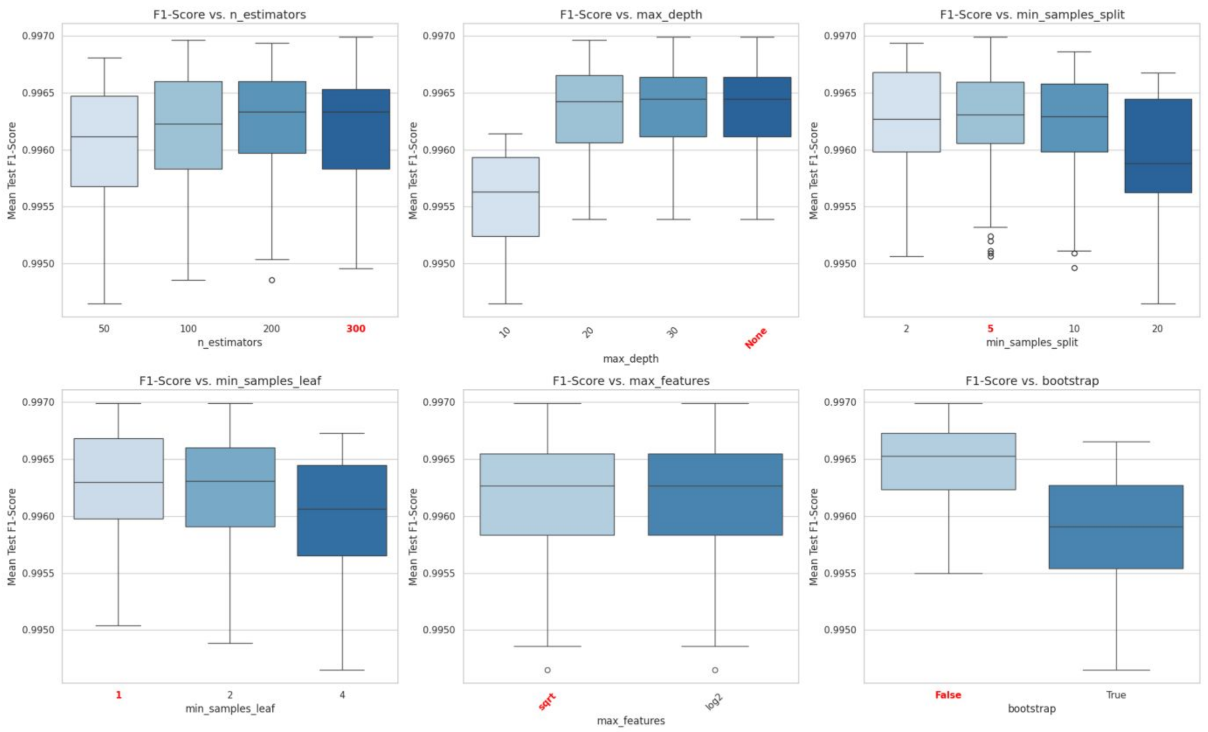

Figure S16. Hyperparameter grid search analysis for the Random Forest-K-means clustering background spectra classifier applied to Raman spectral images. These plots show the effect of each hyperparameter on model performance across a 768-combination grid search. Boxplots represent the distribution of mean test accuracy from 5-fold stratified cross-validation on the Raman dataset. The optimal hyperparameters, selected based on the highest mean F1-score, are highlighted in red along the x-axis. Specifically, the chosen parameters include: number of trees in the forest = 300 (number of estimators [n_estimators]), maximum tree depth = None (maximum depth [max_depth]), minimum samples required to split an internal node = 5 (min_samples_split), minimum samples required at a leaf node = 1 (min_samples_leaf), number of features considered for the best split = square root (max_features = sqrt), and bootstrap sampling = False (indicating that the full dataset was used to build each tree).

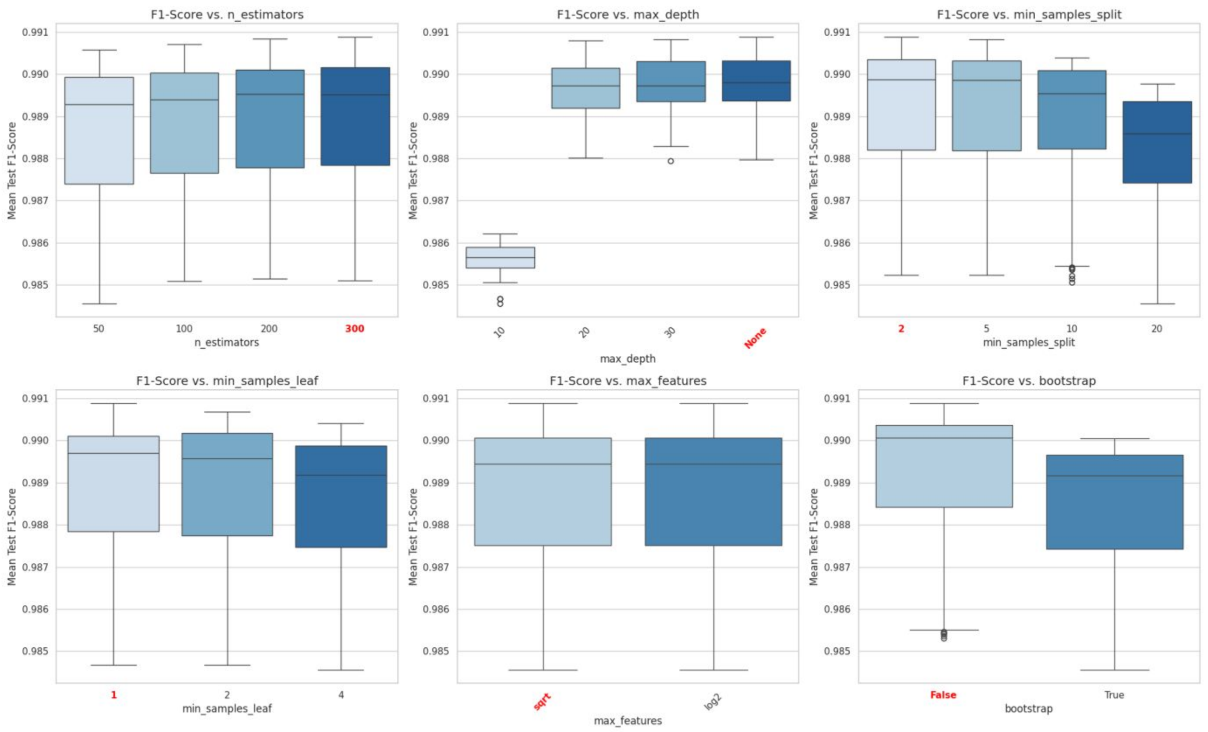

Figure S17. Hyperparameter grid search analysis for the Random Forest-K-means clustering background spectra classifier applied to FTIR spectral images. These plots show the effect of each hyperparameter on model performance across a 768-combination grid search. Boxplots represent the distribution of mean test accuracy from 5-fold stratified cross-validation on the FTIR dataset. The optimal hyperparameters, selected based on the highest mean F1-score, are highlighted in red along the x-axis. Specifically, the chosen parameters include: number of trees in the forest = 300 (number of estimators [n_estimators]), maximum tree depth = None (maximum depth [max_depth]), minimum samples required to split an internal node = 2 (min_samples_split), minimum samples required at a leaf node = 1 (min_samples_leaf), number of features considered for the best split = square root (max_features = sqrt), and bootstrap sampling = False (indicating that the full dataset was used to build each tree).

3.3.6.1.2 Feature importance for model background prediction

Following hyperparameter optimisation, a feature importance analysis was performed on the final RF classifiers to determine which input features were most influential in discriminating between substrate and sample spectra. This investigation was conducted for both the Raman and FTIR models to reveal the key drivers behind the classification decision. The top 11 features contributing to the differentiation between sample and background spectra in the RF classifier for the Raman dataset were peak intensity (0.1696), spectral skewness (0.1567), PC2 (0.1522), spectral mean (0.0979), PC1 (0.0970), total intensity (0.0738), signal-to-noise ratio (0.0531), spectral kurtosis (0.0483), PC4 (0.0303), PC5 (0.0243), and spectral variance (0.0236). For the FTIR dataset, the most important features were peak intensity (0.1768), PC1 (0.1307), spectral mean (0.1110), total intensity (0.1017), spectral variance (0.0798), PC3 (0.0460), PC4 (0.0446), PC9 (0.0351), PC11 (0.0329), signal-to-noise ratio (0.0313), and PC2 (0.0312). Overall, the RF classifier assigned higher importance to spectral features (0.6230 and 0.5289 for Raman and FTIR, respectively) compared to PCA-derived features (0.3562 and 0.4534 for Raman and FTIR), while spatial features contributed least (0.0207 and 0.0176 for Raman and FTIR).

3.3.6.1.3 Semi-supervised ensemble model inference performance

The inference performance of the final trained semi-supervised ensemble model was evaluated on the full spectral images from the test set to determine its processing speed and practical segmentation efficacy. Significant differences in image size and composition were noted between the datasets. The 53 FTIR images were highly consistent, each containing 16,384 pixels, with an average substrate coverage of 74.4 ± 15.1%. In contrast, the 25 Raman images varied significantly in size. To quantify this variability, the dataset was stratified by image size into two cohorts: large images (Group 1; n = 12; mean size, 111,100 ± 105,500 spectra) and small images (Group 2; n = 13; mean size, 960 ± 660 spectra). Substrate coverage differed substantially between cohorts, constituting 86.2 ± 6.6% of pixels in Group 1 versus 44.2 ± 15.7% in Group 2, reflecting the larger field of view in high-resolution images. On average, the total processing time (including PCA, clustering, and RF classification) for a single 16,384-pixel FTIR image was 0.907 ± 0.092 seconds. This equates to a throughput of 278,859 ± 31,352 pixels per second, with the model identifying an average of 12,195 substrate pixels per image. The RF prediction step itself took only 0.060 ± 0.010 seconds. For the larger and more variable Raman images, the average total processing time was 2.536 ± 3.456 seconds. This equates to a throughput of 209,457 ± 299,594 pixels per second, with the model identifying an average of 157,123 substrate pixels per image. The RF prediction step took 0.292 ± 0.460 seconds. The high standard deviation in Raman processing time and throughput is attributed to the extreme variance in image pixel count.

To illustrate the model's practical application, a spectral image containing cryomilled polystyrene particles on aluminium foil was used as a case study (Figure S18). Initial background identification was performed using the unsupervised PCA-based K-means clustering component. This analysis identified two distinct spectral clusters. Cluster 0, corresponding to the background region, comprised 7,148 pixels (90.8% spatial coverage) and exhibited a background score of 42.332, spectral prominence of 0.000, spectral sharpness of 0.008, and no detectable polystyrene peak presence (0.000). In contrast, Cluster 1, representing the sample region, consisted of 729 pixels (9.2% spatial coverage) and exhibited a higher composite score (57.189, where higher values indicate sample-like spectra), increased spectral prominence (0.301), greater spectral sharpness (0.015), and substantial polystyrene peak presence (1000 cm^{-1}, 0.334). This demonstrates the unsupervised component's ability to effectively segment the image into spectrally distinct sample and background regions prior to the final ensemble classification.

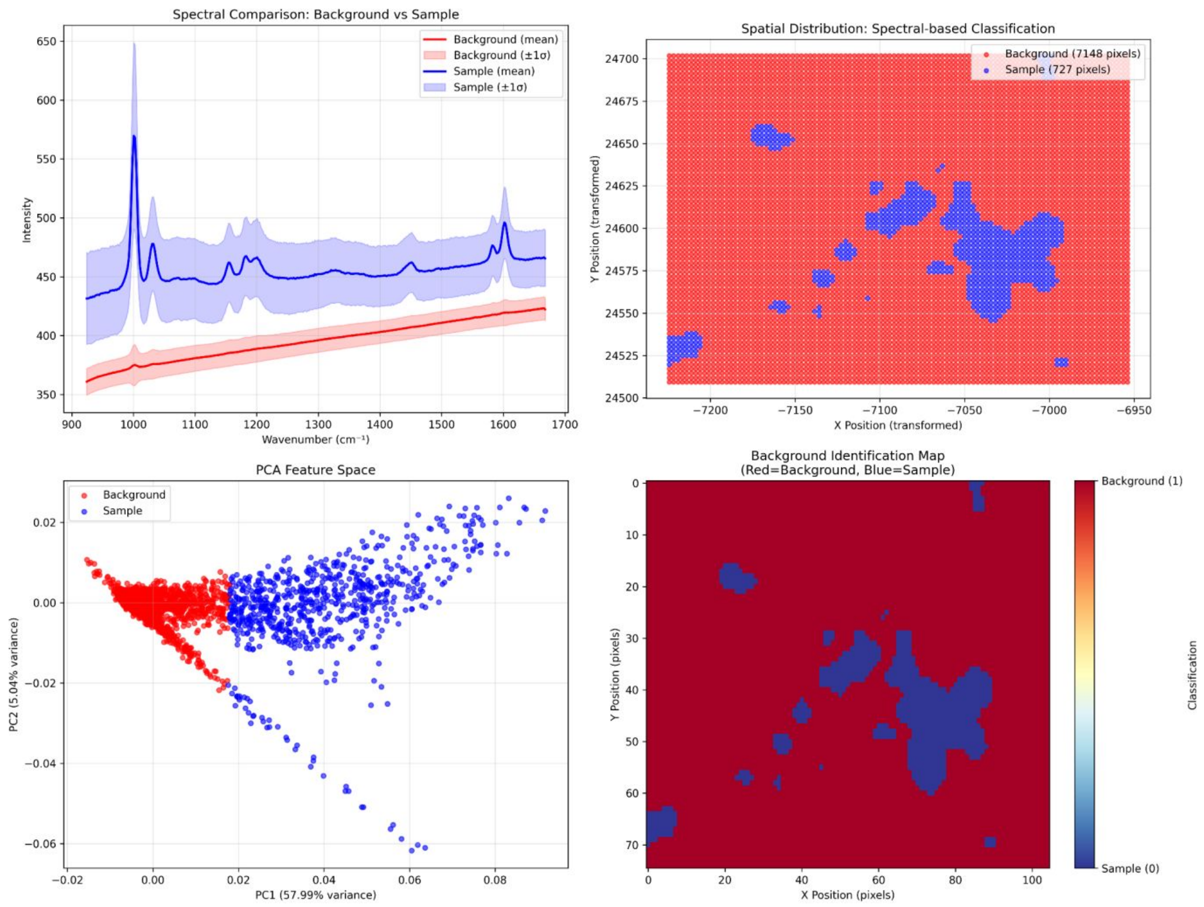

Figure S18. Inference performance of the semi-supervised ensemble model at predicting the presence of background substrate pixels using the obtained spectra. PCA-based spectral clustering for background identification in hyperspectral imaging. (A) Mean spectral profiles (±1σ) of background (red) and sample (blue) regions, showing distinct spectral features, including the presence of characteristic polystyrene peaks in the sample. (B) Spatial distribution of classified pixels: background (red, 7,148 pixels; 90.8%) and sample (blue, 729 pixels; 9.2%). (C) PCA feature space visualisation, with clusters separated along principal components PC1 and PC2, capturing 57.9% and 5.0% of variance, respectively. (D) Background identification map visualising classification results on the spectral image; red denotes background and blue denotes sample regions. This analysis demonstrates the ability of unsupervised PCA-based clustering to effectively distinguish samples from backgrounds in Raman hyperspectral datasets.

3.3.6.2 Random Forest classification grid search

The optimal hyperparameters for Raman and FTIR background detection are listed in Table S26. Cross-validation performance metrics for both modalities are summarised in Table S27.

Table S26. Hyperparameter variables for Random Forest grid-search optimisation

Parameters	Aluminium substrate (Raman)	Silver membrane substrate (FTIR)
**N estimators**	300	300
**Max depth**	None	None
**Min samples split**	5	2
**Min samples leaf**	1	1
**Max features**	sqrt	sqrt
**Bootstrap**	False	False

Table S27. Cross-validation accuracy metrics

Parameters	Raman	FTIR
**Accuracy**	99.59 ± 0.07%	98.78 ± 0.12%
**Precision**	99.62 ± 0.14%	98.85 ± 0.20%
**Recall**	99.78 ± 0.05%	99.36 ± 0.11%
**F1**	99.70 ± 0.05%	99.10 ± 0.09%
**ROC-AUC**	99.96 ± 0.03%	99.59 ± 0.09%

3.3.6.3 Representative benchmark image selection for throughput analysis

To evaluate the computational impact of the substrate subtraction workflow under realistic operating conditions, a representative benchmark image size of 400,000 spectra was selected for timing analysis. This value was chosen to approximate the typical scale of large-area environmental Raman spectral maps processed in this study, which routinely span from small exploratory scans to high-density imaging datasets used for full-sample characterisation. The selected benchmark therefore reflects a practical mid-to-large scale scenario encountered in routine application of the pipeline, rather than an extreme or synthetic edge case. This ensures that reported processing times and throughput gains are representative of real-world deployment conditions for environmental spectral imaging workflows.

3.3.7 Reference-aware adaptive clustering framework

To ensure the accuracy of final composition prediction via our PCC algorithm, a reference-aware adaptive clustering framework was developed. This framework automatically categorises reference spectra by their conformity to polymer-specific spectral signatures, identifying consensus patterns versus anomalous variants. Reference spectra were classified into three tiers. First, consensus references representing pristine polymer signatures with high inter-spectral correlation. Second, variant references capturing real-world modifications (including chemical additives and environmental degradation products). Third, complete references provide maximum spectral coverage. Divergence analysis between consensus and

variant groups identified specific wavenumbers exhibiting significant intensity differences, enabling characterisation of chemical modifications present in environmental samples. This multi-tier approach balances specificity in polymer identification with sensitivity to detect weathered microplastics.

3.3.7.1 Attenuated Total Reflection–Fourier Transform Infrared Spectroscopy (ATR-FTIR) – Plastic Spectral Library

Correlation-based clustering at an average optimal threshold of 0.896 assigned 68.1% of spectra to consensus groups and 31.9% to variant groups. Divergence analysis between consensus and variant references identified 100 spectral regions with significant intensity differences across 14 polymers (mean RMSD = 0.127 ± 0.053). PE exhibited 6 divergent peaks; PET, 7 peaks; PA, 1 peak; and PU, 2 peaks, with 62% of divergent features occurring in the fingerprint region (400–1500 cm⁻¹), 19% in the functional groups region (1500-2000 cm⁻¹), 14% in the C-H stretch (2000-3000 cm⁻¹) and the remaining 5% in the O-H/N-H stretch region (3000-4000 cm⁻¹). The spectral divergence in the mentioned polymer types can be seen in Figure S19.

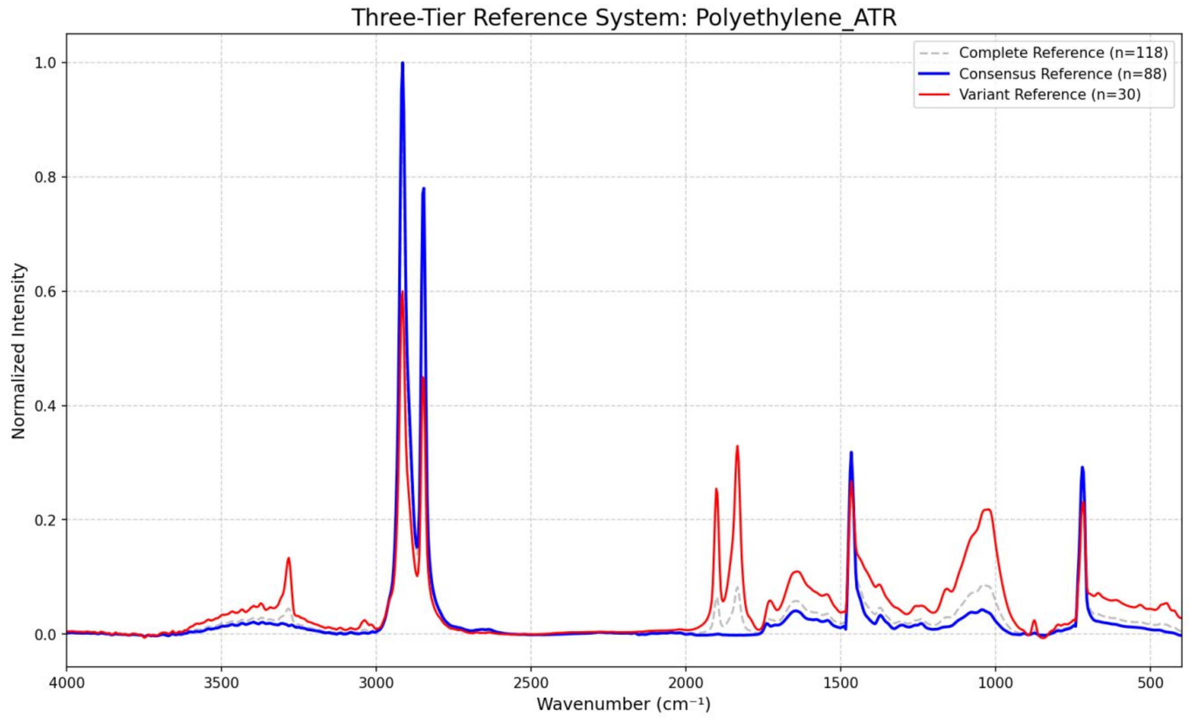

Figure S19. Visualisation of the Three-Tier Reference Framework for Spectral Library Quality Control for our FTIR polymer database. Comparative plots are shown for four example polymers from the ATR-FTIR library, such as ATR Polyethylene (PE).

The plots highlight the spectral divergence between the Consensus Reference (blue line), representing the pristine polymer signature, and the Variant Reference (red line), representing modified signatures. This divergence is particularly notable at specific wavenumbers. PA exhibits differences in the C–N stretching / skeletal vibration region (~1092 cm⁻¹; A). PE shows strong divergence in the C-H stretching region (~2915 cm⁻¹ and ~2847 cm⁻¹; B). PET exhibits differences in the fingerprint region (~670 cm⁻¹) and across its C-O ester stretches (~1240 cm⁻¹ and ~1092 cm⁻¹; C). PU shows significant divergence in the Amide II (~1532 cm⁻¹) and Amide III (~1225 cm⁻¹) bands (D). This visualisation demonstrates the framework's efficacy in

separating spectrally anomalous standards from spectrally consistent, high-quality standards. The 'Consensus Reference' is used for high-confidence matching, while the 'Variant Reference' provides signatures for identifying anomalous, i.e., containing chemical additives or environmentally degraded particles.

3.3.7.2 Raman Spectroscopy (532 nm, 633 nm, and 785 nm) – Plastic Spectral Library

Divergence analysis between these tiers identified 115 spectral regions with significant intensity differences across 14 of these polymer groups (mean RMSD = 0.086 ± 0.012). The groups with the most divergent features included Polyamide (785 nm) with 15 peaks, Acrylonitrile butadiene styrene (785 nm) with 12 peaks, Polypropylene (785 nm) with 11 peaks, Polystyrene (633 nm) with 10 peaks, and Polyethylene terephthalate (532 nm) with 9 peaks. Of these divergent features, 61% occurred in the fingerprint region (400–1500 cm^{-1}), 20% in the functional groups region (1500-2000 cm^{-1}), and the remaining 17% in the C-H/O-H stretch regions (2000-4000 cm^{-1}), with the remaining 2% distributed across minor spectral regions. The spectral divergence in the mentioned polymer types can be seen in Figure S20.

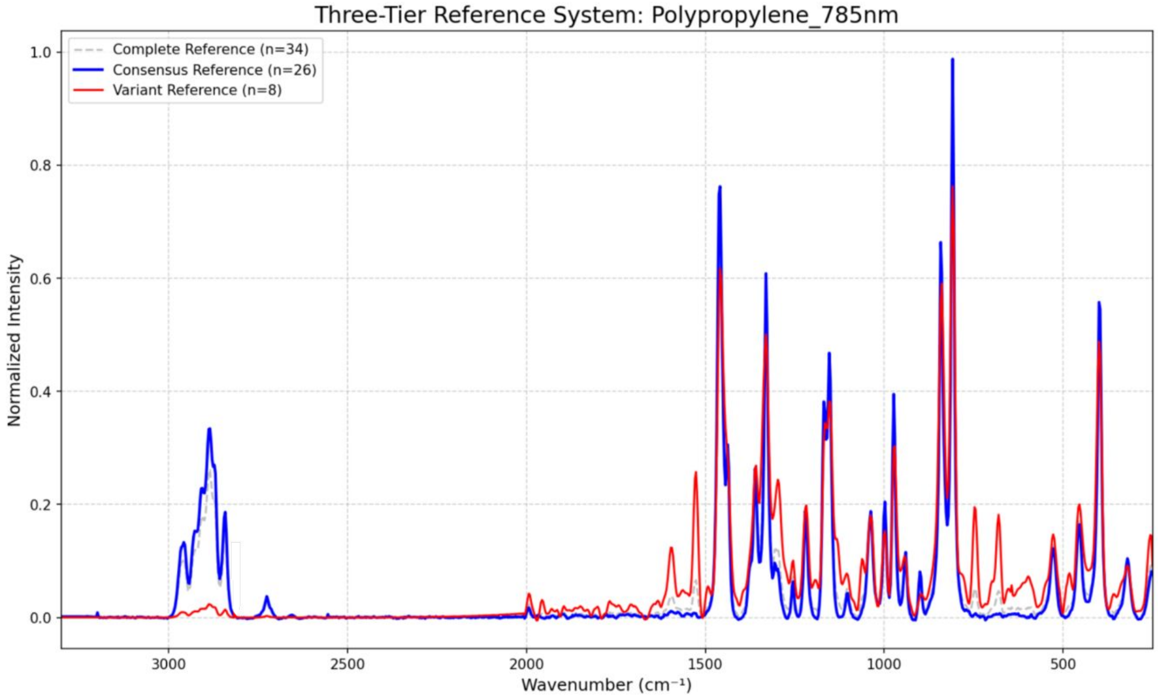

Figure S20. Visualisation of the Three-Tier Reference Framework for Spectral Library Quality Control for our Raman polymer database. Comparative plots are shown for four example polymers: (A) Acrylonitrile butadiene styrene (785 nm), (B) Polypropylene (785 nm), (C) Polystyrene (633 nm), and (D) Polyethylene terephthalate (532 nm). The plots highlight the spectral divergence between the Consensus Reference (blue line), representing the pristine polymer signature, and the Variant Reference (red line), representing modified signatures. This divergence is particularly notable at specific wavenumbers. ABS shows strong divergence in the functional group (~1523 cm^{-1}) and fingerprint (~748 cm^{-1}) regions (A). PP shows clear differences in the C-H stretch region (~2885 cm^{-1}) and at ~1526 cm^{-1} (B). PS exhibits major divergence in the high-wavenumber C-H stretch region (~3056 cm^{-1}) and at the ~995 cm^{-1} aromatic ring breathing mode (C). PET shows divergence in its C-H stretch (~2961 cm^{-1}) and carbonyl/aromatic regions (~1715-1618 cm^{-1}; D). This visualisation demonstrates the framework's efficacy in separating spectrally anomalous standards from spectrally

consistent, high-quality standards. The 'Consensus Reference' is used for high-confidence matching, while the 'Variant Reference' provides signatures for identifying anomalous particles, e.g., containing chemical additives or environmentally degraded particles.

4. Reference List

[1] Yang, Z., Nagashima, H., Murat, C. and Arakawa, H. (2025) 'An automatic method for accurate signal-to-noise ratio estimation and baseline correction of Raman spectra of environmental microplastics', Spectrochimica Acta Part A: Molecular and Biomolecular Spectroscopy, 325, p. 125061. doi: 10.1016/j.saa.2024.125061.

[2] Ghosal, S., Rogers, K.L. and Rogers, D.T. (2018) 'Molecular identification of polymers and anthropogenic particles extracted from oceanic water and fish stomach–A Raman micro-spectroscopy study', Marine Pollution Bulletin, 133, pp. 805-812.

[3] Cowger, W., Roscher, L., Jebens, H., Chamas, A., Maurer, B.D., Gehrke, L., Gerdts, G. and Primpke, S. (2024) 'Generation of macro- and microplastic databases by high-throughput FTIR analysis with microplate readers', Analytical and Bioanalytical Chemistry, 416, pp.1311–1320.

[4] Villegas-Camacho, O., Alejo-Eleuterio, R., Francisco-Valencia, I., Granda-Gutiérrez, E., Martínez-Gallegos, S. and Illescas, J. (2024) 'FTIR-Plastics: A Fourier Transform Infrared Spectroscopy dataset for the six most prevalent industrial plastic polymers', Data in Brief, 55, p. 110612. doi: 10.1016/j.dib.2024.110612.

[5] Meyers, N.; De Witte, B.; Schmidt, N.; Herzke, D.; Fuda, J.-L.; Vanavermaete, D.; Bossaer, M.; Janssen, C.; Everaert, G.; Flanders Research Institute for Agriculture, Fisheries and Food; Ghent University; Flanders Marine Institute; Aix Marseille University; Norwegian Institute for Air Research; (2024): Infrared spectra of plastic polymers weathered in the marine environment under semi-controlled conditions. Marine Data Archive.

[7] Primpke, S., Wirth, M., Lorenz, C. and Gerdts, G. (2018) 'Reference database design for the automated analysis of microplastic samples based on Fourier transform infrared (FTIR) spectroscopy', Analytical and Bioanalytical Chemistry, 410, pp.5131–5141. https://doi.org/10.1007/s00216-018-1156-x

[8] Munno, K., De Frond, H., O'Donnell, B. and Rochman, C.M. (2020) 'Increasing the accessibility for characterizing microplastics: Introducing new application-based and spectral libraries of plastic particles (SLoPP and SLoPP-E)', Analytical Chemistry, 92(3), pp.2443–2451. doi: 10.1021/acs.analchem.9b05455.

[9] Dong, M., Zhang, Q., Xing, X., Chen, W., She, Z. and Luo, Z. (2020) 'Raman spectra and surface changes of microplastics weathered under natural environments', Science of The Total Environment, 739, p. 139990. doi: 10.1016/j.scitotenv.2020.139990.

[10] Cowger, W., Steinmetz, Z., Gray, A., Munno, K., Lynch, J., Hapich, H., Primpke, S., De Frond, H., Rochman, C. and Herodotou, O. (2021) 'Microplastic spectral classification needs an open source community: Open Specy to the rescue!', Analytical Chemistry, 93(21), pp.7543–7548. doi: 10.1021/acs.analchem.1c00123.

[11x]Kowal, M., 2024. mp-classifier. [online] GitHub. Available at: https://github.com/MatthewKowal/mp-classifier [Accessed 24 February 2026].

[12] Miller, E.A., Yamahara, K.M., French, C., Spingarn, N., Birch, J.M. and Van Houtan, K.S. (2022) 'A Raman spectral reference library of potential anthropogenic and biological ocean polymers', Scientific Data, 9, 780. doi: 10.1038/s41597-022-01889-y.

[13] Azari, A., Ronsmans, S., Vanoirbeek, J.A.J., Hoet, P.H.M. and Ghosh, M. (2024) 'Challenges in Raman spectroscopy of (micro)Plastics: The interfering role of colourants', Environmental Pollution, 363(2), p. 125250. doi: 10.1016/j.envpol.2024.125250.

[14] Čerkasova, N., Enders, K., Lenz, R., Oberbeckmann, S., Brandt, J., Fischer, D., Fischer, F., Labrenz, M. and Schernewski, G. (2023) 'A public database for microplastics in the environment', Microplastics, 2(1), pp.132–146. https://doi.org/10.3390/microplastics2010010

[15] Lenz, R., Fischer, F., Arnold, M., Fernández-González, V., Moscoso Pérez, C.M., Andrade-Garda, J.M., Muniategui-Lorenzo, S. and Fischer, D. (2023) 'MicroPlastiX SpecDB: Database of Raman and ATR-FTIR spectra of weathered and biofouled polymers', [dataset] Leibniz Institute of Polymer Research and University of A Coruña. Available at: https://doi.org/10.22000/1820.

[16] Gómez-Bacab, M.T., Quezada-Campos, A.L., Patiño-Arévalo, C.D., Zepeda-Rodríguez, Z., Romero-Cano, L.A. and Zárate-Navarro, M.A. (2025) ‘Use of artificial neural networks for recycled pellets identification: Polypropylene-based composites’, Polymers, 17(17), p. 2349. doi: 10.3390/polym17172349.

[17] Jiang, J., Gou, Y., Cao, J., Jiao, F., Ma, S., Dong, D., Li, G. and Fu, X. (2025) 'Rapid Detection of Polyethylene and Polypropylene Microplastics in Soil Using Ft-Nir and Atr-Ftir Spectral Data Fusion', Available at SSRN: https://ssrn.com/abstract=5245435 or http://dx.doi.org/10.2139/ssrn.5245435

[18] Kedzierski, M., Falcou-Préfol, M., Kerros, M.E., Henry, M., Pedrotti, M.L. and Bruzaud, S. (2019) ‘A machine learning algorithm for high throughput identification of FTIR spectra: Application on microplastics collected in the Mediterranean Sea’, Chemosphere, 234, pp. 242–251. doi: 10.1016/j.chemosphere.2019.05.113.

[19] Manap, M.R.A., Imizan, M.I. bin, Dams, J.W., Rahman, N.A., Waini, F.A.Z., Mukhni, N.H. and Johari, W.L.W. (2025) ‘Dataset of microscopic images and infrared spectra of beach sediment samples from Juara, Salang, and Tulai in Tioman Island, Malaysia’, Data in Brief, 62, p. 111907. doi: 10.1016/j.dib.2025.111907.

[20] De Frond, H., Rubinovitz, R. and Rochman, C.M. (2021) 'μATR-FTIR spectral libraries of plastic particles (FLOPP and FLOPP-e) for the analysis of microplastics', Analytical Chemistry, 93(48), pp.15868–15877. doi: 10.1021/acs.analchem.1c03155.

[21] Cabernard, L., Roscher, L., Lorenz, C., Gerdts, G. and Primpke, S. (2018) 'Comparison of Raman and Fourier transform infrared spectroscopy for the quantification of microplastics in the aquatic environment', Environmental Science and Technology, 52(22), pp.13279-13288.

[22] Hogan, Ú.E., Voss, H.B., Lei, B., Bec, A.E., Feng, X. and Smith, R.D.L. (2026) 'Raman spectra for plastics identification (RaSPI) and Raman maps for plastics identification (RaMPI) datasets', Scientific Data, advance online publication.

[23] Sunil, M., Pallikkavaliyaveetil, N., N., M., Gopinath, A., Chidangil, S., Kumar, S. and Lukose, J. (2024) 'Machine learning assisted Raman spectroscopy: A viable approach for the detection of microplastics', Journal of Water Process Engineering, 60, p. 105150. doi: 10.1016/j.jwpe.2024.105150.

[24] Zhang, Y., Ma, J., Sun, Z. and Du, J. (2025) 'Efficient identification of microplastics based on interpretable deep learning–surface-enhanced Raman scattering', Journal of Instrumental Analysis, 44(8), pp. 1557–1567. doi: 10.12452/j.fxcsxb.250331247.

[25] Ren, L., Liu, S., Huang, S., Wang, Q., Lu, Y., Song, J., Guo, J. and Guo, J. (2023) 'Identification of microplastics using a convolutional neural network based on micro-Raman spectroscopy', Talanta, 260, p. 124611. doi: 10.1016/j.talanta.2023.124611.

[26] Lei, B., Bissonnette, J.R., Hogan, Ú.E., Bec, A.E., Feng, X. and Smith, R.D.L. (2022) 'Customizable machine-learning models for rapid microplastic identification using Raman microscopy', Analytical Chemistry, 94(49), pp. 17011–17019. doi: 10.1021/acs.analchem.2c02451.

[27] Huang, W., Chen, J., Xiong, H., Tan, T., Wang, G., Liu, K., Chen, C. and Gao, X. (2025) 'Improved neural networks for the classification of microplastics via inferior quality Raman spectra', Talanta, 289, p. 127756. doi: 10.1016/j.talanta.2025.127756.

[28] He, K., Zhang, X., Ren, S. and Sun, J. (2016) 'Deep residual learning for image recognition', Proceedings of the IEEE conference on computer vision and pattern recognition, pp.770-778.

[29] Yang, J., Xu, J., Zhang, X., Wu, C., Lin, T. and Ying, Y. (2019) 'Deep learning for vibrational spectral analysis: Recent progress and a practical guide', Analytica Chimica Acta, 1081, pp.6-17.

[30] Bell, I.M., Clark, R.J.H. and Gibbs, P.J. (1997) Raman spectroscopic library of natural and synthetic pigments (pre-approximately 1850 AD). Spectrochimica Acta Part A: Molecular and Biomolecular Spectroscopy, 53A(12), pp.2159–2179. https://doi.org/10.1016/s1386-1425(97)00140-6

[31] Bērziņš, K., Sales, R.E., Barnsley, J.E., Walker, G., Fraser-Miller, S.J. and Gordon, K.C. (2020) 'Low-wavenumber Raman spectral database of pharmaceutical excipients', Vibrational Spectroscopy, 107, 103021.

[32] Doughty, D.C. and Hill, S.C. (2020) 'Raman spectra of atmospheric aerosol particles: Clusters and time-series for a 22.5 hr sampling period', Journal of Quantitative Spectroscopy and Radiative Transfer, 248, p. 106907.

[33] Flanagan, A.R. and Glavin, F.G. (2025) Open-source Raman spectra of chemical compounds for active pharmaceutical ingredient development. Scientific Data, 12, Article 498. https://doi.org/10.1038/s41597-025-0498-x

[34] Li, D., Sheerin, E.D., Shi, Y., Xiao, L., Yang, L., Boland, J.J. and Wang, J.J. (2022) ‘Alcohol pretreatment to eliminate the interference of micro additive particles in the identification of microplastics using Raman spectroscopy’, *Environmental Science & Technology*, 56(17), pp. 11947–11955.

[35] Lim, J., Seo, J. and Shin, D. (2025) ‘Reducing spectral confusion in microplastic analysis: a U-Net deep learning approach’, *Analytical Chemistry*, 97(34), pp. 18432–18443.

[36] Neo, E.R.K., Low, J.S.C., Goodship, V. and Debattista, K. (2023) 'Deep learning for chemometric analysis of plastic spectral data from infrared and Raman databases', Resources, Conservation and Recycling, 188, p. 106718. doi: 10.1016/j.resconrec.2022.106718.

[37] Cooman, T., Stewart, J., Bramble, S.K. and Barden, C.A. (2022) 'Implementing machine learning for the identification and classification of compounds and mixtures in portable Raman instruments', Chemical Physics Letters, 799, 139657. doi: 10.1016/j.cplett.2021.139657.

[38] Qin, Y., Qiu, J., Tang, N., He, Y. and Fan, L. (2024) 'Deep learning analysis for rapid detection and classification of household plastics based on Raman spectroscopy', Spectrochimica Acta Part A: Molecular and Biomolecular Spectroscopy, 309, p. 123854.

[39] Levermore, J.M., Smith, T.E.L., Kelly, F.J. and Wright, S.L. (2020) 'Detection of microplastics in ambient particulate matter using Raman spectral imaging and chemometric analysis', Analytical Chemistry, 92(13), pp.8732–8740. doi: 10.1021/acs.analchem.0c00922.

[40] Mayorova, O.A., Saveleva, M.S., Bratashov, D.N. and Prikhozhdenko, E.S. (2024) 'Combination of Machine Learning and Raman Spectroscopy for Determination of the Complex of Whey Protein Isolate with Hyaluronic Acid', Polymers (Basel), 16(5), p.666. doi: 10.3390/polym16050666.

[41] Zhang, W., Giang, C.M., Cai, Q., Badie, B., Sheng, J. and Li, C. (2023) 'Using random forest for brain tissue identification by Raman spectroscopy', Machine Learning: Science and Technology, 4(4), p.045053. doi: 10.1088/2632-2153/ad1349.

[42] Seifert, S. (2020) 'Application of random forest based approaches to surface-enhanced Raman scattering data', Scientific Reports, 10, p.5436. doi: 10.1038/s41598-020-62469-0.

[43] Chen, L., Sabonchi, A.K.S. and Nanehkaran, Y.A. (2024) 'COVID-19 pandemic microplastics environmental impacts predicted by deep random forest (DRF) predictive model', Environmental Sciences Europe, 36(193). doi: 10.1186/s12302-024-01019-z.

[44] Coca-Lopez, N. (2024) 'An intuitive approach for spike removal in Raman spectra based on peaks’ prominence and width', Analytica Chimica Acta, 1295, p.342312. doi: 10.1016/j.aca.2024.342312.

4.1 Additional References

Alex, S.A., Nayahi, J.J.V. and Kaddoura, S. (2024) 'Deep convolutional neural networks with genetic algorithm-based synthetic minority over-sampling technique for improved imbalanced data classification', Applied Soft Computing, 156, p.111491. doi:10.1016/j.asoc.2024.111491.

Araujo, C.F., Nolasco, M.M., Ribeiro, A.M.P. and Ribeiro-Claro, P.J.A. (2018) 'Identification of microplastics using Raman spectroscopy: Latest developments and future prospects', Water Research, 142, pp.426–440. doi: 10.1016/j.watres.2018.05.060.

Barton, S.J. and Hennelly, B.M. (2019) 'An algorithm for the removal of cosmic ray artefacts in spectral data sets', Applied Spectroscopy, 73(8). doi: 10.1177/0003702819839098.

Bell, I.M., Clark, R.J.H. and Gibbs, P.J. (1997) Raman spectroscopic library of natural and synthetic pigments (pre-approximately 1850 AD). Spectrochimica Acta Part A: Molecular and Biomolecular Spectroscopy, 53A(12), pp.2159–2179. https://doi.org/10.1016/s1386-1425(97)00140-6

Bergstra, J. and Bengio, Y. (2012) 'Random search for hyper-parameter optimization', Journal of Machine Learning Research, 13, pp.281–305.

Blake, N., Gaifulina, R., Griffin, L.D., Bell, I.M. and Thomas, G.M.H. (2022) 'Machine learning of Raman spectroscopy data for classifying cancers: A review of the recent literature', Diagnostics, 12(6), p.1491. doi: 10.3390/diagnostics12061491.

Borawar, L. and Kaur, R. (2023) 'ResNet: Solving Vanishing Gradient in Deep Networks', In: Mahapatra, R.P., Peddoju, S.K., Roy, S. and Parwekar, P. (eds.) Proceedings of International Conference on Recent Trends in Computing. Singapore: Springer, pp.235-247. doi: 10.1007/978-981-19-8825-7_21.

Brandt, J., Mattsson, K. and Hassellöv, M. (2021) 'Deep Learning for Reconstructing Low-Quality FTIR and Raman Spectra—A Case Study in Microplastic Analyses', Analytical Chemistry, 93(49).

Brooks, J.M., Boyer, J.J., Haberstroh, C.J. and Arias, M.E. (2024) 'Settling velocities of environmentally weathered plastic fibers from the Mekong River in Southeast Asia', ACS ES&T Water, 4(4), pp.1556–1563. doi: 10.1021/acsestwater.3c00649.

Caggiani, M.C., Cosentino, A. and Mangone, A. (2016) Pigments Checker version 3.0, a handy set for conservation scientists: A free online Raman spectra database. Microchemical Journal, 129, pp.123–132. https://doi.org/10.1016/j.microc.2016.06.020

Cai, Y., Lin, Y., Cai, H., and Ni, H. (2025) Deep learning in vibrational spectroscopy: Benefits, limitations, and recent progress. Journal of the Chinese Chemical Society, 72(6), 611-626. https://doi.org/10.1002/jccs.70031

Chen, H., Xu, W. and Broderick, N.G.R. (2018) 'An adaptive and fully automated baseline correction method for Raman spectroscopy based on morphological operations and mollification', Applied Spectroscopy. doi: 10.1177/0003702818811688.

Chen, W. and Shi, K. (2021) 'Multi-scale attention convolutional neural network for time series classification', Neural Networks, 136, pp.126–140. doi: 10.1016/j.neunet.2021.01.002.

Chen, Y., Wen, D., Pei, J., Fei, Y., Ouyang, D., Zhang, H. and Luo, Y. (2020) 'Identification and quantification of microplastics using Fourier-transform infrared spectroscopy: Current

status and future prospects', Current Opinion in Environmental Science & Health, 18, pp.14–19. doi: 10.1016/j.coesh.2020.05.009.

Liu, Z., Yang, Y., Huang, M. and Zhu, Q. (2023) ‘Spatially Offset Raman Spectroscopy Combined with Attention-Based LSTM for Freshness Evaluation of Shrimp’, *Sensors*, 23(5), p. 2827. doi: 10.3390/s23052827.

Clough, M.E., Ochoa Rivera, E., Parham, R.L., Ault, A.P., Zimmerman, P.M., McNeil, A.J. and Tewari, A. (2024) 'Enhancing confidence in microplastic spectral identification via conformal prediction', Environmental Science & Technology, 58(49), pp.21740–21749. doi: 10.1021/acs.est.4c05167.

Cowger, W., Gray, A., Christiansen, S.H., DeFrond, H., Deshpande, A.D., Hemabessiere, L., Lee, E., Mill, L., Munno, K., Ossmann, B.E., Pittroff, M., Rochman, C., Sarau, G., Tarby, S. and Primpke, S. (2020) 'Critical review of processing and classification techniques for images and spectra in microplastic research', Applied Spectroscopy, 74(9), pp. 989–1010.

De Frond, H., Thornton Hampton, L., Kotar, S., Gesulga, K., Matuch, C.M., Lao, W., Weisberg, S.B. and Wong, C.S. (2022) 'Monitoring microplastics in drinking water: An interlaboratory study to inform effective methods for quantifying and characterizing microplastics', Chemosphere, 298, 134282.

Dong, M., She, Z., Xiong, X., Ouyang, G., and Luo, Z. (2022) Automated analysis of microplastics based on vibrational spectroscopy: Are we measuring the same metrics? Analytical and Bioanalytical Chemistry, 414(11), 3359–3372.

Elizar, E., Zulkifley, M.A., Muharar, R., Zaman, M.H.M. and Mustaza, S.M. (2022) 'A review on multiscale-deep-learning applications', Sensors, 22(19), p.7384. doi: 10.3390/s22197384.

Elnahas, A., Gray, A., Lee, J., AlAmiri, N., Pokhrel, N., Allen, S. and Foroutan, H. (2025) 'Atmospheric deposition of microplastics in South Central Appalachia in the United States', ACS ES&T Air, 2(1), pp.64–72. doi: 10.1021/acsestair.4c00189.

Faltynkova, A., Johnsen, G. and Wagner, M. (2021) Hyperspectral imaging as an emerging tool to analyze microplastics: A systematic review and recommendations for future development. Micropl. & Nanopl. 1, 13.

Fremout, W. and Saverwyns, S. (2012) 'Identification of synthetic organic pigments: the role of a comprehensive digital Raman spectral library', Journal of Raman Spectroscopy, 43, pp.1536-1544. doi:10.1002/jrs.4054

Geovanna Zea Cobos, A., Amón, J., León, E., and Caballero, P. (2023) Standardization of FTIR-Based Methodologies for Microplastics Detection in Drinking Water: A Meta-Analysis Indeed and Practical Approach. Water, 16(22), 3170.

Giordani, S., Huber, M.J., Jüngling, I.S., Zattoni, A., Roda, B., Reschiglian, P., Marassi, V. and Ivleva, N.P. (2025) 'Online coupling of field-flow fractionation with Raman microspectroscopy enables the advanced study of nanoplastics directly in food', Analytical Chemistry, 98(1), pp. 488–496. doi: 10.1021/acs.analchem.5c05137.

Groom, D.E. (2002) 'Cosmic Rays and Other Nonsense in Astronomical CCD Imagers', Experimental Astronomy, 14(1), pp. 45-55. doi: 10.1023/A:1025603703901.

Han, Y., Wei, C., Zhou, R., Hong, Z., Zhang, Y. and Yang, S. (2020) 'Combining 3D-CNN and Squeeze-and-Excitation Networks for remote sensing sea ice image classification', Mathematical Problems in Engineering, 2020, p. 8065396. doi: 10.1155/2020/8065396.

Heid, E., McGill, C.J., Vermeire, F.H. and Green, W.H. (2023) 'Characterizing uncertainty in machine learning for chemistry', Journal of Chemical Information and Modeling, 63(13), pp.4012–4029. doi:10.1021/acs.jcim.3c00373.

Hendrycks, D. and Gimpel, K. (2016) Gaussian Error Linear Units (GELUs). arXiv preprint arXiv:1606.08415 [cs.LG]. Available at: https://doi.org/10.48550/arXiv.1606.08415 (Accessed: 23 March 2026).

Ho, C.-S., Jean, N., Hogan, C.A., Blackmon, L., Jeffrey, S.S., Holodniy, M., Banaei, N., Saleh, A.A.E., Ermon, S. and Dionne, J. (2019) ‘Rapid identification of pathogenic bacteria using Raman spectroscopy and deep learning’, *Nature Communications*, 10, Article 4927.

Hu, J., Shen, L., Albanie, S., Sun, G. and Wu, E. (2019) Squeeze-and-Excitation Networks. arXiv preprint arXiv:1709.01507. doi: 10.48550/arXiv.1709.01507.

Huber, M.J., Zada, L., Ivleva, N.P. and Ariese, F. (2024) ‘Multi-parameter analysis of nanoplastics in flow: taking advantage of high sensitivity and time resolution enabled by stimulated Raman scattering’, *Analytical Chemistry*, 96(22), pp. 8949–8955.

Ivleva, N. P. (2021). Chemical analysis of microplastics and nanoplastics: Challenges, advanced methods, and perspectives. Chemical Reviews, 121(19), 11886–11936.

Jacob, O., Stefaniak, E. A., Seghers, J., La Spina, R., Schirinzi, G. F., Chatzipanagis, K., Held, A., Emteborg, H., Koeber, R., Elsner, M. and Ivleva, N. P. (2024) 'Towards a reference material for microplastics’ number concentration—case study of PET in water using Raman microspectroscopy', Analytical and Bioanalytical Chemistry, 416, pp.3045–3058.

James, B.D., Medvedev, A.V., Makarov, S.S., Nelson, R.K., Reddy, C.M. and Hahn, M.E. (2024) 'Moldable plastics (polycaprolactone) can be acutely toxic to developing zebrafish and activate nuclear receptors in mammalian cells', ACS Biomaterials Science & Engineering, 10(8), pp.5237–5251. doi: 10.1021/acsbiomaterials.4c00693.

Jung, E.S., Choe, J.H., Kim, J.S., Ahn, D.W., Yoo, J., Choi, T.M. and Pyo, S.G. (2024) 'Quantitative Raman analysis of microplastics in water using peak area ratios for concentration determination', npj Clean Water, 7, p. 104. doi: 10.1038/s41545-024-00397-4.

Kapukotuwa, G.K., Jayasena, N., Weerakoon, K.C., Abayasekara, C.L. and Rajakaruna, R.S. (2025) 'Microplastic pollution of stream water and sediment in a tributary of a major drinking water supplying river in Sri Lanka', ACS ES&T Water, 5(5), pp.2155–2168. doi: 10.1021/acsestwater.4c00936.

Chen, T. and Baek, S.-J. (2023) ‘Library-Based Raman Spectral Identification Using Multi-Input Hybrid ResNet’, *ACS Omega*, 8(40), pp. 37482–37489. doi: 10.1021/acsomega.3c05780.

Koelmans, A.A., Mohamed Nor, N.H., Hermsen, E., Kooi, M., Mintenig, S.M. and De France, J. (2019) 'Microplastics in freshwaters and drinking water: Critical review and assessment of data quality', Water Research, 155, pp.410–422.

Kunapuli, G. (2023) Ensemble Methods for Machine Learning. Manning Publications.

Lafuente, B., Downs, R. T., Yang, H. and Stone, N. (2015) 'The power of databases: the RRUFF project', Highlights in Mineralogical Crystallography, T. Armbruster and R. M. Danisi (eds.), Berlin, Germany, W. De Gruyter, pp.1–30.

Langknecht, T., Lao, W., Wong, C. S., Kotar, S., El Khatib, D., Robinson, S., Burgess, R. M. and Ho, K. T. (2023) 'Comparison of two procedures for microplastics analysis in sediments based on an interlaboratory exercise', Chemosphere, 313, 137479. https://doi.org/10.1016/j.chemosphere.2022.137479

Levermore, J.M., Smith, T.E.L., Kelly, F.J. and Wright, S.L. (2020) 'Detection of microplastics in ambient particulate matter using Raman spectral imaging and chemometric analysis', Analytical Chemistry, 92(13), pp.8732–8740. doi: 10.1021/acs.analchem.0c00922.

Li, H., Xu, S., Teng, J., Jiang, X., Zhang, H., Qin, Y., He, Y. and Fan, L. (2025) 'Deep learning assisted ATR-FTIR and Raman spectroscopy fusion technology for microplastic identification', Microchemical Journal, 212, p. 113224.

Chen, Z., Xie, Y., Wu, Y., Lin, Y., Tomiya, S. and Lin, J. (2024) ‘An interpretable and transferrable vision transformer model for rapid materials spectra classification’, *Digital Discovery*, 3(2), pp. 493–503. doi: 10.1039/D3DD00198A.

Lusher, A.L., Bråte, I.L.N., Munno, K., Hurley, R.R. and Welden, N.A. (2020) 'Is it or isn’t it: The importance of visual classification in microplastic characterization', Applied Spectroscopy, 74(9), pp.1139–1153.

Ma, J., Zuo, M., Wu, B. and Wang, W. (2025) 'An automated baseline correction method for Raman spectra based on piecewise polynomial fitting with adaptive window', Journal of Raman Spectroscopy, 56(4), pp.337-344. doi: 10.1002/jrs.6772.

Madan, S., Henry, T., Dozier, J., Ho, H., Bhandari, N., Sasaki, T., Durand, F., Pfister, H. and Boix, X. (2022) ‘When and how convolutional neural networks generalize to out-of-distribution category–viewpoint combinations’, *Nature Machine Intelligence*, 4, pp. 146–153. doi: 10.1038/s42256-021-00437-5.

Merrill, G.B., Hermabessiere, L., Rochman, C.M. and Nowacek, D.P. (2023) 'Microplastics in marine mammal blubber, melon, & other tissues: Evidence of translocation', Environmental Pollution, 335, p. 122252. doi: 10.1016/j.envpol.2023.122252.

Mintenig, S.M., Kooi, M., Erich, M.W., Primpke, S., Redondo-Hasselerharm, P.E., Dekker, S.C., Koelmans, A.A. and van Wezel, A.P. (2020) 'A systems approach to understand

microplastic occurrence and variability in Dutch riverine surface waters', Water Research, 176, 115723.

Noh, S.-H. (2021) 'Performance Comparison of CNN Models Using Gradient Flow Analysis', Informatics, 8(3), p.53. doi: 10.3390/informatics8030053.

Pelletier, M. (2003) 'Quantitative Analysis Using Raman Spectrometry', Applied Spectroscopy, 57(1), pp. 20A-40A. doi: 10.1366/000370203321165147.

Primpke, S., Cross, R.K., Mintenig, S.M., Simon, M., Vianello, A., Gerdts, G. and Vollertsen, J. (2020) 'Toward the systematic identification of microplastics in the environment: Evaluation of a new independent software tool (siMPle) for spectroscopic analysis', Applied Spectroscopy, 74(9), pp.1127–1138. doi: 10.1177/0003702820917760.

Qiu, M., Zheng, S., Tang, L., Hu, X., Xu, Q., Zheng, L. and Weng, S. (2022) 'Raman Spectroscopy and Improved Inception Network for Determination of FHB-Infected Wheat Kernels', Foods, 11(4), p. 578. doi: 10.3390/foods11040578.

Rathikannu, S., Gautam, S., Joshi, S.K., Katharine, P., Mithra, K.E., Banusaranya, P., Amudhavarshini, V.M., Gayatri, R. and Ho, C.-H. (2025) 'FTIR based assessment of microplastic contamination in soil water and insect ecosystems reveals environmental and ecological risks', Scientific Reports, 15, 28615.

Rosales-Martínez, O., Granda-Gutiérrez, E.E., García-Hernández, R.A., Alejo-Eleuterio, R. and Flores-Fuentes, A.A. (2025) 'Spectral derivatives improve FTIR-based machine learning classification of plastic polymers', Modelling, 6(4), 115.

Smolen, J., Moore, G., Perez, N. D., Wooley, K. L. (2025). Microplastic Micro-FTIR Spectra. figshare. Dataset. https://doi.org/10.6084/m9.figshare.29911817.v1

Song, Y.K., Hong, S.H., Eo, S. and Shim, W.J. (2021) 'A comparison of spectroscopic analysis methods for microplastics: Manual, semi-automated, and automated Fourier transform infrared and Raman techniques', Marine Pollution Bulletin, 173, 113101.

Stockin, K.A., Pantos, O., Betty, E.L., Pawley, M.D.M., Doake, F., Masterton, H., Palmer, E.I., Perrott, M.R., Nelms, S.E. and Machovsky-Capuska, G.E. (2021) 'Fourier transform infrared (FTIR) analysis identifies microplastics in stranded common dolphins (Delphinus delphis) from New Zealand waters', Marine Pollution Bulletin, 173(Pt B), p. 113084. doi: 10.1016/j.marpolbul.2021.113084.

Szegedy, C., Vanhoucke, V., Ioffe, S., Shlens, J. and Wojna, Z. (2016) 'Rethinking the inception architecture for computer vision', Proceedings of the IEEE Conference on Computer Vision and Pattern Recognition (CVPR), pp.2818-2826.

Vaswani, A., Shazeer, N., Parmar, N., Uszkoreit, J., Jones, L., Gomez, A.N., Kaiser, Ł. and Polosukhin, I. (2017) 'Attention is all you need', Advances in Neural Information Processing Systems, 30, pp.5998-6008.

Weisser, J., Pohl, T., Ivleva, N.P., Hofmann, T.F. and Glas, K. (2022) 'Know what you don’t know: assessment of overlooked microplastic particles in FTIR images', Microplastics, 1(3), pp. 359–376. doi: 10.3390/microplastics1030027.

Wright, S.L., Levermore, J.M. and Kelly, F.J. (2019) 'Raman Spectral Imaging for the Detection of Inhalable Microplastics in Ambient Particulate Matter Samples', Environmental Science & Technology, 53(15), pp. 8947–8956. doi: 10.1021/acs.est.8b06663.

Xie, L., Luo, S., Liu, Y., Ruan, X., Gong, K., Ge, Q., Li, K., Valev, V.K., Liu, G. and Zhang, L. (2023) 'Automatic identification of individual nanoplastics by Raman spectroscopy based on machine learning', Environmental Science and Technology, 57(46). https://doi.org/10.1021/acs.est.3c03045

Xie, L., Ma, M., Ge, Q., Liu, Y. and Zhang, L. (2025) 'Machine learning advancements and strategies in microplastic and nanoplastic detection', Environmental Science & Technology, 59(18). doi: 10.1021/acs.est.4c11888.

Yang, Z. and Arakawa, H. (2023) 'A double sliding-window method for baseline correction and noise estimation for Raman spectra of microplastics', Marine Pollution Bulletin, 190, p. 114887. doi: 10.1016/j.marpolbul.2023.114887.

Yun, J. (2024) Mitigating gradient overlap in deep residual networks with gradient normalization for improved non-convex optimization. arXiv preprint arXiv:2410.21564. Available at: https://arxiv.org/abs/2410.21564.

Zeng, G., Ma, Y., Du, M., Chen, T., Lin, L., Dai, M., Luo, H., Hu, L., Zhou, Q. and Pan, X. (2024) 'Deep convolutional neural networks for aged microplastics identification by Fourier transform infrared spectra classification', Science of The Total Environment, 913, p. 169623.

Zhang, F., Li, K. and Ren, Z. (2024) 'Improving Adversarial Robustness of Ensemble Classifiers by Diversified Feature Selection and Stochastic Aggregation', Mathematics, 12(6), p. 834. doi: 10.3390/math12060834.

Zhou, C., Liu, Y., An, X., Xu, X. and Wang, H. (2025) ‘Optimization of deep learning architecture based on multi-path convolutional neural network algorithm’, *Scientific Reports*, 15, p. 19532. doi: 10.1038/s41598-025-03765-3.

Zhu, S., Yan, Y., Wei, L., Li, Y., Mao, T., Dai, X. and Du, R. (2024) SECA-Net: Squeezed-and-excitated contextual attention network for medical image segmentation. Biomedical Signal Processing and Control, 97, 106704. https://doi.org/10.1016/j.bspc.2024.106704

Zhu, Z., Parker, W. and Wong, A. (2021) 'PlasticNet: Deep learning for automatic microplastic recognition via FT-IR spectroscopy', Journal of Computational Vision and Imaging Systems, 6(1), pp.1-3.

Zou, H.-H., He, P.-J., Peng, W., Lan, D.-Y., Xian, H.-Y., Lü, F. and Zhang, H. (2025) 'Rapid detection of colored and colorless macro- and micro-plastics in complex environment via near-infrared spectroscopy and machine learning', Journal of Environmental Sciences, 147, pp.512-522.
